# A magic methyl effect in the active site of a methyltransferase ribozyme

**DOI:** 10.1038/s41467-026-75575-8

**Published:** 2026-07-17

**Authors:** Jana Aupič, Hsuan-Ai Chen, Carolin P. M. Scheitl, Claudia Höbartner, Alessandra Magistrato

**Affiliations:** 1 CNR—Istituto Officina dei Materiali (IOM) c/o Scuola Internazionale Superiore di Studi Avanzati (SISSA), Trieste, Italy; 2 Institute of Organic Chemistry, Julius-Maximilians-Universität Würzburg, Würzburg, Germany; 3 Center for Nanosystems Chemistry, Julius-Maximilians-Universität Würzburg, Würzburg, Germany; 4 Cluster for Nucleic Acid Sciences and Technologies—NUCLEATE, Würzburg, Germany

**Keywords:** RNA, Ribozymes, Computational chemistry, Chemical modification, X-ray crystallography

## Abstract

The chemical richness of RNAs is greatly enhanced by post-transcriptional modifications with RNA methylation as the most prominent type. RNA modifications modulate the stability, folding and interaction pattern of RNA molecules. Furthermore, emerging data suggests RNA modifications also directly regulate the activity of catalytic RNA molecules, i.e., ribozymes. Here, we employ classical and hybrid quantum-classical (QM/MM) molecular dynamics (MD) simulations to investigate the reaction mechanism of an artificial methyltransferase ribozyme MTR1. Importantly, we pinpoint how 2’-*O*-methylations of active site nucleotides synergistically enhance ribozyme activity by reducing the conformational flexibility of the ribose rings and rigidifying the active site. Finally, the herein reported crystal structure of the modified MTR1, solved at 2.6 Å resolution, validates the results of our simulations. Taken together, our work supports the purported central role of modified RNA for early RNA catalysis and may guide rational design of more efficient ribozymes.

## Abstract (web summary)

Emerging data indicate RNA modifications are important regulators of ribozyme activity. Here, the authors show how 2’-*O*-methylation of active site nucleotides enhances catalytic activity by reducing ribose rings’ conformational flexibility.

## Introduction

RNA is an essential biomolecule that mediates a variety of cellular functions. RNA molecules are the backbone of DNA transcription and subsequent protein synthesis, and thus central facilitators and regulators of gene expression^1,2^. Additionally, RNA molecules also act as catalysts^3–5^. Hitherto discovered natural RNA-based enzymes, called ribozymes, are able of catalysing a limited assortment of chemical reactions, concerning phosphodiester bond breaking and formation^3–5^. However, it is believed that in the evolutionary past RNA was able to promote a much wider set of chemical conversions^6–8^. The development of synthetic ribozymes by in vitro selection methods confirmed ribozymes can, at least in the laboratory, catalyse diverse chemical reactions such as Diels-Alder reaction, aldol condensation, aminoacylation, Michael addition and RNA alkylation^9–14^.

**Fig. 1:**
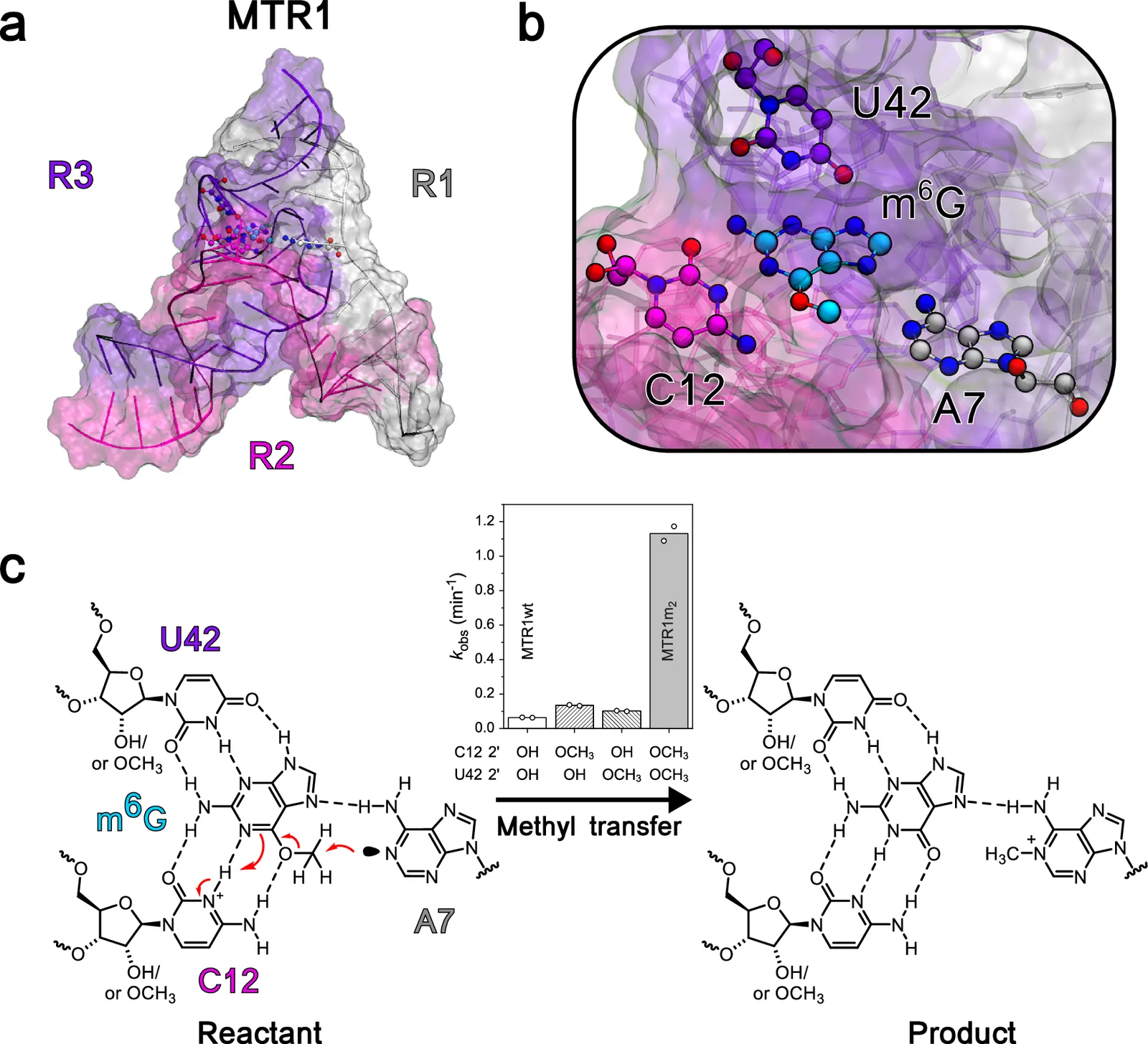
MTR1 is an artificial ribozyme that directs site-specific methylation of the adenosine residue. **a** Molecular dynamics simulation were based on the crystal structure of split MTR1 (PDB code: 7Q7X). RNA strands R1, R2 and R3 are shown as grey, pink, and violet ribbons. Active site atoms are shown as balls and sticks. **b** Close-up of the MTR1 active site. Methyl group donor *O*^6^-methylguanine (m^6^G) is held in place through formation of hydrogen bonds with protonated C12 and U42. A7 is the target of the methylation reaction. **c** Methylation reaction occurs in two steps. First, the proton is transferred from C12 to m^6^G, followed by methyl group transfer to A7 in the rate-limiting step. Modifications of the C12 and U42 ribose ring at the 2’ position were shown to affect the rate of the methylation reaction; individual data points are shown for *n* = 2 independent replicates^19^.

Ribozymes facilitating site-specific RNA modification recently gained enhanced attention, with several reports on methyltransferase ribozymes^15–17^. An early example of artificial ribozymes is MTR1, which mimics natural RNA methyltransferase proteins and was developed in the Höbartner group^18,19^. MTR1 catalyses methyl group deposition on a specific adenosine residue, utilizing the small-molecule *O*^6^-methyl guanine (m^6^G) as the methyl source, to yield 1-methyladenosine (m^1^A)-modified RNA and unmethylated guanine. MTR1 contains two binding arms (R2 and R3) that recognize the substrate RNA strand (R1) via Watson-Crick complementarity (Fig. 1a). By engineering the nucleotide sequence of the binding arms, MTR1 specificity can be readily tuned. The crystal structure of MTR1 in complex with its cofactor revealed MTR1 assumes a 3-helix junction structure with the catalytic core located at its centre^19,20^. There, guanine is held in place by extensive hydrogen bonding to C12 and U42, forming a base triple close to m^1^A that is located in the same plane (Fig. 1b). This base triple is further stabilized by base stacking on both upper and lower face. The arrangement of active site nucleotides suggests the protonated C12 acts as a general acid catalyst in the methylation reaction. This was experimentally supported by the pH-rate profile^18^. Computer simulations confirmed the reaction occurs in two steps (Fig. 1c)^21,22^. First, the proton is passed from the protonated C12 to m^6^G-N1 atom (ΔG^ǂ^ = 7.24 kcal/mol), followed by the methyl transfer from m^6^G-O6 to N1 of the target adenosine residue (A7) in the rate-limiting step (ΔG^ǂ^ = 19.43 kcal/mol). Interestingly, mutational analysis demonstrated that ribose modifications of C12 and U42 decisively affect the reaction rate (Fig. 1c)^19^. Most prominently, simultaneously replacing both C12 and U42 with their 2’-*O*-methylated counterparts (Cm12 and Um42, respectively, subsequently named MTR1m_2_) increased the experimentally observed reaction rate 15-fold^19^. A mechanistic explanation for the synergistic effect of two 2’-*O*-methyl (2’-OMe) groups resulting in the pronounced rate acceleration has not been provided previously.

This observation is intriguing given that natural RNA modifications, including 2’-OMe groups, were demonstrated to decisively impact gene expression by modulating RNA folding, assembly, stability and intermolecular association^23–25^. However, whether and how RNA modifications fine-tune the catalytic properties of naturally-occurring ribozymes has barely been investigated. While modification of active site nucleotides in artificial DNAzymes was reported to be beneficial for catalysis^26^, modifying active site residues in ribozyme systems, such as the hairpin and hammerhead ribozymes, resulted in deleterious effects^27,28^. In fact, MTR1 is the first example of a ribozyme, where installing a naturally occurring RNA modification in the active site enhanced catalysis^19^. Therefore, MTR1 represents a particularly suitable model system for exploring how RNA modifications can directly boost ribozyme catalysis.

Here, we employ classical and hybrid quantum-classical (QM/MM) molecular dynamics (MD) simulations and metadynamics to uncover how ribose ring methylations cooperatively enhance MTR1 catalysis. Our simulations demonstrate that the van der Waals interaction between the 2’-OMe groups of Cm12 and Um42 ribose rings, locked in the C3’ endo conformation, rigidifies the active site, reducing the rate-limiting activation free energy barrier. This is supported by biochemical experiments and in-line probing data that revealed tighter cofactor binding by MTR1m_2_. In parallel, we solved the crystal structure of MTR1m_2_ in the product state at 2.6 Å resolution. Importantly, the structure confirms the van der Waals interaction between Cm12 and Um42, validating our computer simulations.

## Results

### m^6^G binding in the MTR1 active site is unaffected by 2’-*O*-methylation

Computer simulations were initiated from the crystal structure of the trimolecular MTR1 construct (Fig. 1a, PDB code: 7Q7X)^19^. The structure captured MTR1 in the product state, i.e., the target A7 residue in R1 was in its methylated form m^1^A, while the cofactor was bound in the active site as demethylated guanine. To reconstitute the reactant state, the bond between the methyl group and the A7-N1 atom was broken and the methyl group was instead linked to the O6 atom of guanine to form m^6^G (Fig. 1b).

First, the conformational behaviour of the original non-modified MTR1 system (MTR1wt) and the improved MTR1 ribozyme containing 2’-*O*-methylated C12 and U42 (MTR1m_2_) was compared with classical MD simulations employing the χ_OL3_ force field (see Methods)^29,30^. Both systems were simulated in three 1–1.5 μs long replicas. MTR1wt and MTR1m_2_ remained well-folded during the simulations. The average backbone root mean square deviation (RMSD) from the crystal structure was 2.8 ± 1.1 Å and 3.5 ± 2.3 Å for MTR1wt and MTR1m_2_, respectively (Fig. S1). Root mean square fluctuation (RMSF) for each nucleotide identified the terminal residues as most mobile (Fig. S2). For active site nucleotides, we observed no significant RMSF difference between the systems (Fig. S2).

**Fig. 2:**
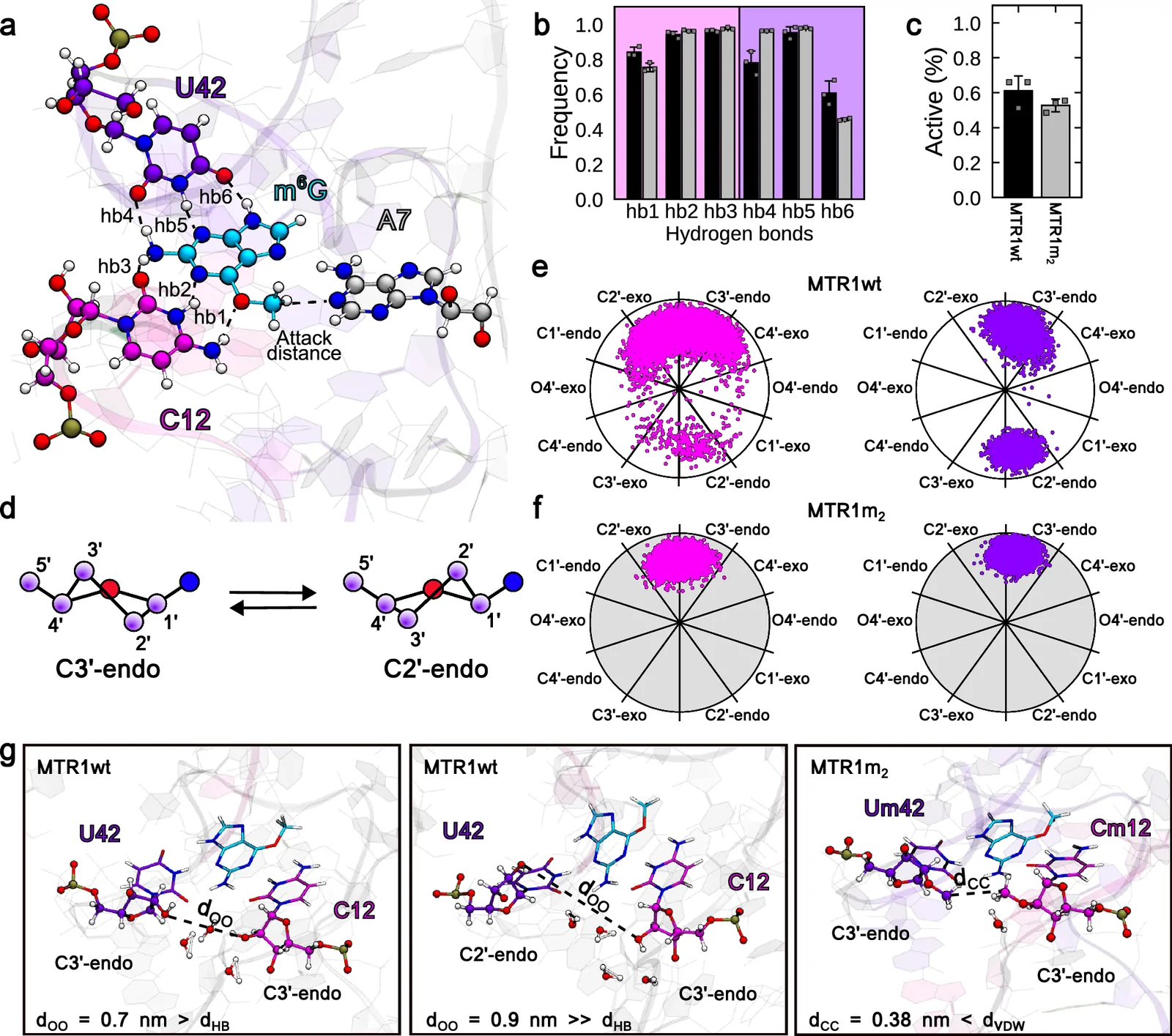
Conformational dynamics of the C12 and U42 ribose rings depend on the functional group present at the 2’ position. **a** Distances in the active site monitored during classical molecular dynamics (MD) simulations. **b** Comparison of average hydrogen bonding frequency between C12, U42 and m^6^G for the non-modified MTR1wt (OH at 2’ position of both C12 and U42; black bars) and modified MTR1m_2_ system (OMe at 2’ position of both Cm12 and Um42; grey bars). Hydrogen bond labels are as depicted in panel (**a**). **c** Percentage of active site configurations with reactive moieties in position for the methylation reaction. Black bars depict results of three replica simulations of MTR1wt, while grey bars correspond to results obtained in simulations of MTR1m_2_. **d** Scheme of the C3’ endo and C2’ endo conformations of the ribofuranose ring. **e**,** f** Pseudorotational wheel, depicting the frequency of different ribose conformations observed for C12 (pink dots) and U42 (violet dots) during MD simulations of the non-modified MTR1wt and modified MTR1m_2_ ribozyme. **g** Snapshots from MD simulation trajectories of MTR1wt and MTR1m_2_ depicting representative ribose conformations observed for C12/Cm12 and U42/Um42. Error bars denote  ± s.d. of the mean for *n* = 3 simulation replicas. Individual data points are shown as gray symbols. Source data are provided as a Source Data file.

Next, we analysed the persistence of hydrogen bonds responsible for m^6^G binding in the active site. Both protonated C12 (hb1-3 in Fig. 2a) and U42 (hb4-6 in Fig. 2a) formed three hydrogen bonds each to m^6^G. All displayed significant stability (> 75 %) along the MD trajectory in both systems, except hb6 (Fig. 2b). This hydrogen bond, formed between U42-O4 and m^6^G-N9 atoms, was more labile, present in 50–60% of simulated timeframes in both systems (Fig. 2b). Additionally, we estimated how often the catalytic site assumed a reactive configuration with all reactive moieties primed for methylation (Fig. S3). The prerequisite for this reaction to occur is sufficient proximity of A7-N1 and m^6^G-CM1 atoms (<4 Å) and that the forming and breaking bond are at an ~180^o^ angle (>150^o^). Applying these considerations, the probability for a reactive active site configuration was estimated at 61 ± 8% and 53 ± 4% for MTR1wt and MTR1m_2_, respectively (Fig. 2c). This suggests that in both systems m^6^G and A7 are positioned for the reaction comparably well.

### 2’-*O*-methylation of active site ribose rings enforces C3’ endo conformation

Conversely, the conformational behaviour of C12 and U42 ribose rings exhibited distinct differences between the two systems. Ribose rings are not planar, but assume one of ten different puckering conformations, defined with the pseudorotational angle^31,32^. Sugar puckering importantly impacts backbone geometry preferentially stabilizing the A-form or B-form RNA helix^31,32^. In RNA, there are two main ribose ring conformations; C3’ endo and C2’ endo conformation, also called North and South, respectively. In the C3’ endo conformation, the C3’ atom is positioned above the sugar plane, while in the C2’ endo conformation the C2’ atom is located above instead (Fig. 2d). However, sugar puckering is a dynamic phenomenon with interconversion between states occurring on ps to μs timescales^31,32^. Indeed, we observed a dynamic exchange between the C3’ endo and C2’ endo conformation for U42 in MTR1wt (Fig. 2e, Fig. S4). C12 also visited the C2’ endo conformation, however, only in one out of three simulation replicas. Instead, in MTR1m_2_, Cm12 and Um42 ribose rings maintained the C3’ endo conformation observed in the crystal structure, with the C2’ endo state never visited (Fig. 2f, Fig. S5). While 2’-*O*-methylation is known to stabilize the C3’ endo state in isolated nucleosides, such a pronounced shift in sugar puckering was unexpected^32–35^. We hypothesized that the observed behaviour was likely due to favourable local interactions. Indeed, the average separation between 2’-OMe carbon atoms across the simulated replicas was 3.8 ± 0.3 Å, below the sum of van der Waals radii for two methyl groups (4 Å). This suggests that there is an attractive interaction between the proximal 2’-OMe groups that additionally fortifies Cm12 and Um42 in the C3’ endo conformation. In MTR1wt, a comparable stabilizing interaction between the C12 and U42 2’-OH groups is absent, since these are too distant to hydrogen bond. In the MTR1wt crystal structure, C12-2’O and U42-2’O atoms are 7 Å apart, while the hydrogen bonding threshold between a hydrogen bond donor and acceptor is ~3.5 Å. Moreover, during MD simulations they were further separated due to C3’ endo to C2’ endo state transition of U42 (Figs. S6 and S7). This opened the catalytic site to incoming water molecules that hydrogen bonded to one of the hydroxyl groups or the m^6^G amino group (Fig. S8). This shifting also affected the interaction of U42 with m^6^G. Specifically, the C2’ endo conformation destabilized the hydrogen bond between U42-O2 and m^6^G amino group (hb4) and stabilized the bond between U42-O4 and m^6^G-N9 atoms (hb6) (Figs. S9 and 10).

Despite continuous and significant efforts to improve RNA force fields^36,37^, these current models still struggle to faithfully describe sugar puckering^35^. Thus, we sought to confirm the distinct preference of Um42 for the C3’ endo conformation in comparison to non-modified nucleotide with QM/MM MD^38^. Here, the active site, spanning C12/Cm12, U12/Um12, m^6^G and A7 (Fig. S11), was treated with the more accurate but computationally demanding Density Functional Theory, while other parts of the system were still described at classical force field level. First, few ps-long unbiased QM/MM MD simulations were performed to equilibrate the systems. Both the global ribozyme structure and the catalytic site were stable during these QM/MM MD simulations. (Figs. S12–14). To achieve appropriate sampling of sugar puckering dynamics and accurately determine the energetic profile of the transition between the C3’ and C2’ endo conformation of U42 and Um42, in MTR1wt and MTR1m_2_, respectively, QM/MM MD simulations were coupled with well-tempered metadynamics. As the collective variable (CV), we used the pseudorotational angle θ of U42 or Um42. The obtained sugar puckering free energy profiles confirmed the puckering preferences observed in classical MD simulations (Figs. S15 and S16, see Supplementary Discussion).

### Active site rigidification reduces activation barrier of the rate-limiting step

**Fig. 3:**
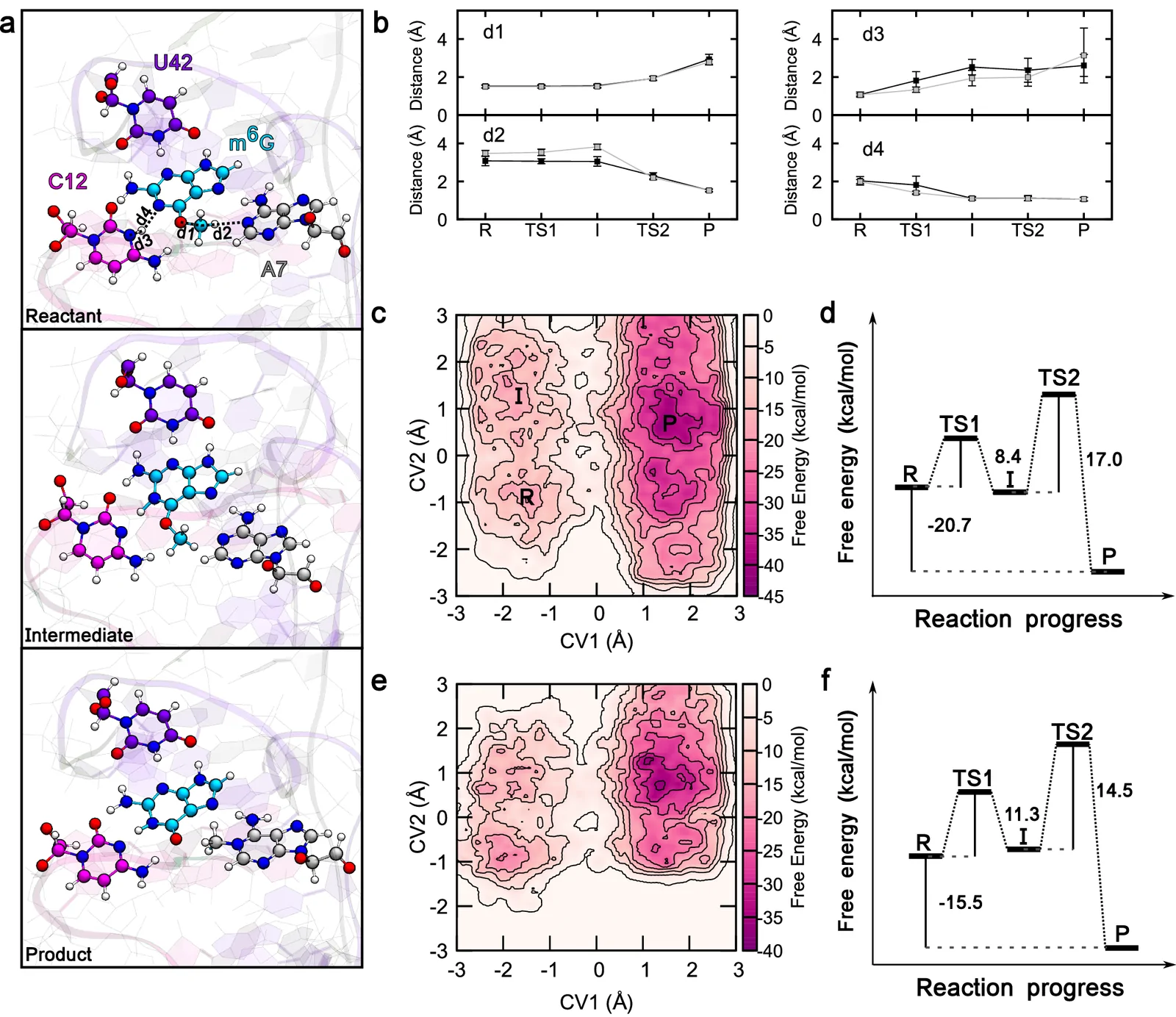
Molecular mechanism of the MTR1-catalyzed methylation reaction as observed from QM/MM metadynamics. **a** Snapshots of the active site in the reactant, intermediate and product state. Distances used for the definition of collective variables (d1–d4) are depicted in the reactant panel. **b** Average value and standard deviation of distances d1–d4 for different stages of the reaction (R, reactant state; TS1, first transition state; I, intermediate state; TS2, second transition state; P, product state). Black and grey dots correspond to distances observed in simulations of the non-modified MTR1wt and modified MTR1m_2_ system, respectively. For MTR1wt, statistics were derived from *n* = 4678, 1233, 4283, 1584 and 17677 simulation frames for the R, TS1, I, TS2 and P state, respectively. For MTR1m_2_, statistics were derived from *n* = 5373, 2788, 5644, 467 and 13170 simulation frames for the R, TS1, I, TS2 and P state, respectively. **c** Free energy surface for the methylation reaction in the MTR1wt system. Collective variable 1 (CV1) is the difference between the d1 and d2 distances, while collective variable 2 (CV2) corresponds to the difference of d3 and d4 distances. **d** Free energy profile as a function of the reaction progress in MTR1wt. Second reaction step (i.e. methyl group transfer) is the rate-limiting step. **e** Free energy surface for the methylation reaction catalysed by MTR1m_2_. Collective variables (CV1 and CV2) are as in panel (**c**). **f** Free energy profile as a function of the reaction progress for MTR1m_2_. Activation barrier of the rate-limiting step is lower than in the MTR1wt system. Source data are provided as a Source Data file.

To investigate the effect of disparate sugar puckering on catalysis, we simulated the methylation reaction in both MTR1 systems^39^. QM/MM MD simulations were coupled with metadynamics, employing two CVs. CV1 described the methyl group transfer and CV2 the translocation of the proton from C12-N3 to m^6^G-N1. Both CVs were defined as the difference between the breaking and forming bond length (CV1 = d1 – d2 and CV2 = d3 - d4 in Fig. 3a), thus increasing from negative to positive values as the reaction advanced from the reactant (R) to the product (P) state (Fig. 3b). Mirroring conformational preferences observed in QM/MM MD, C12 and U42 were initiated in the C3’ endo and C2’ endo conformation, respectively, in MTR1wt, while in the MTR1m_2_ system Cm12 and Um42 adopted the C3’ endo conformation. The minimum energy path connecting the reactants and products was determined from the two-dimensional free energy surface with the MEPSAnd programme (Fig. S17)^40^. In MTR1wt, the reaction proceeded as reported previously (Fig. 3a–d)^21,22^. First, the proton passed from the protonated C12 to m^6^G, yielding a stable intermediate (I). The activation barrier (ΔF^ǂ^) for this step was 8.4 ± 0.3 kcal/mol. This value may be slightly underestimated, since we observe that in the reactant state C12 can undergo a lactam-lactim isomerisation (Fig. S18). The lactim state, where the proton is on the C12-O2 atom, is characterized by CV2 values close to 0, although the first transition state (TS1) has not yet been reached. CV2 therefore cannot perfectly distinguish between R and TS1. However, ΔF^ǂ^ for reaching TS1 matches well the previously observed value (ΔG^ǂ^ = 7.24 kcal/mol)^21,22^, suggesting a minimal contribution of the rarely visited lactim state. Conversely, TS1 and I are well differentiated and therefore the relative free energy of the I state is accurately determined. After m^6^G protonation, the methyl hand-off from m^6^G-O6 to A7-N1 was observed with a ΔF^ǂ^ of 17.0 ± 0.7 kcal/mol, identifying this as the rate-limiting step, as reported previously^21,22^. The corresponding transition state (TS2) was observed at negative values of CV1 meaning the breaking bond was still shorter than the forming bond, in line with an associative mechanism. The overall reaction free energy, ΔF, was −20.7 ± 0.3 kcal/mol, suggesting a thermodynamically favourable product state. The MTR1m_2_ system followed an identical reaction pathway (Fig. 3b, e, f). However, the rate-limiting activation barrier was significantly lower at 14.5 ± 0.4 kcal/mol, in line with experimentally observed reaction acceleration. No lactam-lactim isomerisation was observed.

**Fig. 4:**
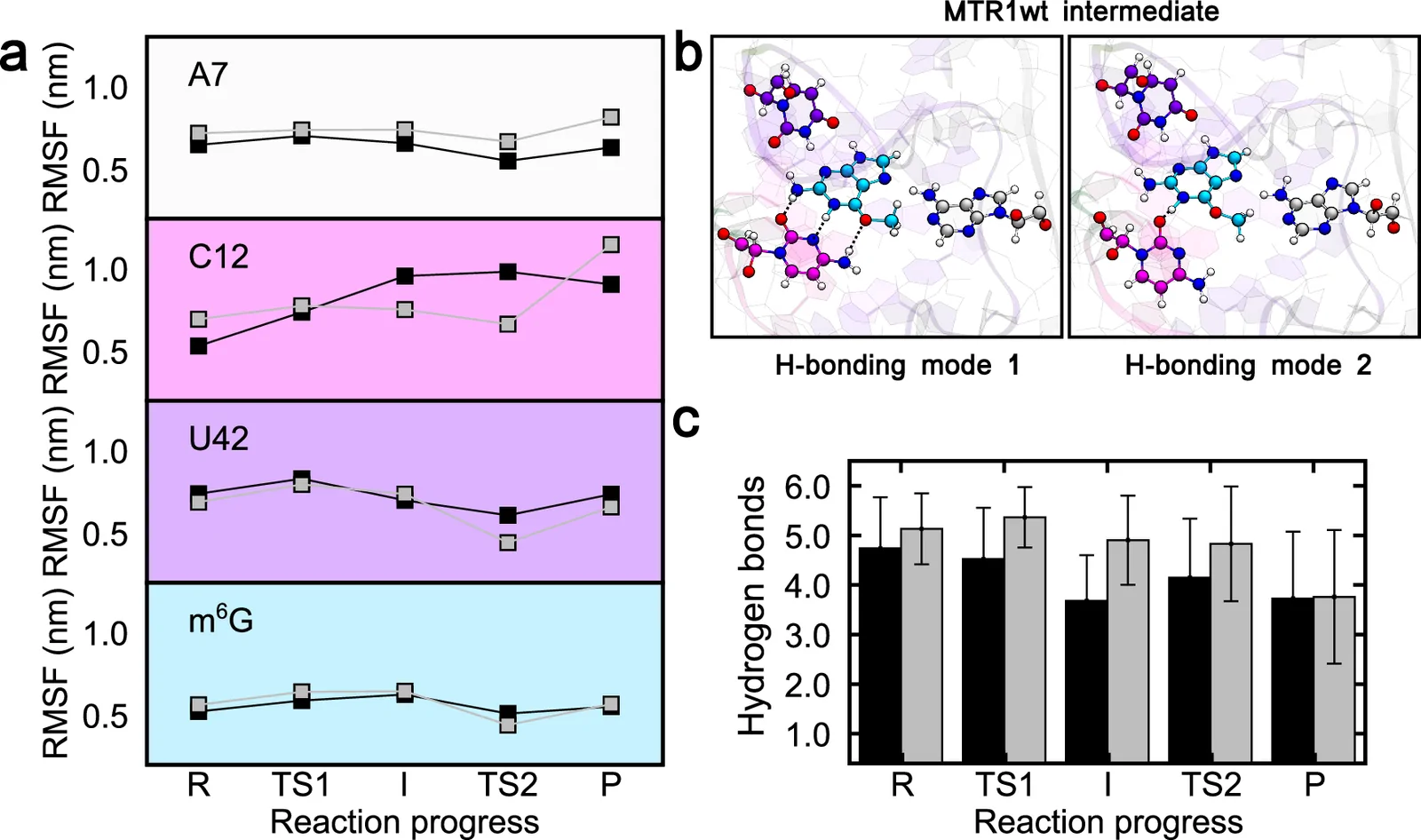
Active site of the non-modified MTR1wt system exhibits larger fluctuations during the reaction process. **a** Relative mean square fluctuations (RMSF) of the active site residues and the m^6^G cofactor during the reaction process (R, reactant state; TS1, first transition state; I, intermediate state; TS2, second transition state; P, product state). Black symbols correspond to the non-modified MTR1wt system, while grey symbols depict results obtained for the modified MTR1m_2_ system. C12 exhibits significantly larger positional fluctuations in MTR1wt than in MTR1m_2_. **b** Hydrogen bonding in the MTR1wt active site in the intermediate state. The proton passed from C12 to m^6^G can form a hydrogen bond with the C12-N1 atom or with the carbonyl group of C12. In MTR1m_2_, only the first hydrogen bonding mode is observed. **c** Average number of hydrogen bonds between C12, U42 and m^6^G (hb1-6) for different reaction stages. The average number of hydrogen bonds is larger in the modified MTR1m_2_ system (grey bars) than in the non-modified MTR1wt system (black bars), particularly in the intermediate state (I) and both transition states (TS1 and TS2). Error bars denote ± s.d of the mean. For MTR1wt, statistics were derived from *n* = 4678, 1233, 4283, 1584 and 17677 simulation frames for the R, TS1, I, TS2 and P state, respectively. For MTR1m_2_, statistics were derived from *n* = 5373, 2788, 5644, 467, and 13170 simulation frames for the R, TS1, I, TS2 and P state, respectively. Source data are provided as a Source Data file.

To explain the differences in the reaction free energy profiles, we analysed the flexibility of active site nucleotides and the guanine cofactor during the reaction and how it impacts key interatomic distances. In the reactant state, the MTR1wt and MTR1m_2_ active sites displayed comparable positional fluctuations (Fig. 4a). However, in the I and TS2 state, C12 was significantly more flexible in the MTR1wt system. This appears to arise due to the shifting of the C12 residue between two hydrogen binding modes (Fig. 4b). Specifically, the proton once passed from C12 to m^6^G could either re-establish a hydrogen bond with the C12-N1 atom or interact with the C12 carbonyl group. Consequently, other hydrogen bonds between C12 and the guanine cofactor were disrupted (Fig. 4c, Fig. S19). On average, we observed 1.6 hydrogen bonds between C12 and the guanine cofactor in the I state and 2.0 in TS2, in comparison to 2.3 and 2.2 hydrogen bonds observed in MTR1m_2_ for the I and TS2 state, respectively (Fig. S19). Additionally, in MTR1wt the average length of hb1, important for stabilizing the negative charge created on m^6^G-O6 upon methyl group departure, was 3.5 ± 0.9 Å and 3.0 ± 1.0 Å in the I and TS2 state, respectively. In MTR1m_2_, average hb1 length was 2.3 ± 0.6 Å in the I state and 2.5 ± 0.8 Å in TS2. This suggests MTR1m_2_ better stabilizes the guanine leaving group, consistent with the higher affinity of guanine measured by in-line probing (see below). Furthermore, decreased hydrogen bonding in MTR1wt resulted in less correlated movement of active site nucleobases in MTR1wt with respect to MTR1m_2_ (Fig. S20). Together, these results suggest that the attractive van der Waals interaction between the 2’-OMe groups of Cm12 and Um42 confers additional rigidity to the active site core that assures favourable positioning of catalytic residues during methylation and reduces the rate-limiting activation barrier.

### Crystal structure of MTR1m_2_ and biochemical comparison

**Fig. 5:**
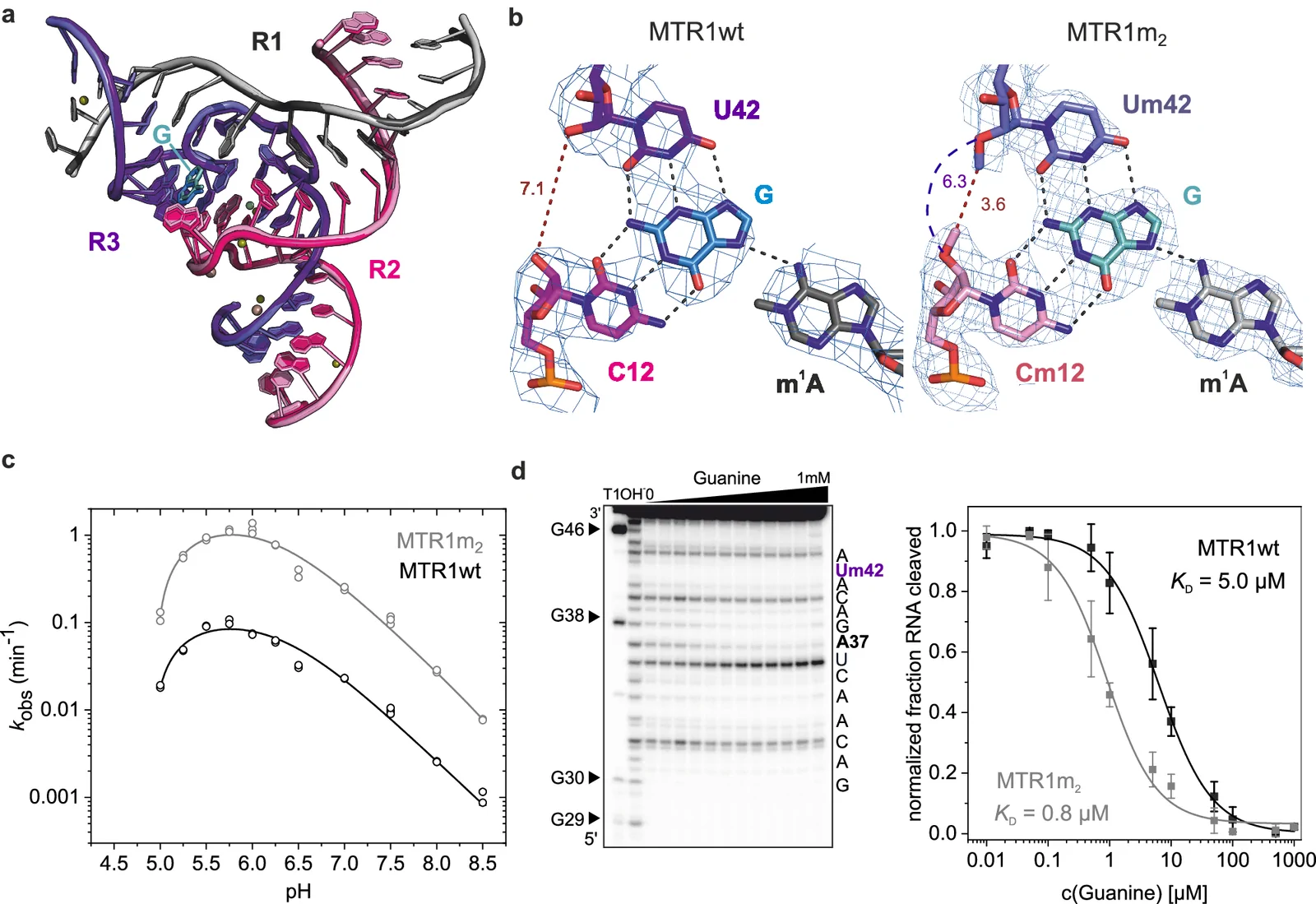
Structural and biochemical comparison of MTR1wt and MTR1m_2_. **a** Alignment of MTR1wt and MTR1m_2_ (PDB codes 7Q7X and 9S1D, respectively). Darker colours represent MTR1wt and the lighter version corresponds to MTR1m_2_, with R1 in grey, R2 in pink, R3 in violet and the cofactor in blue. **b** Active sites of MTR1wt and MTR1m_2_ embedded in the σA-weighted 2Fo − Fc density map contoured at 1σ. Colour code follows as in (**a**). Black dashed lines denote hydrogen bond interactions. **c** Comparison of the pH rate profile of MTR1wt^19^ and MTR1m_2_ shows accelerated reaction kinetics for MTR1m_2_ at all pH values measured, but similar pH rate profile for both. Individual data points are shown as empty symbols for *n* = 2 or 3 independent replicates. **d** Representative PAGE image of in-line probing gel of split MTR1m_2_ (pH 8.0, 20 mM MgCl_2_, 20 °C, 36 h; m^1^A-containing RNA-ribozyme complex, 0.01 – 1,000 µM guanine) together with analysis of normalized band intensities of A37 fit to a one-site-binding model for determination of apparent *K*_D_ value. See Fig. S25 for full gel image and analysis of U36 and G38. Error bars denote ± s.d. of the mean for *n* = 3 independent replicates. Source data are provided as a Source Data file.

To experimentally support the computational results, we performed structure probing in solution and solved the crystal structure of MTR1m_2_ (PDB code: 9S1D). Crystal constructs were prepared and grown by hanging-drop vapour diffusion similarly as described previously^19^. After diffraction experiments, the MTR1m_2_ structure was resolved at 2.6 Å resolution (Fig. S21, see “Methods”). The resultant MTR1m_2_ structure highly resembled the wild-type version (RMSD = 0.43 Å for 1,193 atom pairs) and the guanine was bound in the same manner in both structures (Fig. 5a, b). A notable feature of the MTR1m_2_ active centre is the two 2’-OMe groups pointing towards each other at a distance of 3.6 Å, situated at the regime of short-range van der Waals interactions^41^. Additionally, compared to the polar hydroxyl groups in MTR1wt, the clustering of two weakly polar 2’-OMe groups in MTR1m_2_ minimizes water contacts, which further bolsters their interaction via the hydrophobic effect. Interestingly, a similar distance and van der Waals interaction between RNA 2’-OMe groups has been observed in the cryoEM structure of the human 18S ribosomal RNAs, recently reported at 1.9 Å resolution (Fig. S22)^42^.

Given the similar architecture of the reaction centres in both structures, MTR1m_2_ likely exploits the same reaction mechanism as MTR1wt involving Cm12 protonation, as observed in the above described computer simulations. Indeed, kinetic assays of MTR1m_2_ at different pH conditions revealed a pH-activity profile similar to that of MTR1wt, with an optimal pH near 6.0, but with on average ~10-fold faster *k*_obs_ at 25 °C (Fig. 5c). We also compared temperature-dependent kinetics for both ribozymes between 0  and 30 °C. Increasing the temperature from 15  to 30 °C resulted in slower *k*_obs_, with a stronger decrease for MTR1wt (~5-fold) than for MTR1m_2_ (~3-fold). Below 15 °C, *k*_obs_ for both ribozymes changed less than twofold, with as slightly steeper slope observed in the Eyring plot for MTR1m_2_ (Fig. S23, Tables S3 and S4, see Supplementary Discussion). Furthermore, the methyl transfer by MTR1m_2_ was accelerated when the target adenosine was replaced by 7-deazadenosine (c^7^A) (Fig. S24), consistent with a recent report on non-modified MTR1^22^. The combination of c^7^A with Cm12 and Um42 resulted in the fastest RNA-catalysed methyl transfer reported so far with a *k*_obs_ of >1.5 min^–1^ at pH 6. The accelerating effect of the synergistic 2’-OMe groups was maintained even for less reactive substrate RNAs, such as 3-deazadenosine (c^3^A), which showed overall a slower methyl transfer (*k*_obs_ at pH 6.0 was 0.02 min^–1^ with MTR1wt, 0.09 min^–1^ with MTR1m_2_).

Next, we used in-line probing to study the effect of 2’-OMe groups on cofactor binding. Mimicking the post-catalytic state of MTR1wt or MTR1m_2_, 5’-^32^P-labelled R3 was hybridized to m^1^A-R1 and R2, and incubated with increasing concentrations of guanine at pH 8.0 and 20 mM Mg^2+^. The resulting cleavage patterns of MTR1m_2_ were similar to that of MTR1wt, showing an increasing intensity for the cleavage bands of U36 and decreasing intensity for A37 and G38 upon titration with guanine (Fig. 5d and Fig. S25). This is in line with previous results obtained for the full-length MTR1 ribozyme^19^. Analysis of the band intensities of U36, A37 and G38 resulted in apparent *K*_D_ values of 5.0 µM and 0.8 µM for MTR1wt and MTR1m_2_, respectively. Although obtained for the post-catalytic state, the more than fivefold lower apparent *K*_D_ of MTR1m_2_ suggests 2’-*O*-methylation causes a tighter cofactor binding site, consistent with the computational results described above.

## Discussion

Ribofuranose ring modifications impact RNA conformational dynamics, altering the local and potentially also global RNA structure^31,32^. This consequently affects recognition, ligand binding and susceptibility of RNA molecules to degradation by nucleases. For example, 2’-*O*-alkylation was shown to enhance stability of small RNAs^33,43^, such as miRNA and siRNA, stall translation elongation^44^ and gate ribosome and spliceosome assembly^45^. On the other hand, whether 2’-*O*-alkylation or other naturally occurring RNA modifications modulate catalytic properties of ribozymes is less clear. Mutational analysis of natural ribozymes, such as the hammerhead and hairpin ribozyme^27,28^, showed 2’-modifications of ribose rings can boost ribozyme activity indirectly by increasing substrate binding affinity. Conversely, including 2’-modifications in the active site mainly reduced catalytic activity by distorting active site geometry^27,28^. For example, Holliger and co-workers recently reported a 2’-OMezyme, an RNA-cleaving ribozyme consisting entirely of 2’-OMe nucleotides, that exhibited modest catalytic activity^46^. However, other synthetic DNAzymes (XNAzymes) containing various modified nucleotides have been developed for enhanced cellular activity and stability^26,47^. Our studies of MTR1 importantly demonstrate that strategically positioned 2’-OMe RNA modifications in the active site can improve ribozyme catalysis and facilitate turnover (Fig. S24). This was recently further demonstrated for RNA-catalysed RNA labelling using SNAPR, i.e. MTR1m_2_ in conjunction with *O*^6^-benzylguanines known as SNAP-tag substrates^48^.

**Fig. 6:**
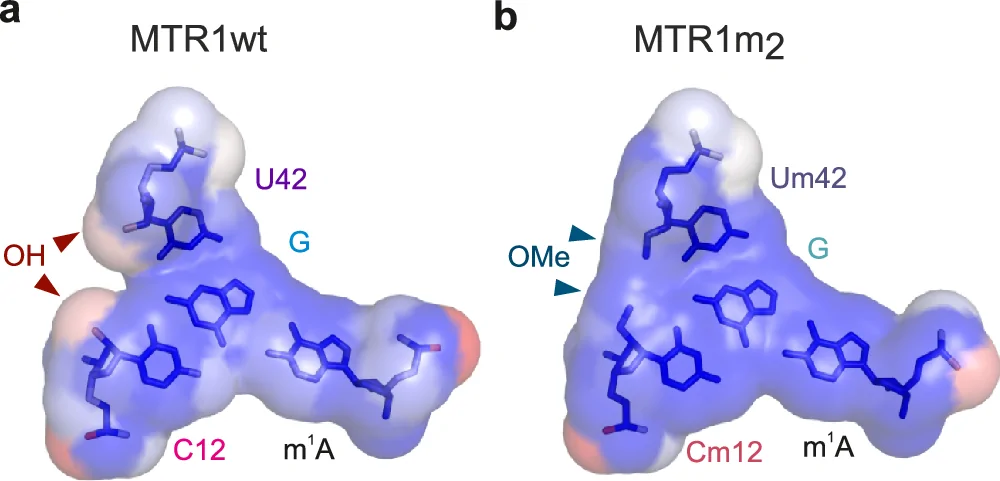
Solvent accessible surface area (SASA) of the ribozyme active sites. SASA shown for MTR1wt (**a**) and MTR1m_2_ (**b**) crystal structures. The blue-white-red spectrum represents the low to high solvent accessibility. Red and blue arrows indicate hydroxy groups and methoxy groups, respectively. Figure prepared in PyMOL v3.3.1 with solvent radius of 1.4 Å.

By performing multiscale MD simulations and solving the MTR1m_2_ crystal structure, we uncovered what drives the observed rate accelerations following concomitant 2’-*O*-methylation of C12 and U42 in the MTR1 active site. Specifically, the van der Waals interaction between the proximal 2’-OMe groups locked the Cm12 and Um42 ribose rings in the C3’ endo conformation and shielded the active site from incoming water molecules. This hydrophobic effect can be visualized in the solvent accessible surface area (SASA) of the active sites in both crystal structures (Fig. 6). Due to its polar nature, the 2’-OH groups in MTR1wt present a higher SASA than the 2’-OMe in MTR1m_2_. The additional hydrophobic interaction between the two 2’-OMe groups acts as a nonpolar barrier, lowering the accessibility of water into the reaction centre. This echoes the observation of reduced water contacts with m^6^G in the MTR1m_2_ system during classical MD simulations (Fig. S8). Moreover, the anchoring of the ribose rings further extended to the core of the active site, reflected in more persistent hydrogen bonding between Cm12, Um42 and m^6^G during the reaction process in comparison to the non-modified MTR1wt, particularly in the intermediate and second transition state. This reduced the rate-limiting activation barrier for 2.5 ± 0.8 kcal/mol. The experimentally observed increase in the reaction rate upon introduction of Cm12 and Um42 into MTR1 matches an activation barrier reduction of ~1.6 kcal/mol^19^, displaying good accordance with the computed value. We posit the minor difference between the theoretical and experimental value may be due to the conformational heterogeneity of the MTR1wt active site in solution, with U42 able of assuming both C3’ endo and C2’ endo state (see Supplementary Discussion).

That Cm12 and Um42 are locked in the C3’ endo conformation due to the van der Waals interaction between the 2’-OMe groups and not merely because of the influence of 2’-modification on sugar puckering is supported by several lines of evidence. First, the free energy difference between the C3’ and C2’ endo state of Um42 observed in our QM/MM MD simulations was significantly more pronounced than previously observed for isolated Um^33,34^. Second, in the MTR1 system containing a single 2’-OMe nucleotide, Cm12 or Um42, the reaction rate increase is only twofold^19^. As MD simulations suggest U42 is the main source of ribose flexibility, MTR1(Um42) should have a similar catalytic efficiency as MTR1m_2_ if 2’-*O*-methlyation was sufficient for ribose rigidity. Third, the MTR1m_2_ crystal structure confirmed that the 2’-OMe groups are sufficiently close to interact. The higher affinity for the cofactor is consistent with reduced dynamics of the modified active site. Finally, replacing the 2’-OMe groups of Cm12 and Um42 by 2’-F atoms showed a much weaker accelerating effect^19^, although the electronegative fluorine atoms are known to stabilize the C3’endo ribose pucker^49^. However, due to the smaller size of the fluorine atoms compared to OMe groups, their distance is too far for direct interaction, and their polarity may rather enhance hydration than shield the active site from water molecules.

Taken together, our results decrypt in molecular detail how RNA modifications directly dictate the catalytic properties of RNA molecules. Due to the large number of different possible RNA modifications (>170)^8^, we believe the uncovered mechanism represents only one of many alternative regulatory modes still to be unveiled. Furthermore, our outcomes may contribute to establishing general principles for designing more efficient ribozymes^50–53^. Ribozymes able of installing naturally occurring RNA modifications, such as MTR1, carry potential as novel therapeutic agents in various human diseases driven by RNA modification pattern changes, including cancer and different neurological disorders^25^. However, current artificial ribozymes are weak catalysts (*k*_cat_ < 2 min^–1^)^9–14^. Increasing their turnover rate will be crucial for developing ribozymes with any applicative potential. The crystal structure of MTR1m_2_ demonstrates that even small perturbations of the active site can lead to a significant increase in the catalytic rate. Subtly modifying the active site is a well-established approach for engineering more efficient protein enzymes^54,55^ and may prove viable also for ribozymes. As the 3-helix junction structure is a common ribozyme scaffold, inclusion of a van der Waals interaction between 2’-OMe groups could prove a general strategy for increasing catalytic efficiency. More broadly, our results suggest that increasing the rigidity of ribozymes’ active sites, thus making them more protein-like, may be necessary for improving their catalytic properties. The good agreement between the MTR1m_2_ active site as observed in QM/MM MD simulations and crystal structure, suggests QM/MM MD simulations may be a crucial tool for ribozyme design-oriented studies. Finally, the discovery that RNA modifications directly influence catalysis supports the idea that modified ribonucleotides were central for the functioning of primordial ribozymes that powered early evolution according to the RNA world hypothesis^6,7^.

## Methods

### Classical molecular dynamics

Simulated systems were prepared from the MTR1 crystal structure (PDB code: 7Q7X)^19^. Before starting molecular dynamics (MD) simulations, the overhangs present in the MTR1 crystal structure were removed. Simulation box and topology was built with the tleap tool from the AmberTools22 package^56^. The ribozyme was placed in a rectangular simulation box, such that the minimum distance between the ribozyme and box edge was at least 12 Å. For constructing the topology we employed the χ_OL3_ force fields, which gives best performance for the majority of RNA systems^29,30^. Modified nucleotides were parametrized in house. Merz–Kollman-restrained electrostatic potential (RESP) charges for *O*^6^-methylguanine (m^6^G) and protonated cytidine nucleobase were calculated with the Hartree Fock method and the 6-31 G* basis set in Gaussian09^57,58^. Parameter files were generated with antechamber programme from the AmberTools package using Amber atom types^56^. The charge distribution across the methoxy group of 2’-*O*-methylated uridine and cytidine was taken from the modrna08 forcefield^59^, however the total charge was restricted to match the 2’-hydroxyl group of non-modified residues to ensure compatibility with the χ_OL3_ force fields. Water molecules were described with the TIP3P water, while KCl ions at 150 mM concentration were modelled with Li-Merz monovalent ion parameters for TIP3P water model^60^.

MD simulations were run in Gromacs2022.3^61^. Newton’s equations of motion were solved with the leap-frog algorithm coupled with a 2 fs time step. Electrostatic interactions were calculated with the particle mesh Ewald method^62^. The cut off for van der Waals and electrostatic interactions was set at 1.2 nm. Bonds involving hydrogen atoms were constrained with the LINCS algorithm. Each system was first equilibrated in the canonical (NVT) ensemble to 300 K for 10 ns. Next, pressure equilibration was performed for 10 ns in the isothermal–isobaric (NPT) ensemble at 1 bar. Temperature and pressure were controlled with the V-rescale thermostat and the p-rescale barostat, respectively^63,64^. During equilibration, position restrains (1000 kJ/mol) were placed on backbone heavy atoms. Additionally, the so called H-bond fix was applied to the final two base pairs of each ribozyme arm, to prevent fraying of base pairs at the edges^65^. After equilibration, position restraints were removed and the MD trajectory was collected for 1–1.5 µs. For each system, simulations were performed in triplicates. Trajectory analysis was performed with the cpptraj programme from AmberTools and MDAnalysis python package^66,67^.

### Hybrid quantum-classical molecular dynamics simulations

Hybrid quantum-classical (QM/MM) MD simulations were performed in CP2K (version 2023.2)^68^. The QM zone was described with density functional theory utilizing the Becke–Lee–Yang–Parr (BLYP) functional and a dispersion correction^69–71^. Double-ζ molecularly optimized basis set was used along with Goedecker–Teter–Hutter pseudopotentials^72^. The plane wave cut off was positioned at 400 Ry. This set up proved appropriate for natural ribozymes^73^. The QM zone was composed of m^6^G, C12, U42 and A7 nucleotides, for a total of 114 atoms in the non-modified MTR1wt system and 120 atoms in the modified MTR1m_2_. Bonds to hydrogen atoms were restrained in the MM zone. QM/MM MD simulations were started from the final frame of a representative classical MD simulations replica. Initially, each system was relaxed in a microcanonical (NVE) ensemble for 250 steps. Next, 500 step long simulated annealing to 125 K was performed using the Langevin thermostat. The simulated system was then heated in a step-wise manner from 150  to 300 K using the NVT ensemble. Temperature was maintained with the V-rescale thermostat. Two thermostats, both with 10 fs time constant, were applied; one maintained temperature in the QM region and one in the rest of the system. After equilibration, the trajectory was collected over 5 ps. The time step was 0.5 fs. The thermostat time constant was increased to 1000 fs during production runs.

### QM/MM metadynamics simulations of sugar puckering

To sample the sugar puckering behaviour of U42 and Um42 in MTR1wt and MTR1m_2_, respectively, we performed QM/MM well-tempered metadynamics with CP2K coupled with the PLUMED plugin (version 2.9) that can perform a variety of enhanced-sampling methods^74,75^. As a collective variable (CV) we used the pseudorotational angle (*θ*) of U42 or Um42^35^. Gaussian hill height and width were 2.4 kJ/mol and 0.1, respectively. Bias factor, scaling Gaussian hill heights along the trajectory, was set at 20. Gaussian hill was positioned every 50 steps. Simulations were performed at 300 K. Metadynamics simulations were initiated from the final equilibrated frame of unbiased QM/MM MD simulations. Consequently, at the start of QM/MM metadynamics simulations of sugar puckering in MTR1wt, C12 and U42 were in the C3’ endo and C2’ endo state, respectively, while in MTR1m_2_, Cm12 and Um42 were both in the C3’ endo state. For MTR1wt, which exhibited a significant conformational plasticity of the active site, occurring at timescale inaccessible to QM/MM MD, we ran an additional metadynamics simulation starting from a pre-production MD structure, where C12 and U42 were both in the C3’ endo state. QM zone was defined as in unbiased QM/MM MD. To restrict the sampling of the conformational space to pseudorotational angle values of interest (0–180^o^), a repulsive wall was set at sin(*θ*) = –0.25. Free energy profile as a function of the pseudorotational angle was calculated with the sum_hill tool that is part of PLUMED. Error bars were calculated with block averaging.

### QM/MM metadynamics simulations of the methyltransfer reaction

To simulate the methyltransferase reaction, we performed QM/MM metadynamics with CP2K employing two collective variables (CVs). CV1 described the methyl group transfer and was defined as the difference of the distance between m^6^G-CM1 and m^6^G-O6 atoms and the distance between m^6^G-CM1 and A7-N1 atoms. On the other hand, CV2 was defined as the difference of the distance between C12-N3 and C12-H1 atoms and the distance between C12-H1 and m^6^G-N1 atoms. Gaussian hills with height of 0.004 Ha and width of 0.2 Bohr were deposited every 50 steps. For CV1, a repulsive wall was placed at -2.5 Å and 2.5 Å. QM zone was defined as in unbiased QM/MM MD. Metadynamics simulations were performed for a minimum of 75 ps per system. Two-dimensional free energy surface was reconstructed with graph_sopt programme of CP2K. The minimum energy path describing the reaction free energy profile was determined with the MEPSAnd programme^40^. Error bars were calculated with block averaging.

### General biochemical methods

All standard solvents and chemicals including urea, acrylamide/bisacrylamide stock solution, and buffer reagents were purchased from commercial suppliers and used without further purification. Water used for all experiments was purified with a Milli-Q-unit from Sartorius. RNase T1 and T4 PNK were purchased from ThermoFisher Scientific. γ-^32^P-ATP was from Hartmann Analytic GmbH. *O*^6^-methylguanine (m^6^G) was purchased from Sigma Aldrich. Vivaspin desalting columns were from Sartorius. RNA oligonucleotides were synthesized in house using solid phase synthesis with phosphoramidite chemistry (see below). 5’-*O*-DMT-2’-*O*-TOM-protected and 5’-*O*-DMT-2’-*O*-methyl RNA phosphoramidites and 5’-*O*-DMT-protected CPG supports (1000 Å, 25-35 μmol/g) were purchased from ChemGenes or Sigma Aldrich. Phosphoramidites for incorporation of c^3^A and c^7^A were prepared following published procedures^14,76,77^.

Imaging of polyacrylamide gels containing radioactive oligonucleotides was achieved by exposure to phosphor storage screens followed by scanning with a Typhoon Phosphorimager from Cytiva. Analytical anion-exchange HPLC was performed on a GE Healthcare Life Sciences ÄKTA micro system. RNA solid phase synthesis was carried out on a K&A DNA/RNA synthesizer (H6/H-8).

### RNA synthesis

RNA oligonucleotides were prepared by solid-phase synthesis using standard 2’-*O*-TOM-protected or 2’-*O*-methyl ribonucleotide phosphoramidites. RNAs were deprotected using a 1:1 (v/v) mixture of 28 % aq. NH_3_ and 40 % aq. MeNH_2_ (known as AMA) at 37 °C for 6 h, followed by deprotection with 1 M tetrabutylammonium fluoride (TBAF) in THF at 25 °C for 16 h. The oligonucleotides were desalted by size exclusion chromatography followed by purification via denaturing polyacrylamide gel electrophoresis (PAGE), extraction and ethanol precipitation.

RNA purity and quality was confirmed by anion-exchange HPLC (Dionex DNAPac PA200, 2 × 250 mm at 60 °C; solvent A: 25 mM Tris-HCl (pH 8.0), 6 M urea; solvent B: 25 mM Tris-HCl (pH 8.0), 6 M urea, 0.5 M NaClO_4_; linear gradient: 0-40% solvent B, with a slope of 4% solvent B per column volume). The identity of the RNAs was confirmed by HR-ESI-MS (negative ion mode). A summary of the RNA sequences together with their respective calculated and measured masses is provided in Table S1. The HPLC chromatograms are summarized in Fig. S26.

### 5’-^32^P-RNA labelling

5’-^32^P-labelling of RNA R1 for kinetic assays and RNA R3 for in-line probing was performed in a total volume of 10 µL 1x PNK buffer (50 mM Tris, pH 7.6, 10 mM MgCl_2_, 5 mM DTT, 0.1 mM Spermidine) containing 200 pmol of RNA, 5 µCi γ-^32^P-ATP and 5 U T4 PNK. The reaction was incubated for 1 h at 37 °C, quenched by the addition of loading dye and purified on 15 % (for R3) or 20% denaturing PAGE (for R1) followed by extraction and EtOH-precipitation.

### Determination of MTR1m_2_ pH-dependence

The pH-dependence of MTR1m_2_ was analysed using single-turnover kinetic assays. In a total volume of 10 µL, 100 pmol of each ribozyme strand (R2 and R3) was mixed with 500 IPS 5’-^32^P-labelled target RNA R1 in 1x reaction buffer (120 mM KCl, 5 mM NaCl and 50 mM buffer, pH 4.5–8.5 (the following buffers were used: sodium acetate: pH 4.5, 5.0 and 5.5; Bis-Tris: pH 6.0 and 6.5; HEPES: pH 7.0, 7.5 and 8.0; Tris-HCl: pH 8.5)) together with 100 μM m^6^G and 40 mM MgCl_2._ An annealing step (3 min at 95 °C and 10 min at 25 °C) was performed prior to the addition of m^6^G and MgCl_2_ to ensure proper folding and hybridization of the ribozyme-target RNA complex. The reaction mixture was incubated at 25 °C and aliquots (1 µL) were taken at appropriate time points, quenched immediately by the addition of loading dye (4 µL) and resolved on 20% denaturing PAGE. Methylation yields were determined by the quantification of band intensities corresponding to reacted and unreacted RNA using phosphorimaging. The yield versus time data was then fit to the pseudo first-order kinetic equation *Y* = *Y*_max_(1 – e^-*k*obs*t^), in which *k*_obs_ is the observed reaction rate and *Y*_max_ is the final yield using Origin (2024). All kinetic assays were carried as two independent replicates.

### Temperature dependence of ribozyme kinetics and Eyring-analysis

Kinetic assays were performed and analyzed as described in the “Determination of MTR1m_2_ pH-dependence” section, but in the presence of 3 mM MgCl_2_ at pH 6.0. The temperature dependence of the ribozyme from 0 to 15 °C was subsequently analyzed using Eyring transition state theory. A plot of ln(*k*_obs_/T) vs. 1/*T* was fit to Eq. 1 to obtain the activation enthalpy (Δ*H*^ǂ^) and entropy (Δ*S*^ǂ^).

1 $${{\rm{In}}}\left(\frac{{k}_{{{\rm{obs}}}}}{T}\right)=\frac{-\Delta {H}^{{\ddagger} }}{R}\frac{1}{T}+{{\rm{In}}}\left(\frac{{k}_{{{\rm{B}}}}}{h}\right)+\frac{\Delta {S}^{{\ddagger} }}{R}$$

In Eq.( 1), T is the absolute temperature in Kelvin, *R* is the gas constant (8.314 J mol^–1^ K^-1^), *k*_B_ is the Boltzmann constant (1.38 × 10^–23 ^J K^–1^) and *h* is Planck’s constant (6.626 ×10^-34 ^J s).

### *K*_d_ determination via in-line probing

#### Preparative scale RNA modification

The m^1^A containing substrate RNA used for in-line probing experiments of the post-catalytic state was obtained via a preparative scale modification of R1 using MTR1m_2_. In a total volume of 20 µL, 1 nmol of substrate RNA R1 were mixed with 1.2 nmol of each ribozyme strand in 1x reaction buffer (120 mM KCl, 5 mM NaCl, 50 mM Bis-Tris, pH 6.0) along with 100 µM of m^6^G and 40 mM MgCl_2_. An annealing step (3 min at 95 °C, 10 min at 25 °C) was performed prior to the addition of m^6^G and 40 mM MgCl_2_. After overnight incubation at 25 °C, the reaction was precipitated with EtOH, purified using 20 % denaturing PAGE, extracted and the modified RNA was recovered by EtOH-precipitation.

#### In-line probing reaction

In-line probing was performed using the 5’-^32^P-labelled ribozyme strand R3 in complex with unlabelled m^1^A RNA R1 and the remaining ribozyme strand R2. In a total of 2.5 µL, 125 IPS 5’-^32^P-R3 were annealed to 25 pmol of R1 and R2 (3 min at 95 °C and 10 min at 25 °C). Following, 100 mM Tris-HCl (pH 8.0) and 100 mM KCl were added together with 20 mM MgCl_2_ and guanine in various concentrations to yield a final total volume of 5 µL. The samples were incubated at 20 °C for 36 h, quenched by the addition of loading dye (5 µL), resolved on 20% denaturing PAGE, and visualized using autoradiography. As references, an alkaline hydrolysis lane (250 IPS of 5′-^32^P-R3 incubated in 10 µL 20 mM NaOH for 4 min at 95 °C) and an RNase T1 digestion (150 IPS of 5′-^32^P-R3 in 10 µL 50 mM Tris (pH 7.5) digested with 1U RNase T1 at 37 °C for 45 s) were used. Gels were analysed by quantifying and then normalizing the band intensities for positions most sensitive to changes in guanine concentration (A36, A37, G38). The data was plotted against the guanine concentration and fitted to a one-site binding model using Origin (2024) to obtain the *K*_d_ values.

### Complex formation and crystallization

Crystallization complexes were formed by mixing the three RNA strands (R1, R2 (OMe), R3 (OMe)) in a 1:1:1 molar ratio (5 nmol each). The sample was desalted via ultrafiltration using a Vivaspin 500 column (3000 MWCO) and extensive washing with water followed by concentration to a total volume of 20 µL. 50 mM KCl and 10 mM HEPES (pH 7.5) were added and the sample was heated to 95 °C for 3 min and cooled at 20 °C for 15 min to ensure correct folding and hybridization of the ribozyme-substrate RNA complex. Then, 240 µM m^6^G was added along with 5 mM MgCl_2_ to a total volume of 25 µL and a final complex concentration of 200 µM. The construct was then stored at -20 °C until further use.

Crystals were grown at 20 °C using the hanging drop vapour-diffusion method with a 1 µL drop volume. The RNA construct was mixed in a 1:1 ratio with solutions containing 100 mM NaCl, 100 mM LiCl, 10 mM MgCl_2_, 50 mM MES (pH 6.4–6.7) and 36–42% MPD. Rod shaped crystals appeared after 3–4 days and were harvested after 7–8 days by flash freezing in liquid nitrogen without additional cryoprotection.

### Data collection and structure determination

X-ray diffraction experiments were conducted at 100 K at beamline P11 in German Electron Synchrotron (DESY). Diffraction data were collected at a wavelength of 1.033 Å on an Eiger2 X 16 M detector. Images were processed with XDS (2024.June.30)^78^, followed by scaling analysis in AIMLESS and calculation of MATTHEWS_COEF in CCP4 programme suite (version 9.0.004)^79^. Phase was solved by molecular replacement in Phaser^80^, using the native MTR1 structure (PDB: 7Q7X) as search model, which resulted in a single structure solution with a translation function Z-score of 33. Density modification was performed in RESOLVE^81^ with unmethylated MTR1 structure (7Q7X without N1-methylation at A7 in chain A) as phase input to resolve the continuous density along the base stacking (Fig. S21a). The improved phase was incorporated into refinements in Phenix (version 1.21.2_5419)^82^ and the three methyl groups (at N1 of A7, O2’ of C12 and O2’ of U42) were modelled according to the resulting difference map in COOT (version 0.9.8.95)^83^ (Fig. S21b, c). Restraints of the ligand (guanine) and the modified nucleotides (ligand ID: 1MA, OMC and OMU for m^1^A, Cm and Um respectively) were directly imported from the monomer library in COOT. Figures of the MTR1 structures were prepared in PyMol (Schrödinger, version 3.1.1). Data collection and refinement statistics are summarized in Table S2.

### Reporting summary

Further information on research design is available in the Nature Portfolio Reporting Summary linked to this article.

## Supplementary information

Supplementary Information

Reporting Summary

Transparent Peer Review file

## Source data

Source Data

## Data Availability

Raw diffraction images of the reported crystal structure have been deposited in the XRDa database under the accession code XRD-00546. The atomic coordinates and structure factors were deposited to the PDB (www.rcsb.org) under accession codes 9S1D. Existing structures used throughout the study were also obtained from the PDB under accession codes 7Q7X and 8QOI. Classical MD trajectories and input files required to replicate all our simulations were deposited to Zenodo as entry 18259069. Due to their size, QM/MM MD trajectories are promptly available from the corresponding author Alessandra Magistrato (e-mail: alessandra.magistrato@sissa.it) upon request. Source data are provided with this paper.
