# Supplementary material for: A magic methyl effect in the active site of a methyltransferase ribozyme
Source: Nat Commun. 2026 Jul 17;17:8784. doi: 10.1038/s41467-026-75575-8 (PMC13493877; doi:10.1038/s41467-026-75575-8)
Supplement: Supplementary file 1 [file 41467_2026_75575_MOESM1_ESM.pdf]

Supplementary information

A magic methyl effect in the active site of a methyltransferase ribozyme

Jana Aupič,[a,*] Hsuan-Ai Chen,[b] Carolin P.M. Scheitl,[b] Claudia Höbartner,[b,c,d*] Alessandra Magistrato[a,*]

[a]CNR - Istituto Officina dei Materiali (IOM) c/o Scuola Internazionale Superiore di Studi Avanzati (SISSA), Via Bonomea 265 - 34136 Trieste, Italy

[b]Julius-Maximilians-Universität Würzburg, Institute of Organic Chemistry, Am Hubland, 97074 Würzburg, Germany

[c]Julius-Maximilians-Universität Würzburg, Center for Nanosystems Chemistry, 97074 Würzburg, Germany

[d]Cluster for Nucleic Acid Sciences and Technologies – NUCLEATE

Corresponding authors:

Alessandra Magistrato: alessandra.magistrato@cnr.it

Claudia Höbartner: claudia.hoebartner@uni-wuerzburg.de

Jana Aupič: jana.aupic@sissa.it

This PDF file contains:

Supplementary Discussion

Supplementary Figures S1-S26

Supplementary Tables S1-S4

Supplementary References

Supplementary Discussion

QM/MM metadynamics simulations of sugar puckering

To confirm the sugar puckering preferences observed in classical MD, we ran QM/MM MD simulations coupled with well-tempered metadynamics, utilizing the pseudorotational angle θ of U42 or Um42 as the collective variable (CV) in the MTR1wt or MTR1m$_2$ system, respectively. QM/MM metadynamics simulations were initiated from the final frame of representative classical MD simulations replicas that were first relaxed by running 5 ps long unbiased QM/MM MD simulations. As such, metadynamics simulations were initiated with C12 and U42 in the C3' endo and C2' endo state, respectively, in the MTR1wt system, while at the start of the simulations for the MTR1m$_2$ system, Cm12 and Um42 were both in the C3' endo state. As the active site of MTR1wt showed significant flexibility, reflected in the distancing of C12 and U42 2'-OH groups and an influx of water molecules in the active site (Figs. S7 and S8), we ran an additional metadynamics simulations starting from a pre-production MD state, where C12 and U42 were both in the C3' endo state as observed in the crystal structure. The simulations demonstrated that in MTR1m$_2$ Um42 preferentially assumed the C3' endo state with the free energy difference between the C3' endo and C2' endo conformation ($\Delta F_{C3' \rightarrow C2'}$) of 19.3 ± 0.2 kcal/mol (Fig. S15). In the MTR1wt system initiated from the post-production MD state, the C3' endo state of U42 was less favourable than the C2' endo state with $\Delta F_{C3' \rightarrow C2'}$ of -17.2 ± 0.3 kcal/mol. Instead, if the MTR1wt system was initiated from the pre-production MD state, the difference was smaller ($\Delta F_{C3' \rightarrow C2'}$ of -6.1 ± 0.3 kcal/mol, Fig. S15). This suggests that the sugar puckering preferences arising from our simulations strongly depend on the surrounding structural context, with molecular interactions between the ribose rings and the surrounding molecular environment decisively influencing puckering behaviour, as previously observed for different RNA systems[1–3]. Indeed, free energy differences between the C3' and C2' endo state for single non-modified and 2'-*O*-methylated RNA nucleotides were reported to be less pronounced, below 10 kcal/mol[4–6]. In case of MTR1m$_2$, the C3' endo state appears to be primarily enforced by the van der Waals interaction between the 2'-OMe groups, while in the MTR1wt system the C2' endo conformation of U42 is stabilized by a persistent hydrogen bond between the U42 2'-OH group and A43-OP2 atom (Fig. S16).

Eyring Analysis

The temperature dependence of ribozyme activity was analyzed using Eyring transition state theory to obtain the activation enthalpy ($\Delta H^‡$) and entropy ($\Delta S^‡$) in the MTR1wt and MTR1m$_2$ system and thus identify the main driver for improved catalysis in MTR1m$_2$ (Table S3). The analysis resulted in $\Delta H^‡$ of 1.8 ± 0.7 kcal/mol and 5.0 ± 1.0 kcal/mol, and $\Delta S^‡$ of -67.8 ± 2.5 cal/molK and -50.9 ± 3.3 cal/molK for MTR1wt and MTR1m$_2$, respectively (Table S4). Considering experimental uncertainty and error propagation the data should not be overinterpreted. With this in mind, our data tentatively suggest that improved catalysis is due to entropic considerations. If the rate acceleration would be brought about only by an additional van der Waals interaction between the two 2'-OMe groups of Cm12 and Um42, the $\Delta H^‡$ should be smaller in MTRm$_2$ due to improved chemical stability. Instead, we observed a 3.2 kcal/mol higher $\Delta H^‡$ for MTR1m$_2$ in comparison to MTR1wt (error range of 1 kcal/mol). This unfavorable $\Delta H^‡$ in MTR1m$_2$ is offset by the entropy gain of 16.9 cal/molK (error range of 2.5-3.5 cal/molK). In our simulations, we observed no significant change in active site solvation along the methylation reaction for neither MTR1wt nor MTR1m$_2$, suggesting that the disparate

entropic cost is not primarily due to differences in water ordering. Instead, our simulations suggest that the more favourable $\Delta S^{‡}$ for $MTR1m_2$ arises from the more rigid, pre-organized active site that resembles the transition state (Fig. 5, Fig. S20).

Comparison of experimentally and computationally determined rate acceleration

The experimentally observed increase in the methylation reaction rate upon introduction of Cm12 and Um42 modifications into MTR1wt corresponds to a reduction in the activation barrier of approximately 1.6 kcal/mol[7]. Conversely, our simulations indicate a decrease in the rate-limiting activation barrier of 2.5 ± 0.8 kcal/mol. We posit that the small difference between experimental and theoretical values is most likely due to the conformational heterogeneity of the active site of the MTR1 ribozyme in solution. While our reaction simulations were carried out with ribose rings of C12 and U42 in the C3' endo and C2' endo conformation, respectively, these can likely also both assume the C3' endo state in solution as suggested by the results of classical MD simulations (Fig. 2). We hypothesize that initiating the reaction simulation for the MTR1wt system with both C12 and U42 in the C3' endo state would result in a lower rate-limiting activation barrier, more similar to that calculated for $MTR1m_2$. Consequently, MTR1wt molecules may be able to assume a reaction-accelerating and decelerating conformation in solution with C12/U42 ribose rings in the C3'/C3' endo state or C3'/C2' endo state, respectively. This may be the reason why the experimentally observed increase in the reaction rate is lower than that predicted computationally, as experimentally determined reaction rates correspond to ensemble averages over all conformations in solution.

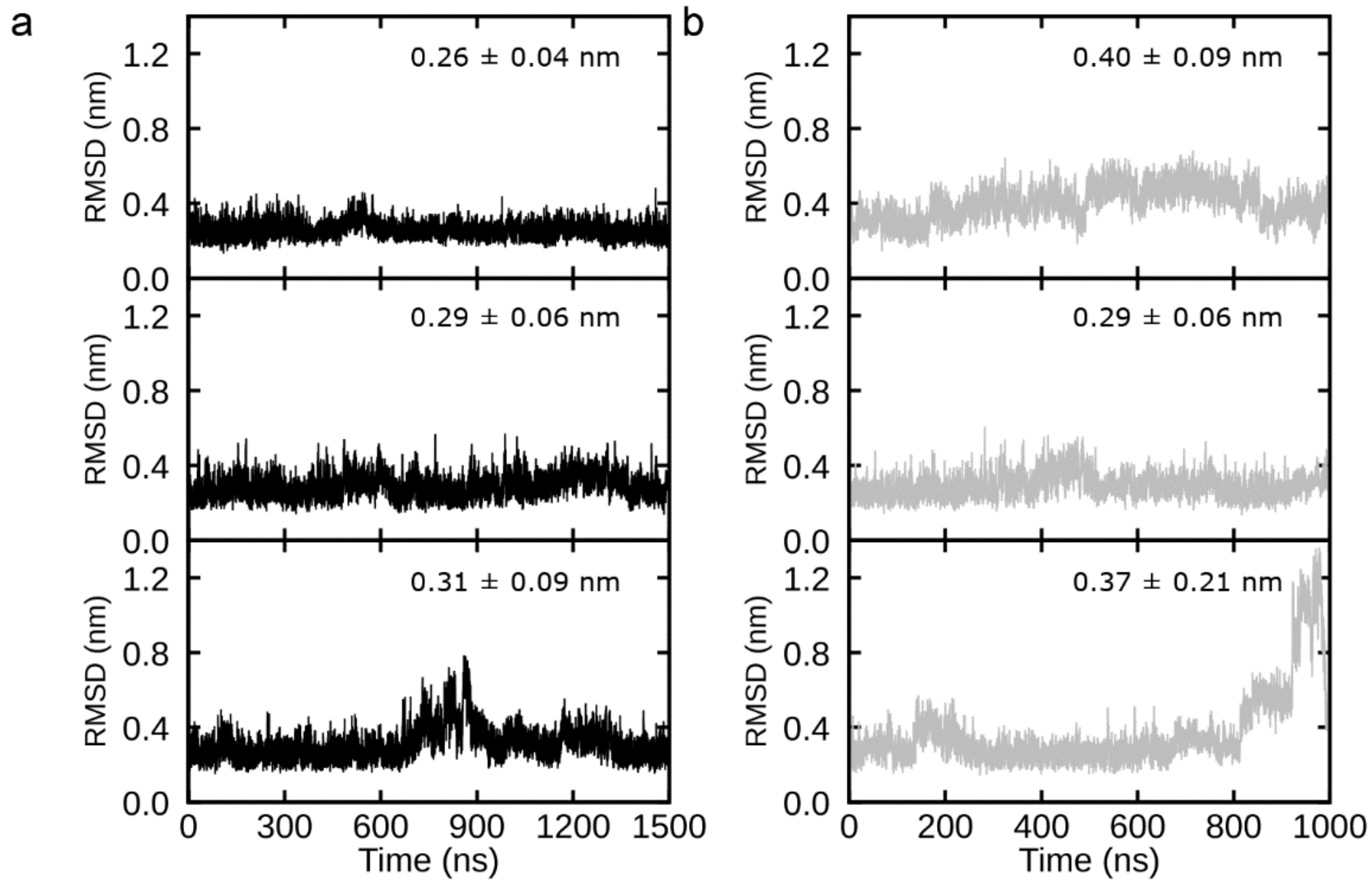

Figure S1. Backbone root mean square deviation (RMSD) as a function of simulation time. (a) RMSD as observed from classical molecular dynamics (MD) simulations of MTR1wt. RMSD was calculated with MTR1wt crystal structure as the reference state. RMSD is stable along all three simulation replicas. The average RMSD for each replica is given at the top right of each panel. (b) RMSD as observed from classical MD simulations of MTR1m$_2$. RMSD was calculated with MTR1wt crystal structure as the reference state. The three panels correspond to three simulation replicas and show the ribozyme global structure remains stable during MD simulations. The average RMSD ± s.d. across n = 15000 (a) or 10000 (b) simulation frames is given at the top right of each panel. Source data are provided as a Source Data file.

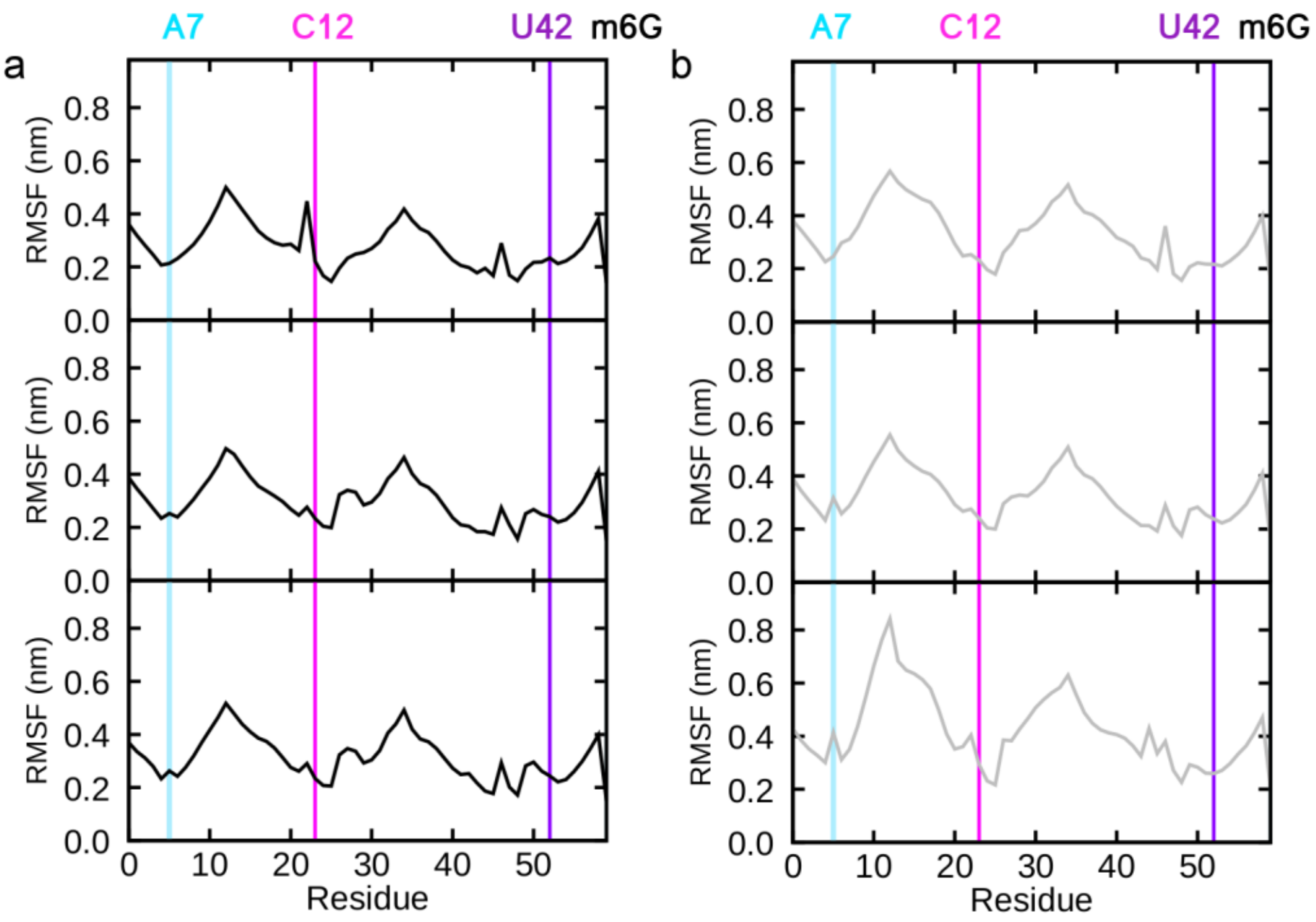

c

	MTR1wt	MTR1m2
A7	0.24 ± 0.03 nm	0.32 ± 0.08 nm
C12	0.23 ± 0.01 nm	0.25 ± 0.03 nm
U42	0.24 ± 0.01 nm	0.24 ± 0.02 nm
m6G	0.15 ± 0.01 nm	0.17 ± 0.03 nm

Figure S2. Residue root mean square fluctuations (RMSF) as observed during classical MD simulations. (a) RMSF calculated for three simulation replicas of MTR1wt. (b) RMSF calculated for three simulations replicas of MTR1m$_2$. Residue index depicted on the x-axis corresponds to numbering in the simulation box and not in the PDB structure of MTR1. For clarity, vertical lines mark the position of active site residues A7, C12 and U42, while m^{6}G cofactor corresponds to the final residue. Active site residues, including m^{6}G, are located in the central region of the ribozyme that shows the lowest RMSF. Conversely, the peaks in RMSF values correspond to ribozyme terminal arms. (c) Average RMSF ± s.d. of active site residues across n = 3 independent simulation replicas. Source data are provided as a Source Data file.

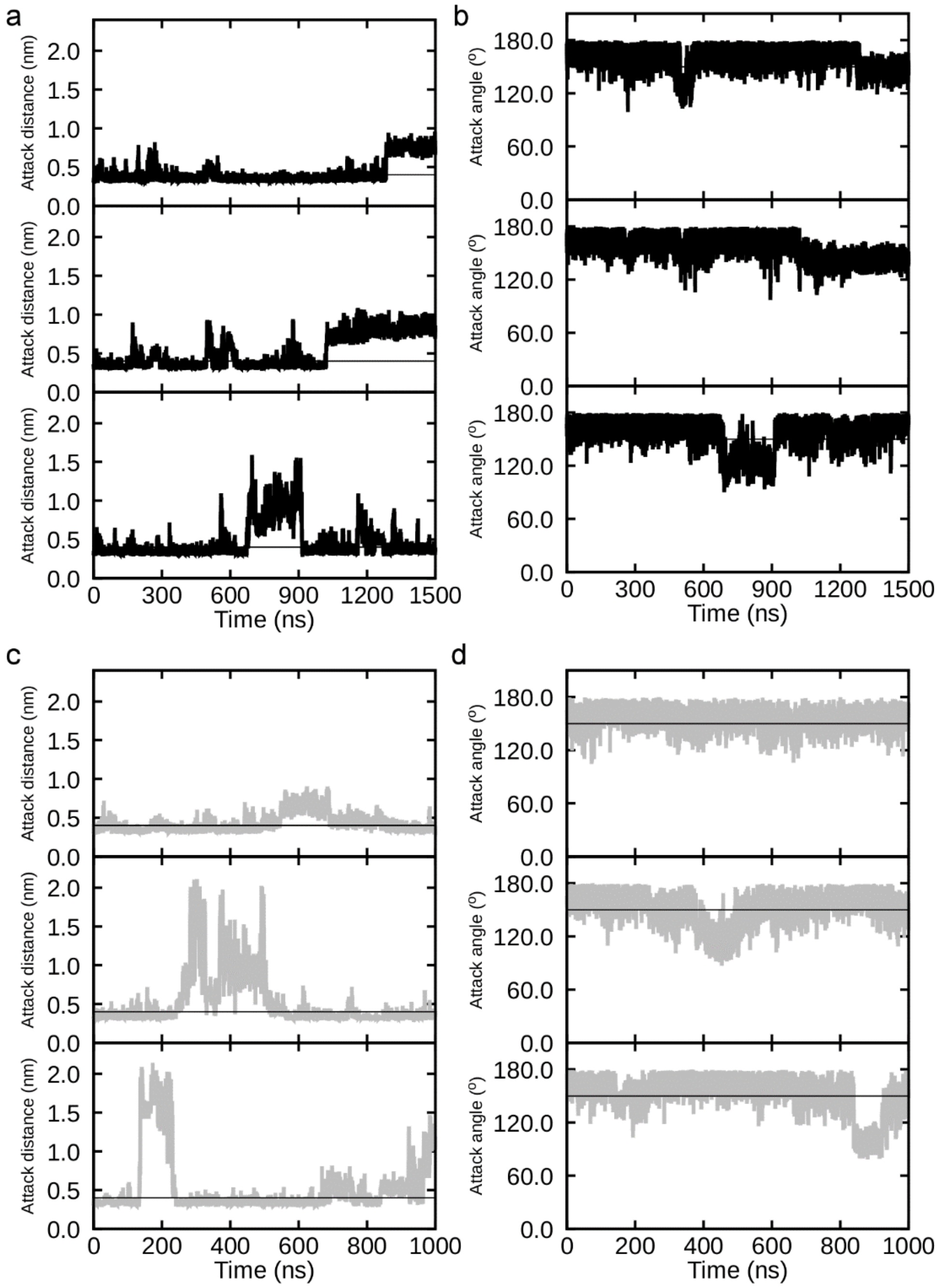

Figure S3. Attack distance and angle observed during classical MD simulations. (a, b) Attack distance and angle observed for MTR1wt as a function of simulation time. Each panel corresponds to a single simulation replica. Attack distance is defined as the distance between A7-N1 and m^{6}G-CM1 atoms, while attack angle is defined as the angle between A7-N1, m^{6}G-CM1 and m^{6}G-O6 atoms. The ribozyme is assumed to be in an active configuration if the attack distance is below 0.4 nm and the attack angle is above 150°. These thresholds are depicted with a vertical line. (c, d) Attack distance and angle observed for three simulation replicas of MTR1m$_2$ as a function of simulation time. Source data are provided as a Source Data file.

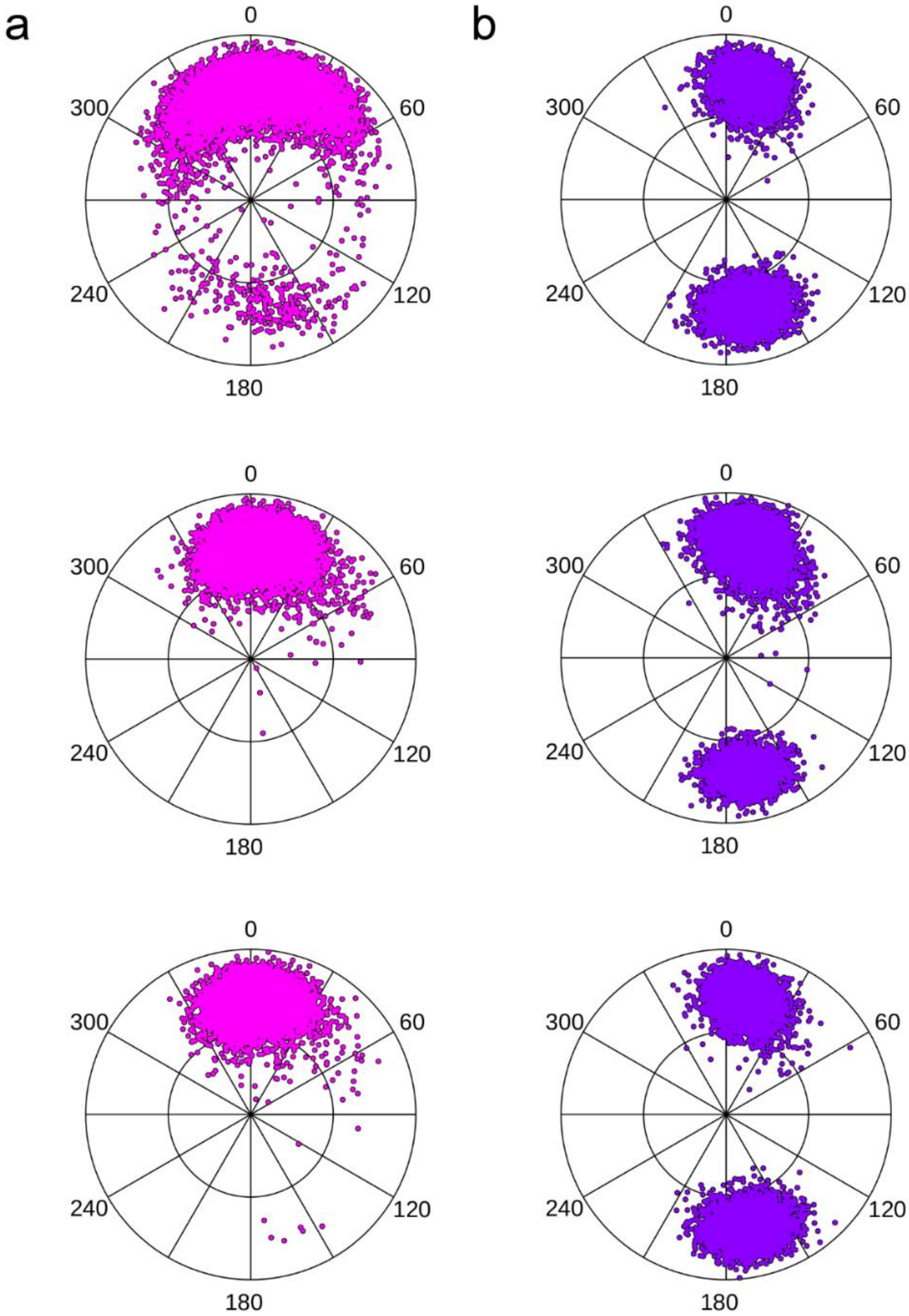

Figure S4. Pseudorotational wheel depicting the frequency of different ribose ring conformations of C12 and U42 observed during classical MD simulations of MTR1wt. The angle given in degrees corresponds to the pseudorotational angle, while the radial distance represents the puckering amplitude. (a) Pseudorotational wheel calculated for C12. In all three replicas, C12 predominantly assumes the C3' endo-C2' exo conformation, corresponding to pseudorotational angle range from -30° to 30°. (b) Pseudorotational wheel calculated for U42. U42 is shifting between the C3' endo-C2' exo and C2' endo-C3' exo conformation, corresponding to pseudorotational angle range from 150° to 210°, in all three replicas. Source data are provided as a Source Data file.

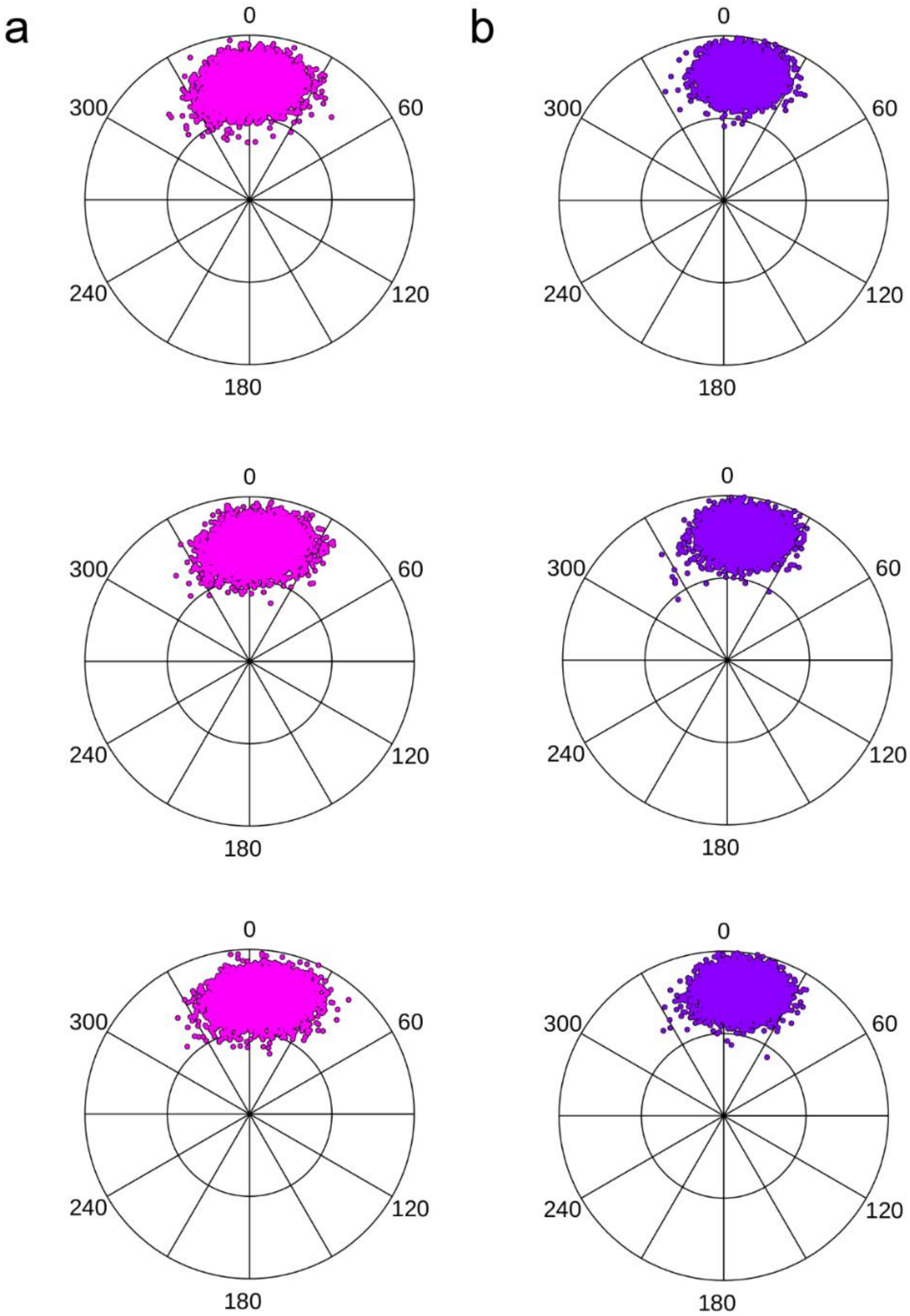

Figure S5. Pseudorotational wheel depicting the frequency of different ribose ring conformations of Cm12 and Um42 observed during MD simulations of MTR1m$_2$. The angle given in degrees corresponds to the pseudorotational angle, while the radial distance represents the puckering amplitude. (a) Pseudorotational wheel calculated for Cm12. Cm12 predominantly assumes the C3' endo-C2' exo conformation, corresponding to pseudorotational angle range from -30° to 30°, in all three replicas. (b) Pseudorotational wheel calculated for Um42. Um42 is present in the C3' endo-C2' exo conformation in all three replicas. Source data are provided as a Source Data file.

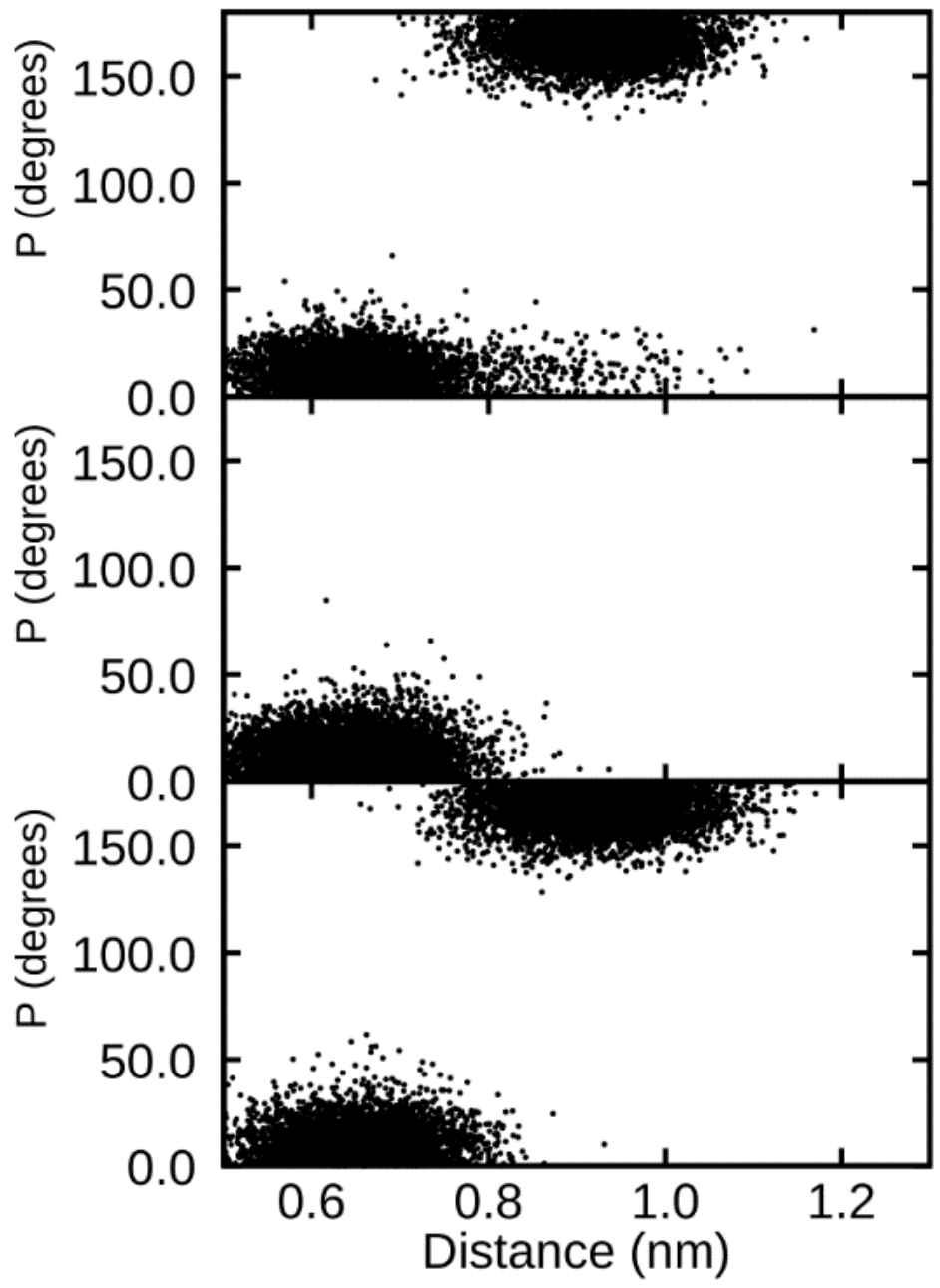

Figure S6. Pseudorotational angle of U42 is correlated to the distance between C12-2'O and U42-2'O atoms. Correlation is shown for the MTR1wt system. When U42 is present in the C3' endo conformation the distance between C12-2'O and U42-2'O atoms is shorter (0.6 nm - 0.8 nm), while the shift of the U42 ribose ring to the C2' endo conformation leads to an elongation of this distance (0.8 nm – 1 nm). Source data are provided as a Source Data file.

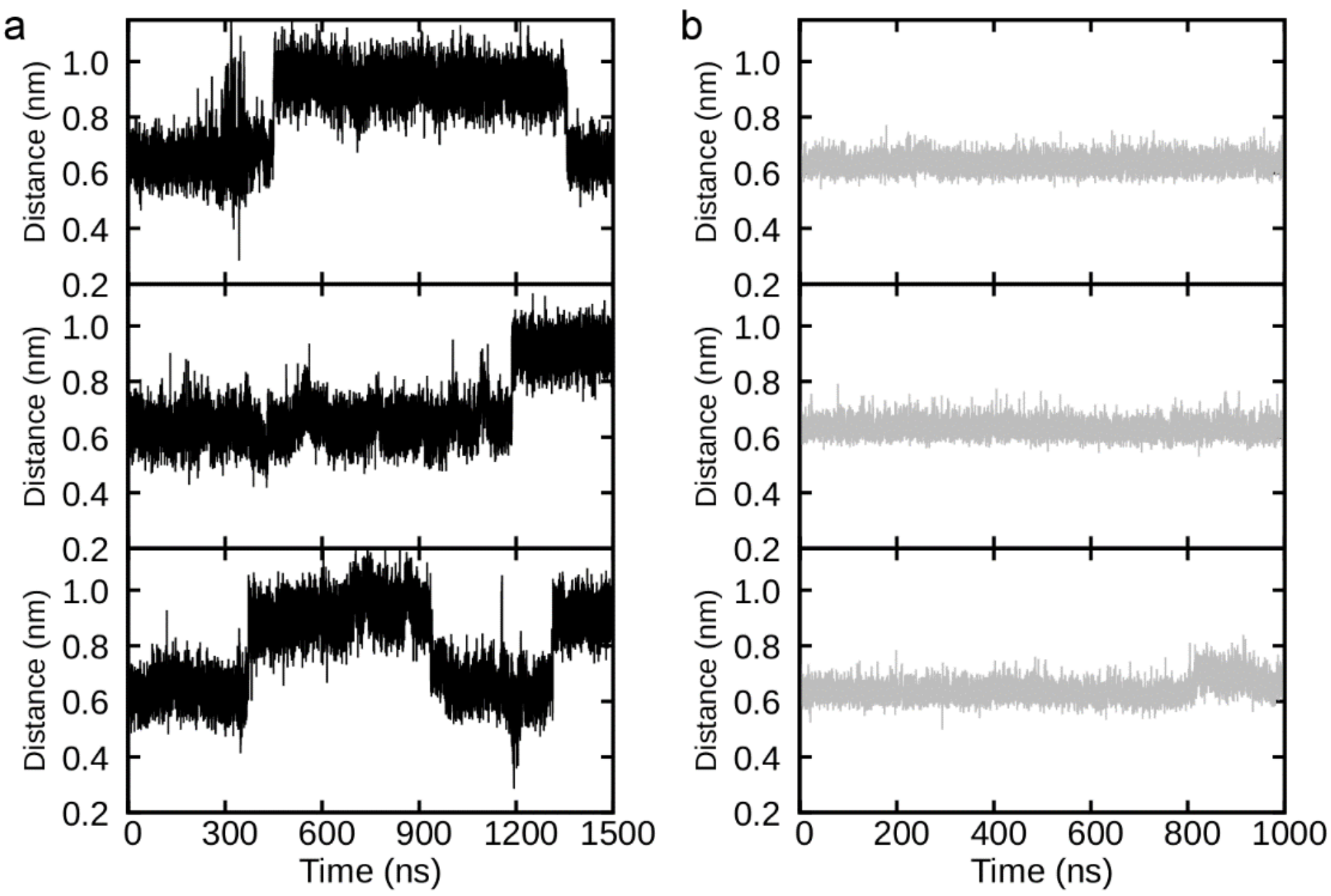

Figure S7. Distance between C12-2'O and U42-2'O atoms as observed during classical MD simulations. (a) In all three replicas, the distance between C12-2'O and U42-2'O atoms in the MTR1wt system shifts between low and high values, due to changing ribose ring puckering of U42. (b) The distance between Cm12-2'O and Um42-2'O atoms in the MTR1m$_2$ system is stable, as Um42 maintains the C3' endo conformation. Source data are provided as a Source Data file.

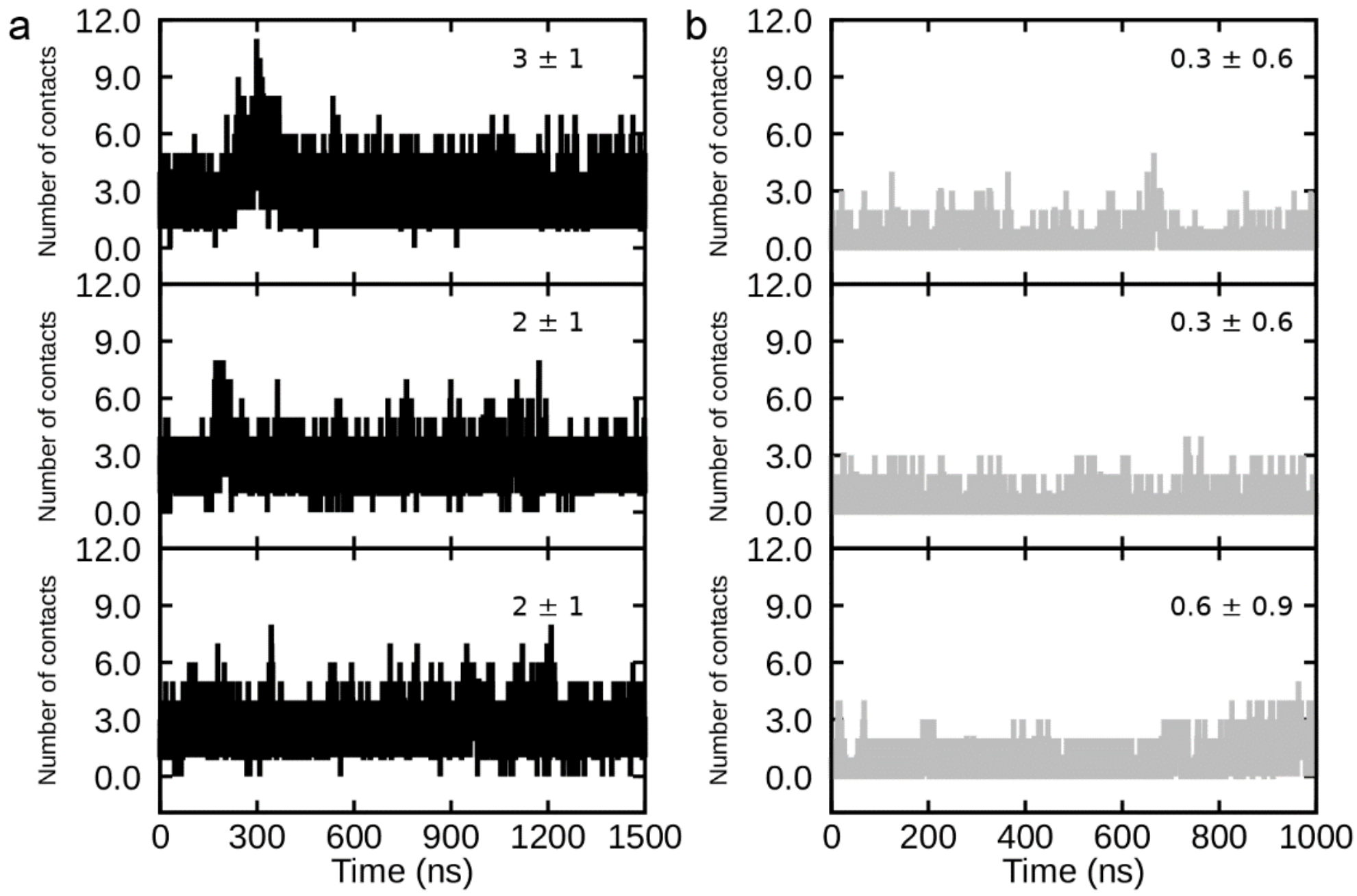

Figure S8. The number of water molecules surrounding the m^{6}G amino group observed during classical MD simulations. The average number of proximal water molecules ± s.d. across n = 15000 (a) or 10000 (b) simulation frames is written top right for each replica. (a) In the MTR1wt system, the higher average between 2'-OH groups of C12 and U42 results in higher number of waters approaching m^{6}G in all three replicas. (b) In MTR1m$_2$, the tight van der Waals interaction between 2'-OMe groups of Cm12 and Um42 prevents water molecules entering the catalytic site. Source data are provided as a Source Data file.

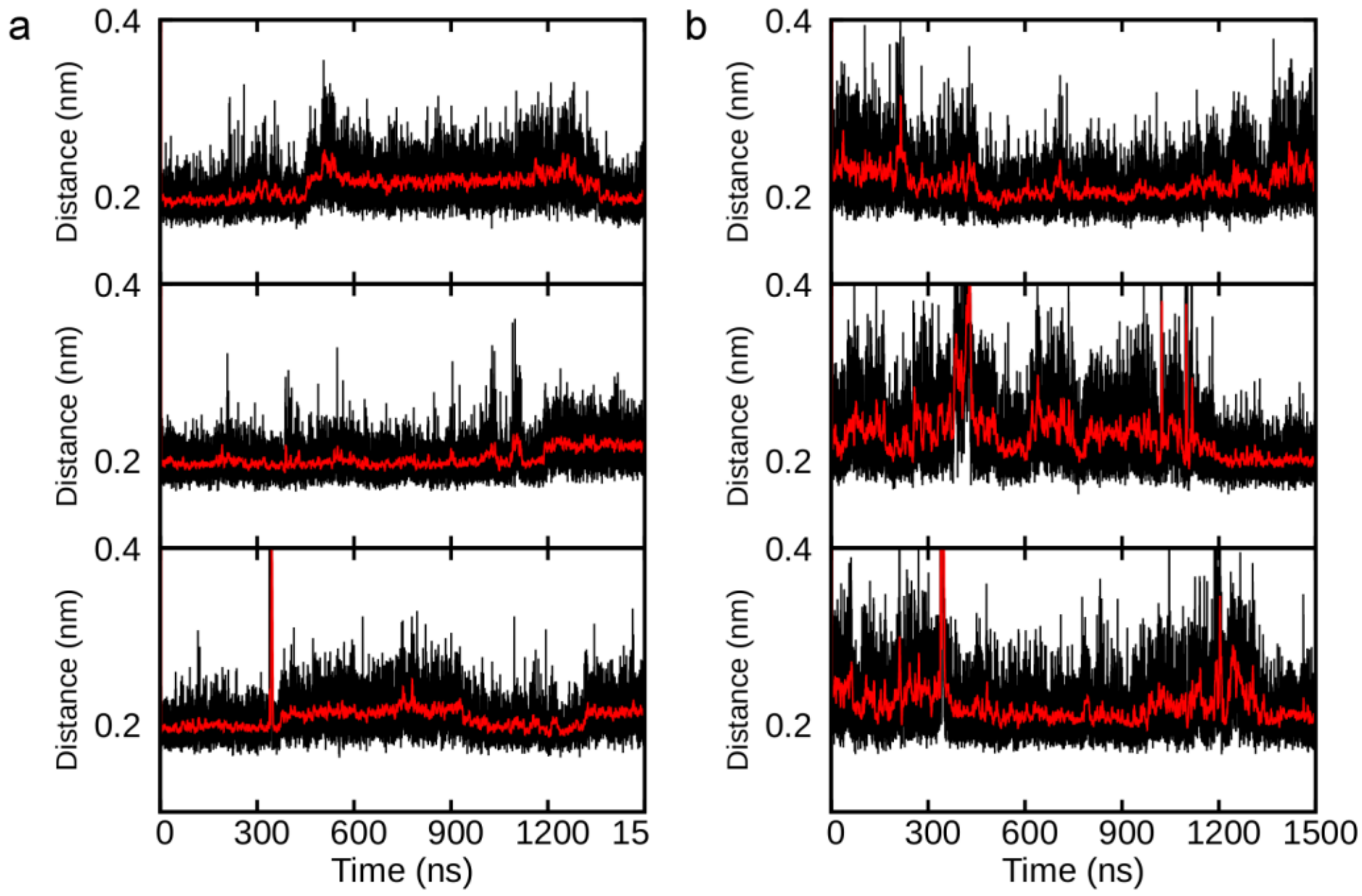

Figure S9. Distance between U42 and m^{6}G as observed during classical MD simulations of MTR1wt. (a) Distance between U42-O2 atom and m^{6}G amino group proton defining hydrogen bond 4 (hb4). (b) Distance between U42-O4 atom and m^{6}G-N9 proton defining hydrogen bond 6 (hb6). As U42 transition to the C2' endo conformation hb4 is destabilized, while hb6 is more frequently formed. Black line reports the instantaneous value, while the red line corresponds to the rolling average over 30 frames. Source data are provided as a Source Data file.

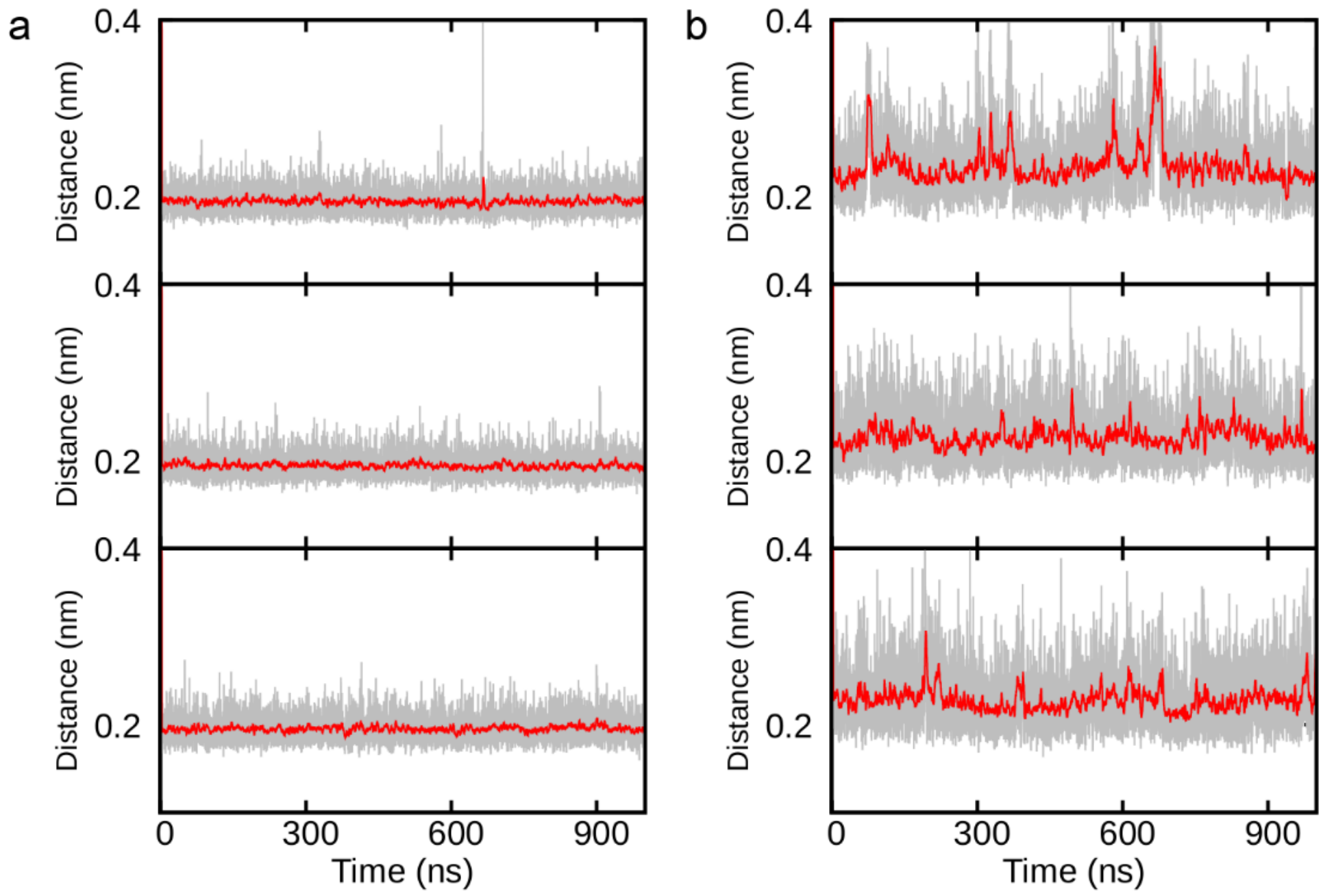

Figure S10. Distance between Um42 and m^{6}G as observed during classical MD simulations of MTR1m$_2$. (a) Distance between Um42-O2 atom and m^{6}G amino group proton defining hydrogen bond 4 (hb4). (b) Distance between Um42-O4 atom and m^{6}G-N9 proton defining hydrogen bond 6 (hb6). Um42 stably occupies the C3' endo conformation. Consequently, hb4 is stably formed, while hb6 is more labile. Gray line reports the instantaneous value, while the red line corresponds to the rolling average over 30 frames. Source data are provided as a Source Data file.

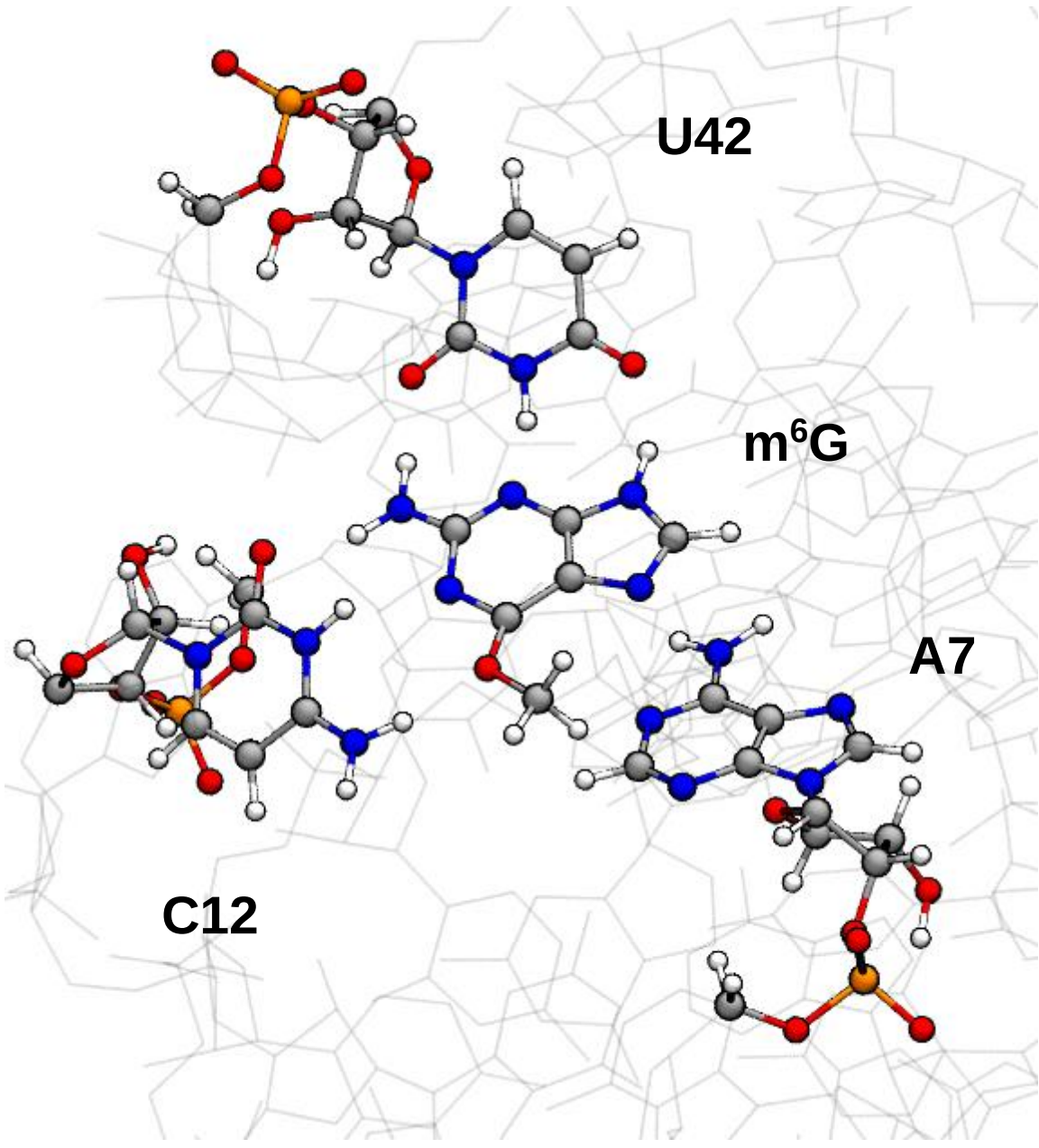

Figure S11. QM zone definition for hybrid quantum-classical (QM/MM) MD simulations. The figure depicts the active site of MTR1wt, with QM atoms shown in ball and stick representation. For each nucleotide (A7, C12 and U42), QM/MM boundary was set along the bond C4'-C5' and bonds C2'-C3' and C3'-C4' of the successive nucleotide. This QM zone definition was applied across all biased and unbiased QM/MM simulations. An analogous QM zone definition was employed also for MTR1m$_2$.

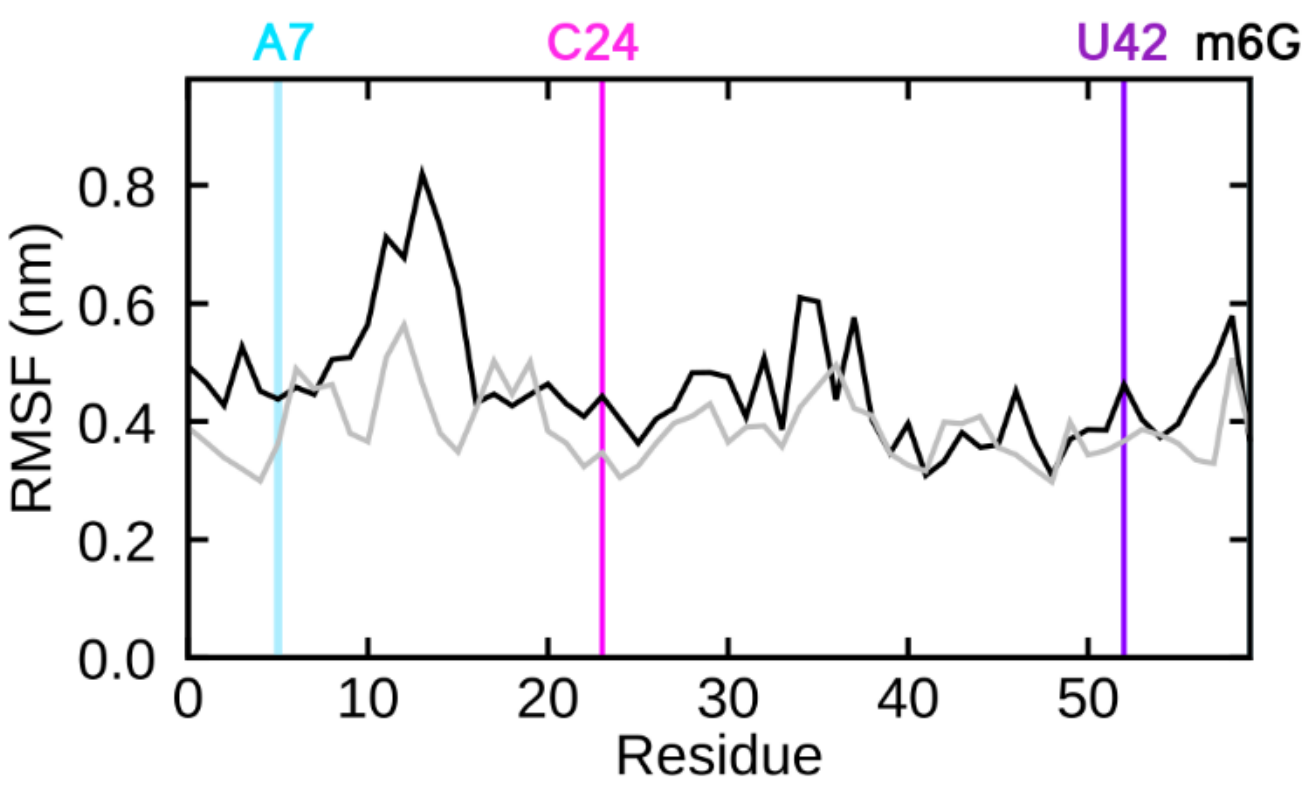

Figure S12. Root mean square fluctuation (RMSF) per residue observed during unbiased QM/MM MD simulations. (a) RMSF calculated for MTR1wt (black line) and MTR1m$_2$ (grey lines). Residue index depicted on x-axis corresponds to numbering in the simulation box and not in the PDB structure of MTR1. For clarity, vertical lines mark the position of active site residues A7, C12 and U42, while the m^{6}G cofactor corresponds to the final residue. Source data are provided as a Source Data file.

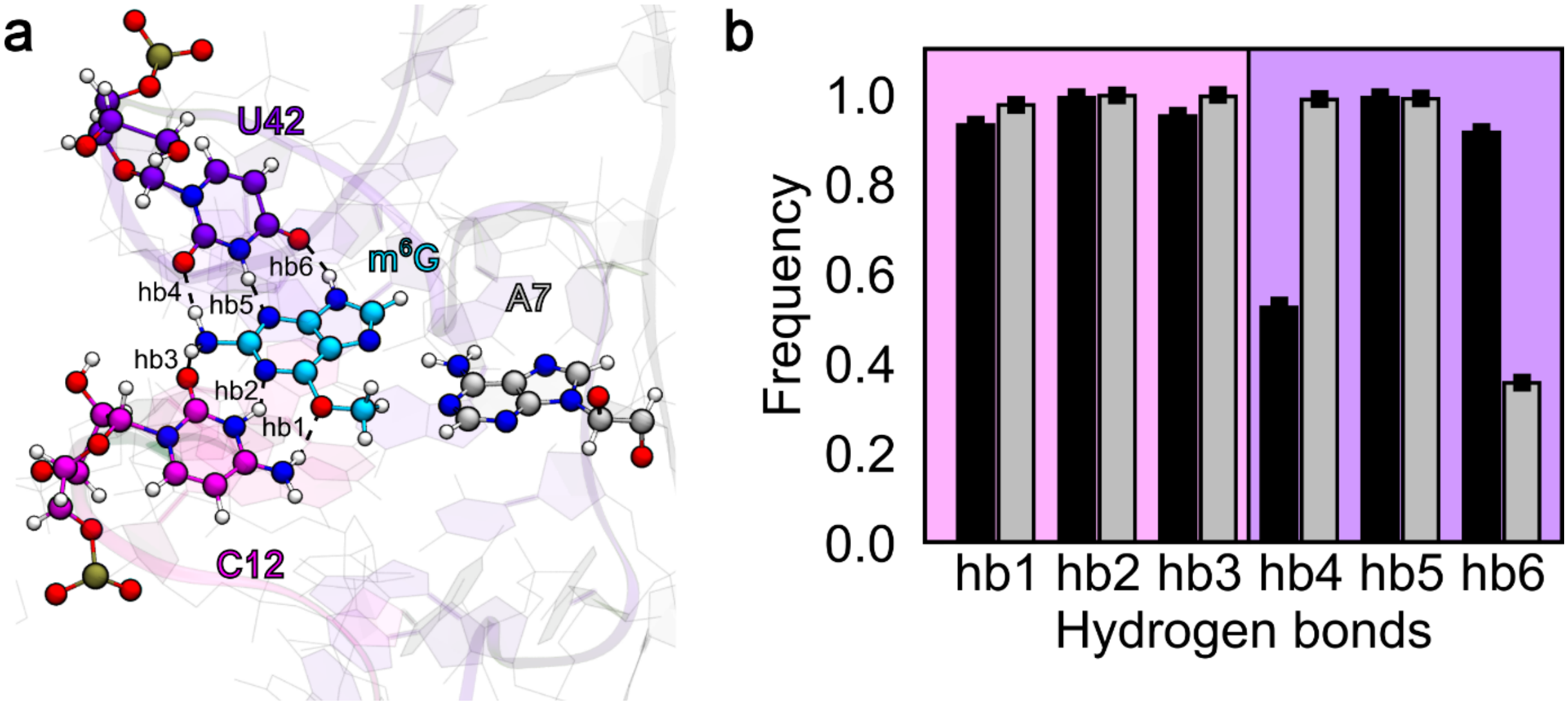

Figure S13. Comparison of average hydrogen bonding frequency between C12, U42 and m^{6}G observed during QM/MM MD simulations. (a) Scheme of hydrogen bond definition. (b) Frequency of hydrogen bonding observed for MTR1wt is shown with black bars, while grey bars correspond to results obtained for MTR1m$_2$. Source data are provided as a Source Data file.

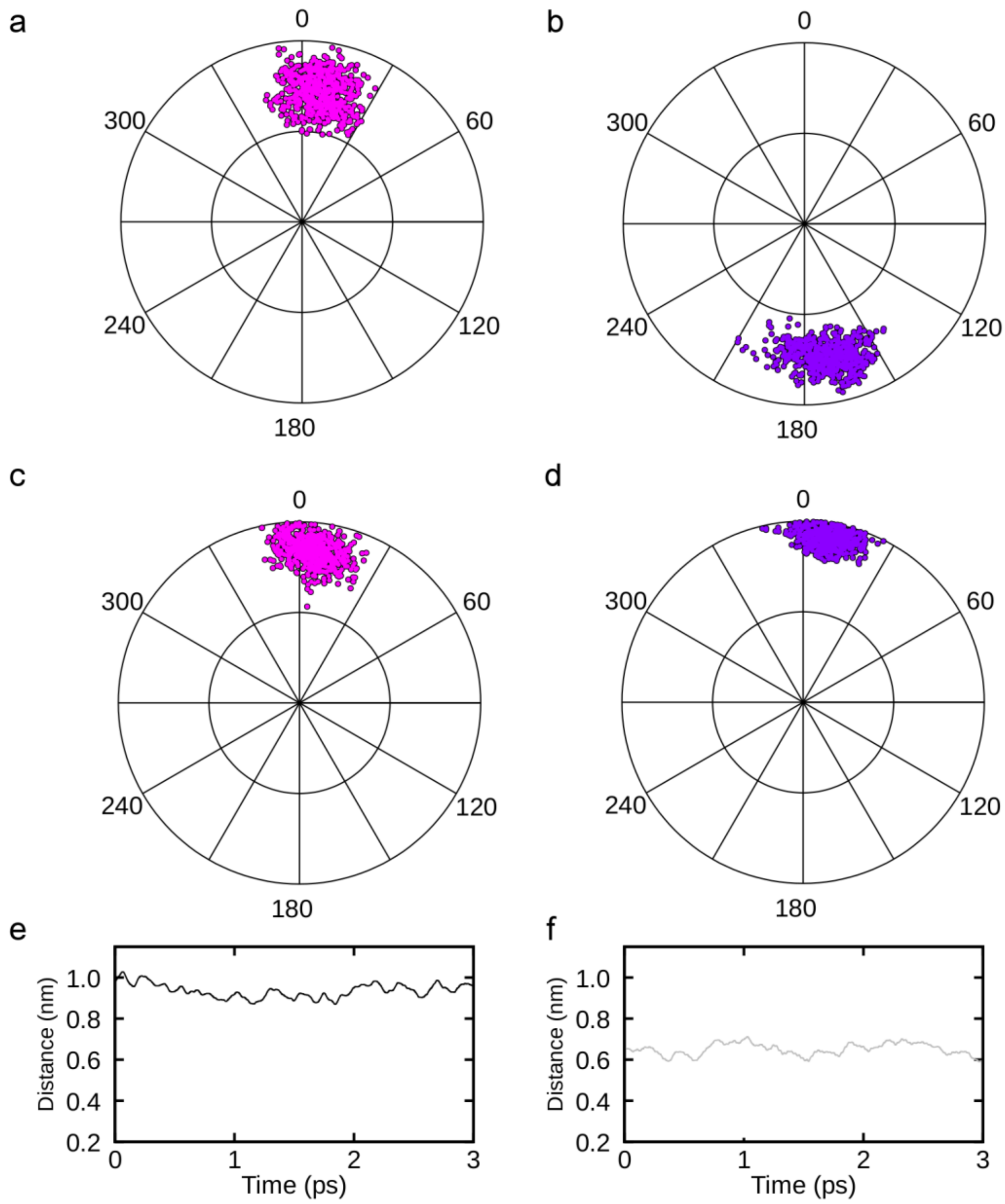

Figure S14. Sugar ring puckering as observed during unbiased QM/MM MD simulations. (a, b) Pseudorotational wheel calculated for C12 and U42 in MTR1wt. The angle given in degrees corresponds to the pseudorotational angle, while the radial distance represents the puckering amplitude. (c, d) Pseudorotational wheel calculated for Cm12 and Um42 in MTR1m$_2$. (e) The distance between C12-2'O and U42-2'O atoms in MTR1wt. (f) The distance between Cm12-2'O and Um42-2'O atoms in the MTR1m$_2$ system. Due to the short time scale of QM/MM MD simulations no changes in sugar ring puckering are observed. Accordingly, the distance between C12-2'O and U42-2'O atoms is stable. Source data are provided as a Source Data file.

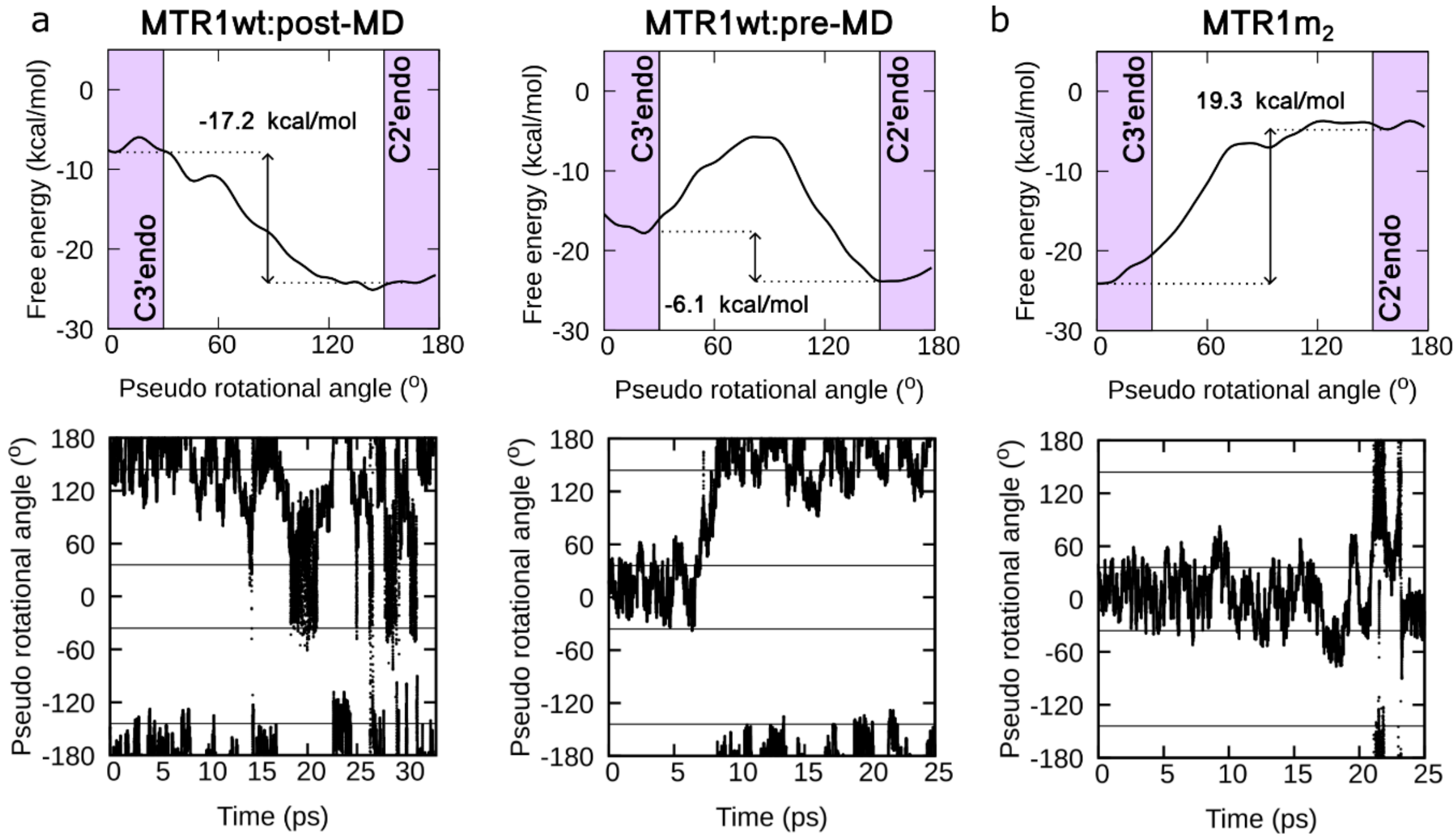

Figure S15. Free energy profile as a function of U42 or Um42 pseudorotational angle obtained with QM/MM metadynamics. (a) Free energy profile obtained for the MTR1wt system. If the simulations were initiated from the structure obtained after the production run of classical MD (post-MD), the C3' endo conformation is less stable than the C2' endo conformation for 17.2 ± 0.3 kcal/mol. Instead, if simulations were started from the relaxed crystal structure (pre-MD), the difference in stability between the C3' and C2' endo states was less pronounced ($\Delta F_{C3' \rightarrow C2'}$ = -6.1 ± 0.3 kcal/mol). Lower panels depict the pseudorotational angle of U42 as a function of simulation time. (b) Free energy profile obtained for the MTR1m$_2$ system. The C3 'endo conformation is more stable than the C2' endo conformation for 19.3 ± 0.2 kcal/mol. Lower panels depict the pseudorotational angle of Um42 as a function of simulation time. Source data are provided as a Source Data file.

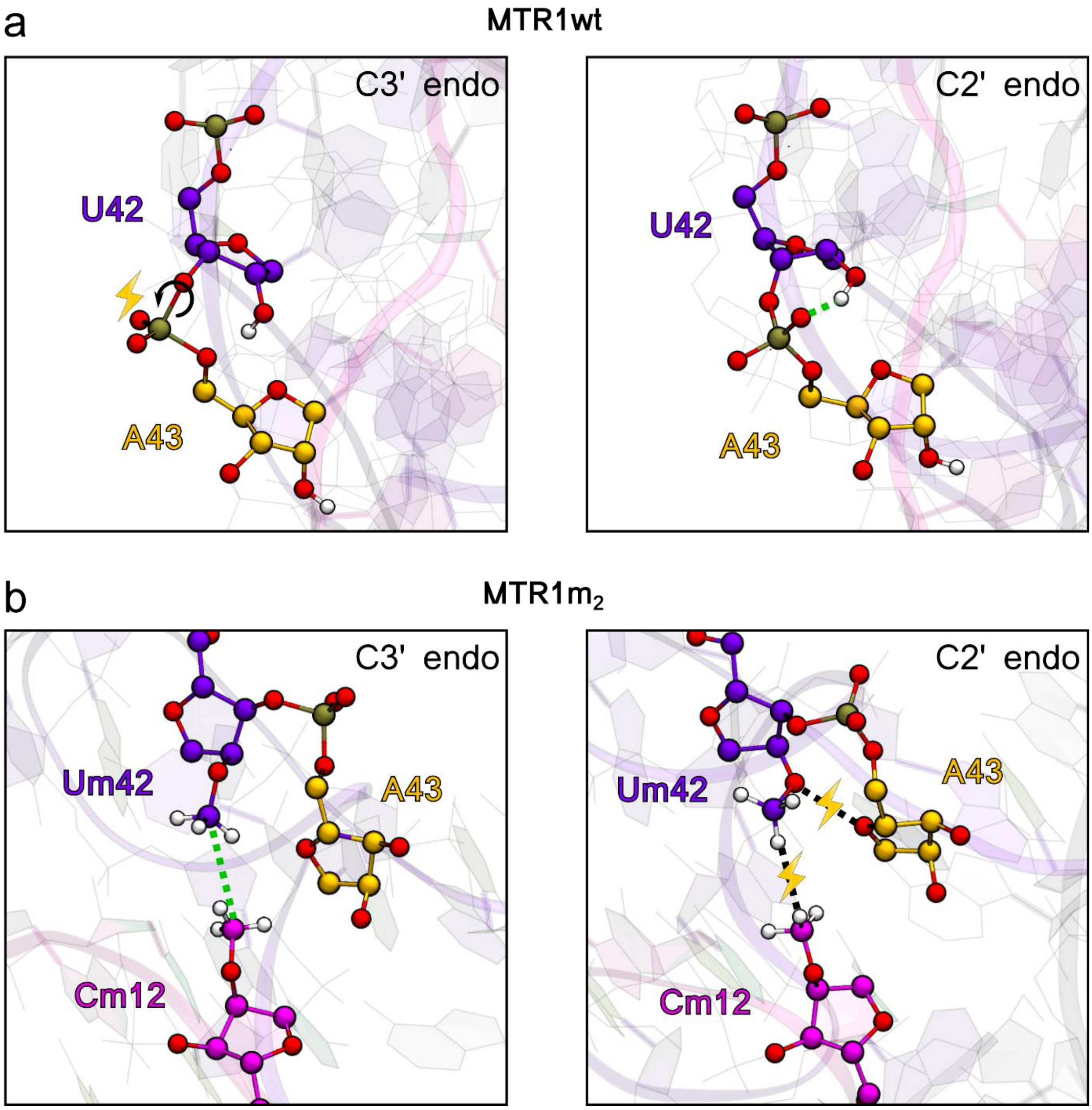

Figure S16. Representative snapshots from QM/MM metadynamics simulations. (a) In MTR1wt the C2' endo state of U42 is stabilized by a hydrogen bond between U42-2'OH and A43-OP2 (0.20 ± 0.05 nm). In the C3' endo state this bond is absent (0.34 ± 0.01 nm) with the phosphate group assuming a different torsion angle that leads to steric stress. (b) In MTR1m$_2$ the C3' endo state of Um42 is promoted by a linear positioning of 2'-OMe groups that results in a van der Waals interaction (green dotted line). When Um42 is in the C2' endo state the 2'-OMe groups lie at an angle that is less favourable for the van der Waals interaction and additionally results in a steric clash between the Um42-O2' and A43-O4' atoms (0.31 ± 0.02 nm). The bolt indicates unfavourable interactions.

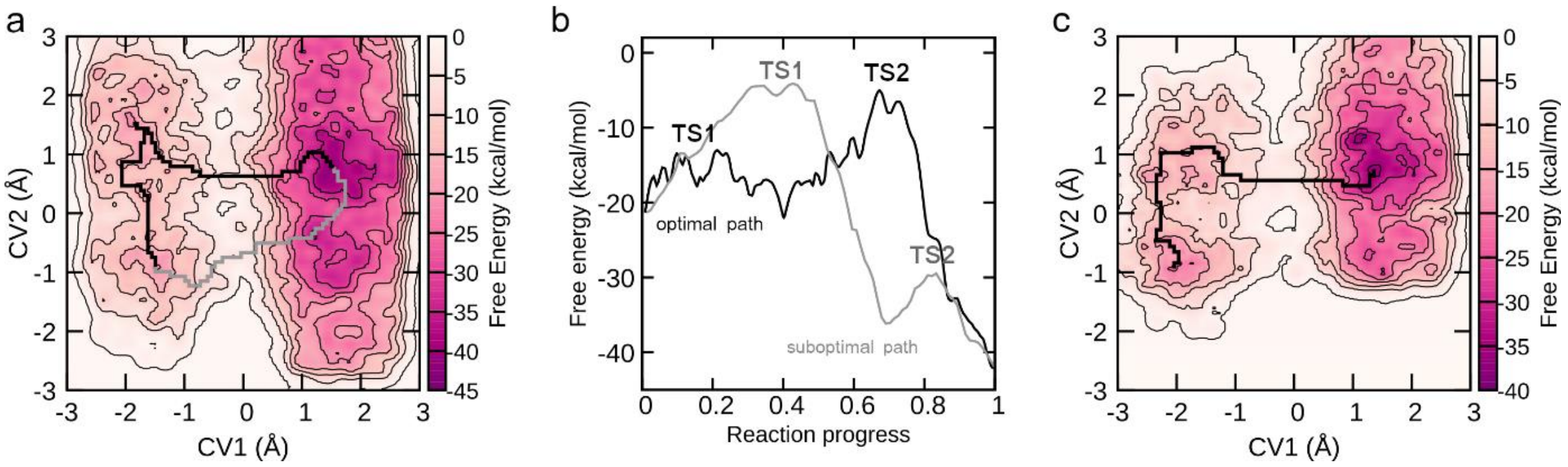

Figure S17. Reaction pathway of the MTR1-catalyzed methylation reaction as observed from QM/MM metadynamics. (a) Free energy surface for the methylation reaction in the MTR1wt system. Minimum energy reaction pathway is shown in black. Alternative suboptimal pathway where methylation occurs prior to the proton transfer is shown in grey. (b) Free energy profile for the optimal and suboptimal reaction pathways shown in panel a. The activation barrier to achieve the first transition state is ~ 8.4 kcal/mol for the optimal path and ~ 17 kcal/mol for the suboptimal path. (c) Free energy surface for the methylation reaction in the MTR1m$_2$ system. Minimum energy reaction pathway is shown in black. Source data are provided as a Source Data file.

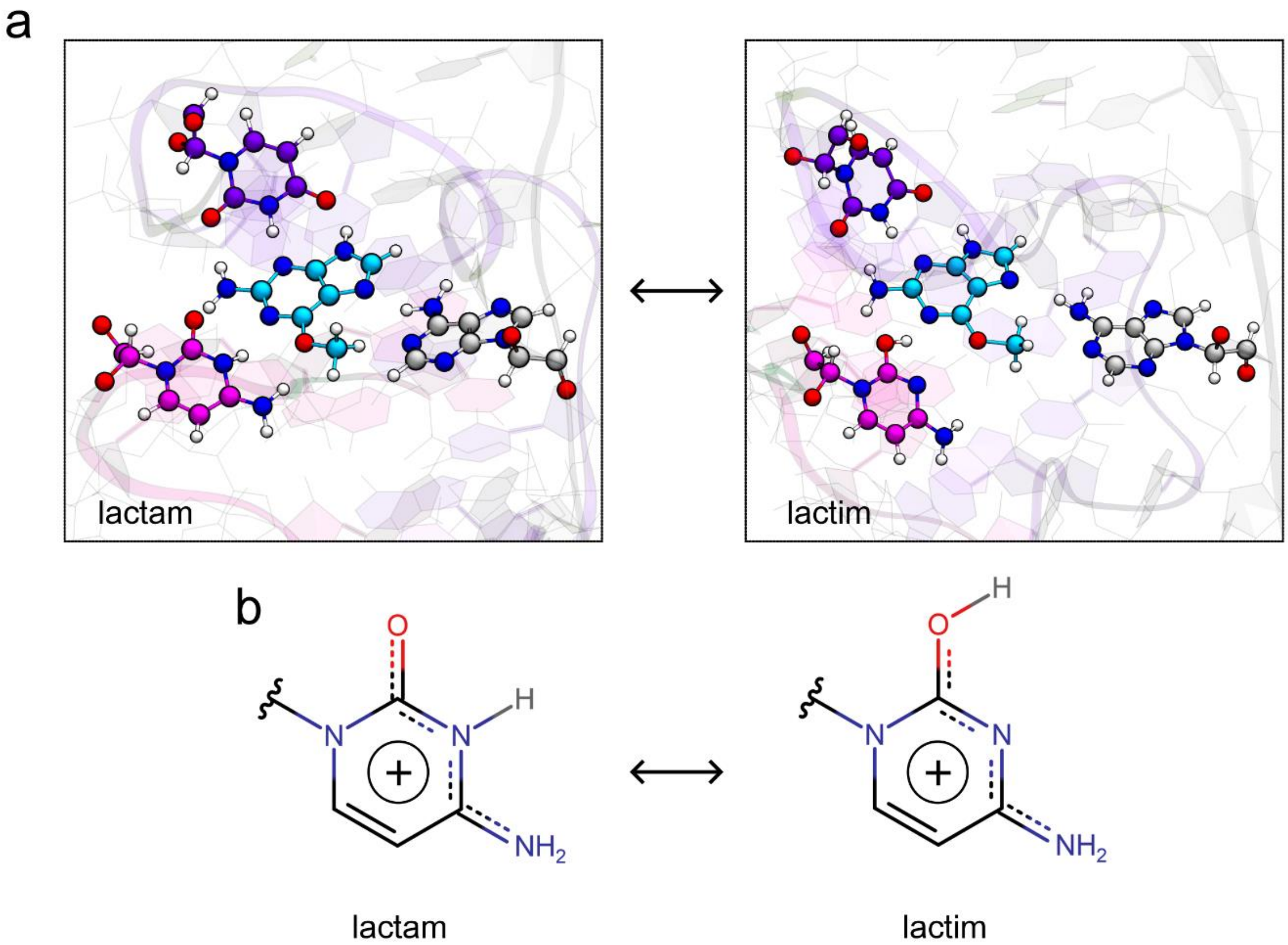

Figure S18. C12 lactam-lactim tautomerism in MTR1wt. (a) Snapshots of the MTR1wt active site in the reactant state with C12 in the lactam or lactim conformation. (b) Chemical scheme of the protonated cytidine nucleobase in the lactam and lactim state.

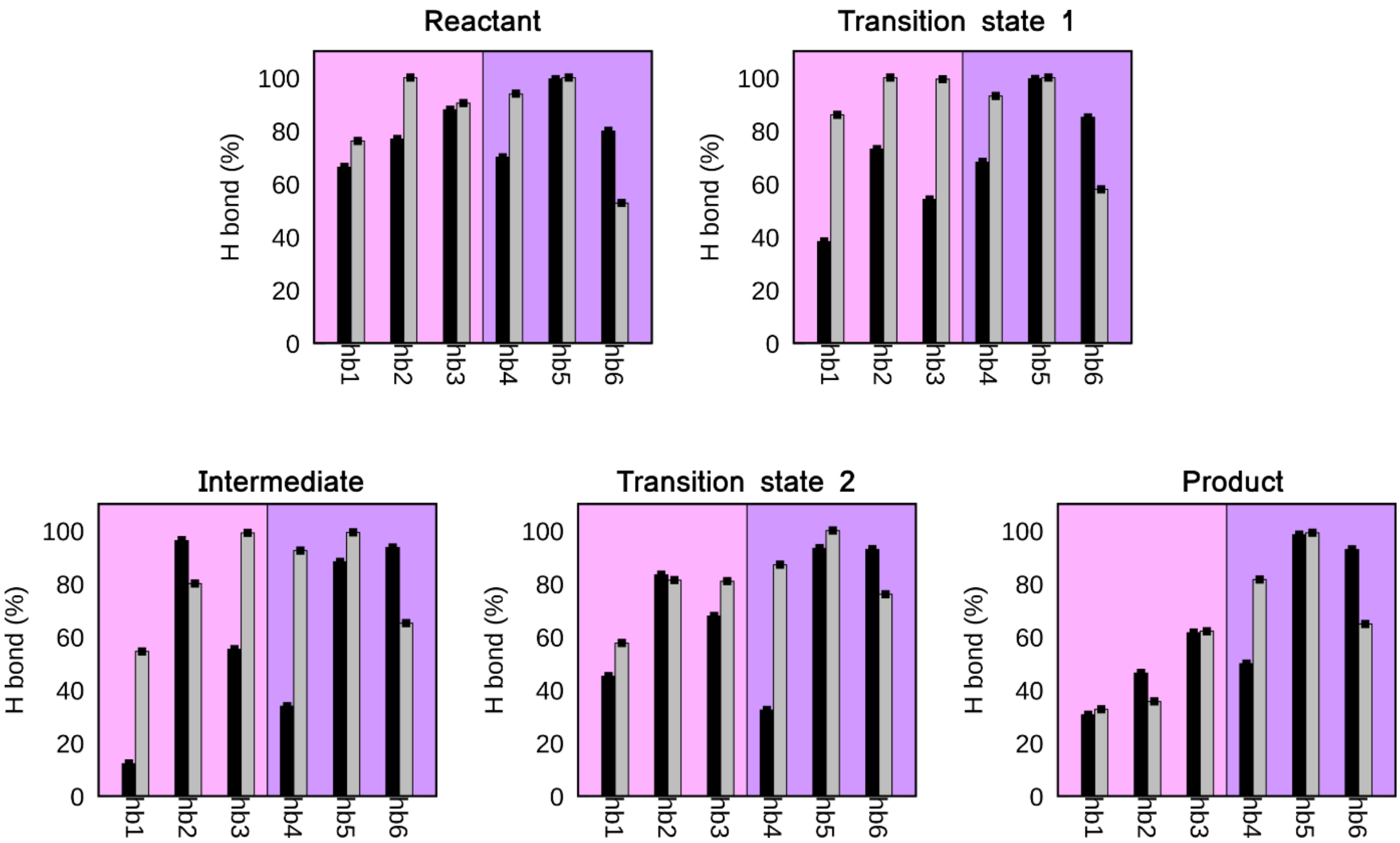

Figure S19. Hydrogen bonding frequency between C12, U42 and m^{6}G observed during QM/MM metadynamics. Hydrogen bonding frequency is depicted for each state along the reaction. Frequency of hydrogen bonding observed for MTR1wt is shown with black bars, while grey bars correspond to results obtained for MTR1m$_2$. Hydrogen bonds are defined as in Figure S12. Source data are provided as a Source Data file.

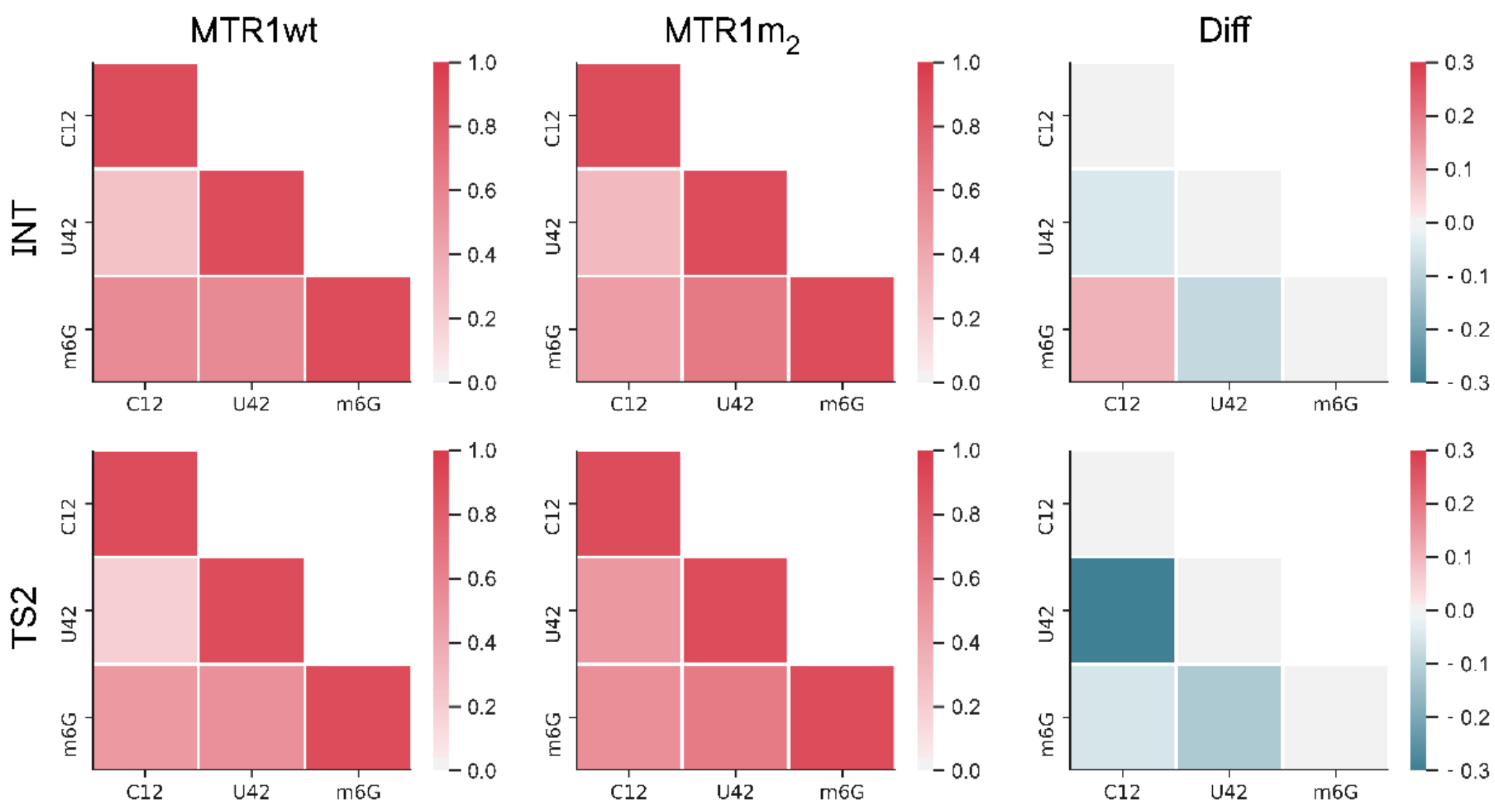

Figure S20. Correlation coefficients between C12, U42 and m⁶G observed during QM/MM metadynamics simulating the methylation reaction. Correlations were estimated in the intermediate (INT) and transition state 2 (TS2) for MTR1wt and MTR1m$_2$. The plots on the right depict the difference in correlation between MTR1wt and MTR1m$_2$ for the individual states. The active site in MTR1wt shows lower correlation than in the MTR1m$_2$ system in both states. Source data are provided as a Source Data file.

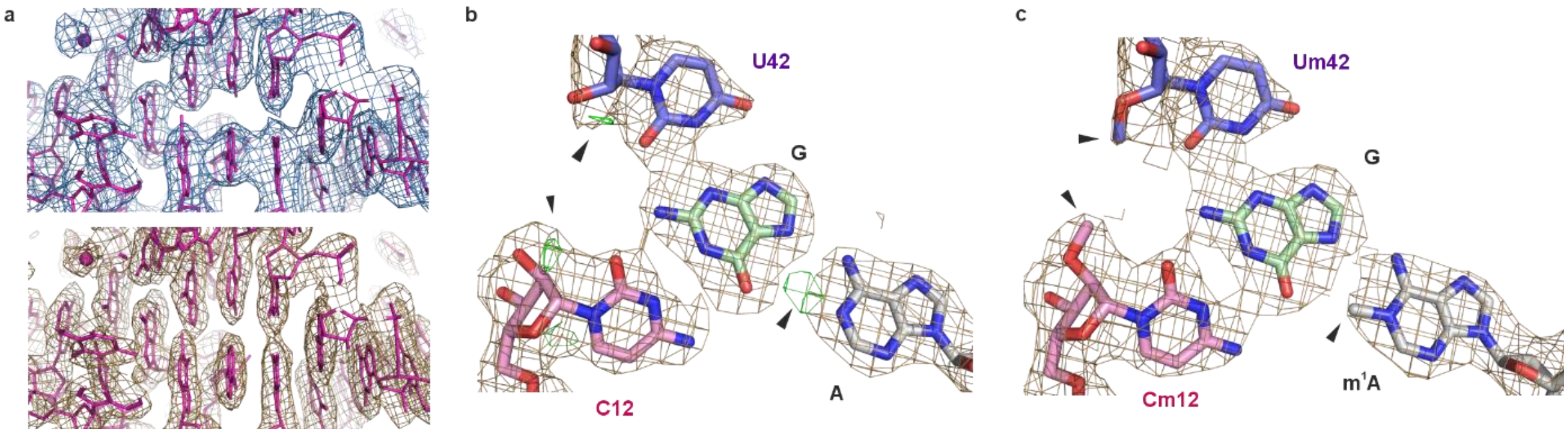

Figure S21. Density modification and modelling of the three methyl groups. (a) Molecular replacement was performed with unmethylated MTR1wt to reduce bias (all unmodified including A7, C12 and U42, PDB:7Q7X). The initial map showed a continuous density along the base stacking (upper, blue mesh), which was improved significantly by density modification (see Methods) and the resulting base densities were well separated (lower, beige mesh). (b) After a few rounds of refinements with all unmodified nucleotides (A7, C12, U42), positive difference density was steadily observed near the 2'O of C12 and U42 and N1 of the target adenosine (black arrows), where methyl groups were placed in the model accordingly (c). In (a), blue and beige mesh represent the σA-weighted 2Fo-Fc density map countered at 1σ. In (b) and (c), beige and green mesh depict the σA-weighted 2Fo-Fc (1σ) and Fo-Fc (3σ) density map respectively.

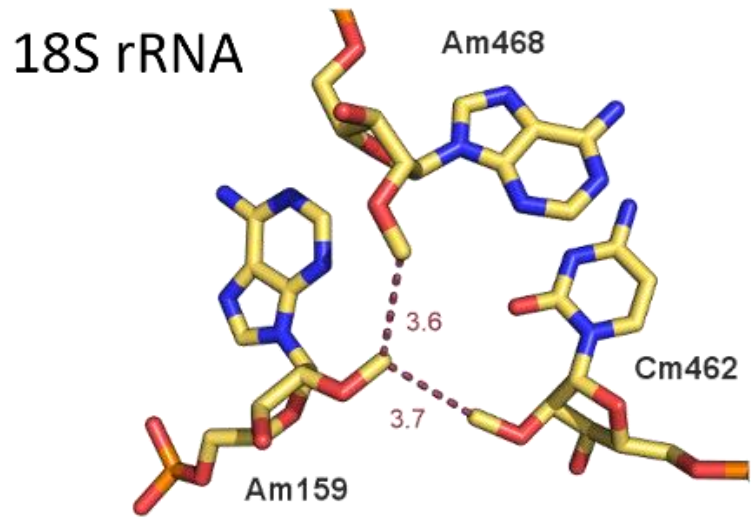

Holvec et al Nat Struct Mol Biol. 2024

Figure S22. Contacts between 2'-OMe groups stabilize 18S rRNA in human ribosome. Three 2’-OMe groups in human 18S rRNA show similar distance as found between the two 2’-OMe groups in $MTR1m_2$.

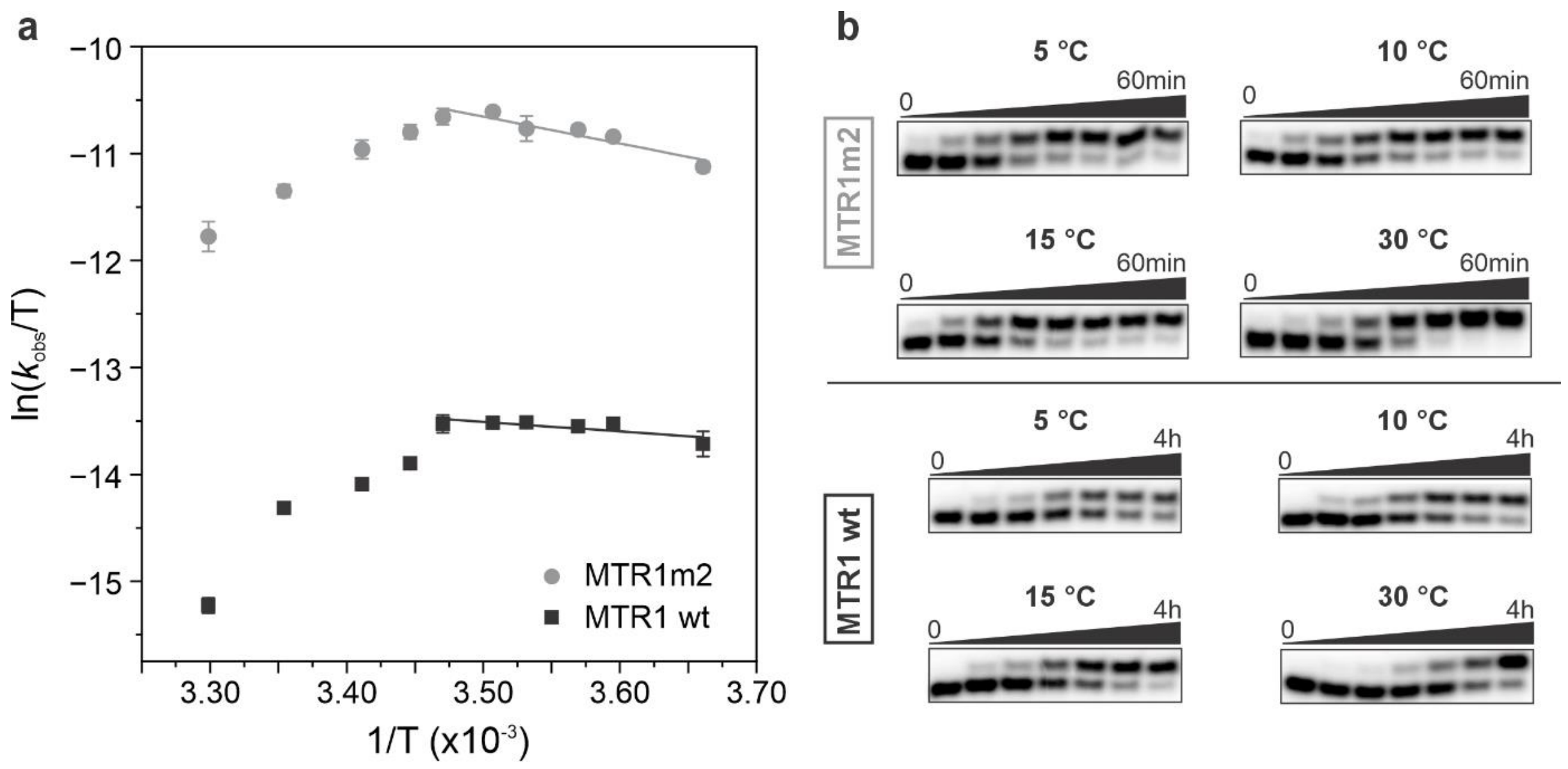

Figure S23. Temperature dependence of MTR1wt and MTR1m$_2$. (a) Eyring equation plots for the methyltransferase reaction catalyzed by MTR1m$_2$ and MTR1wt. Linear fit was applied for data collected from reactions performed at 0 – 15 °C. Reactions at above 25°C exhibited an opposite trend likely due to weaker hybridization of substrate and ribozyme at higher temperatures. Respective k_{obs} values are summarized in Table S3. Values for $\Delta H^{\ddagger}$ and $\Delta S^{\ddagger}$ can be determined from the slope and the y-intercept of the linear fits and are summarized in Table S4. Error bars denote ± s.d. of the mean for n = 2 independent experiments. (b) Representative PAGE images of kinetic assays performed to obtain data shown in (a). Time points were taken at 0, 45 sec, 2 min, 5 min, 10 min, 20 min, 35 min and 60 min for MTR1m$_2$, or 0, 5 min, 10 min, 30 min, 1 h, 2 h, 4 h for MTR1 wt. Source data are provided as a Source Data file.

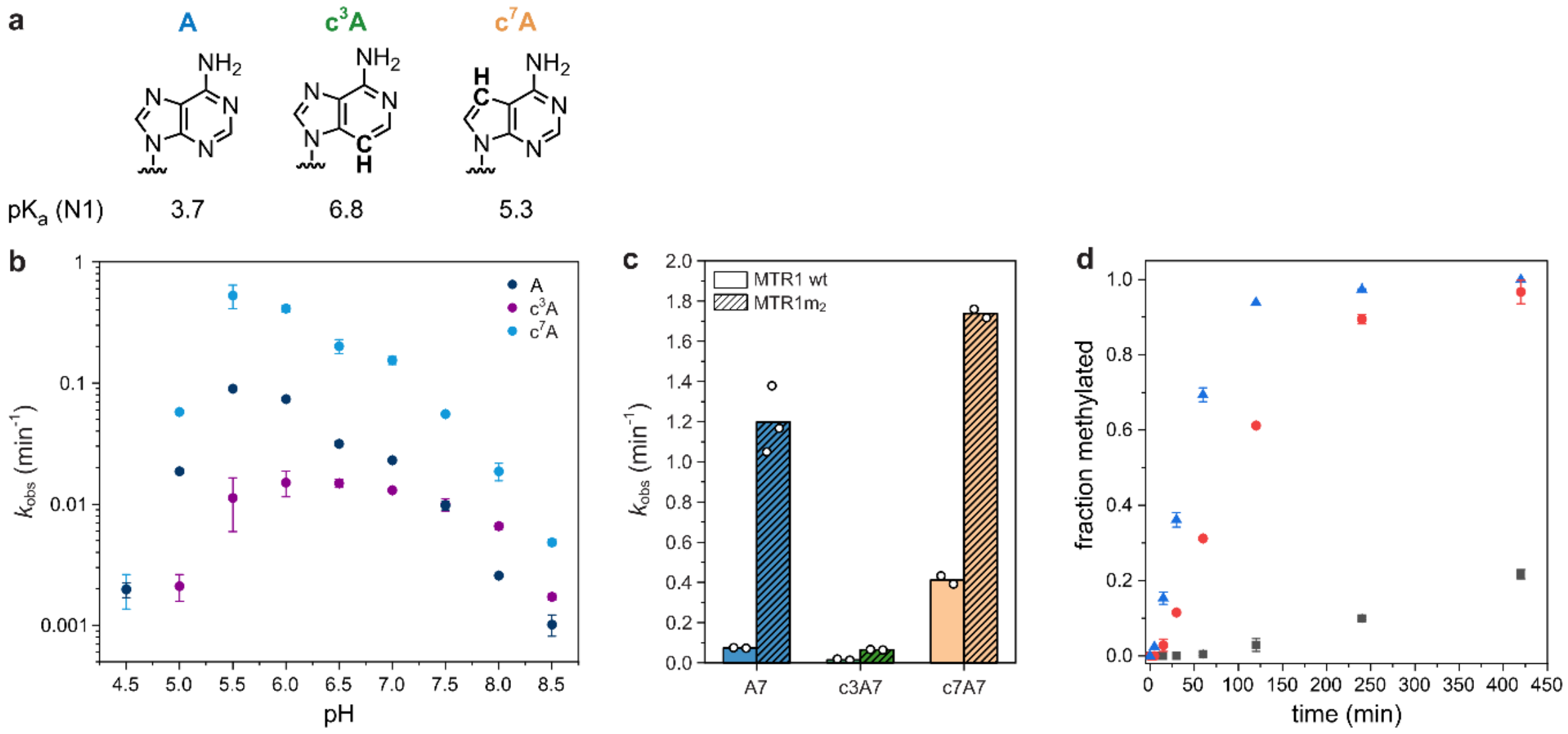

Figure S24. Kinetics and multiple turnover of MTR1m₂. (a) Structures of A, c^3A and c^7A together with the pKa of the free nucleosides. (b) Comparison of kinetic rate constants for the methylation of A, c^3A or c^7A by MTR1wt (100 µM m6G, 40 mM $MgCl_2$, 25 °C). Error bars denote ± s.d. of the mean for n = 2 or 3 independent replicates. (c) Acceleration of reaction rate for methylation of A, c^3A or c^7A by $MTR1m_2$ compared to MTR1wt at pH 6.0; individual data points are shown for n = 2 or 3 independent replicates. (d) Multiple turnover of $MTR1m_2$ with 1% (black), 5% (red), 10 mol% (blue) ribozyme ($MTR1m_2$, pH 6.0). Error bars denote ± s.d. of the mean for n = 2 or 3 independent replicates. Source data are provided as a Source Data file.

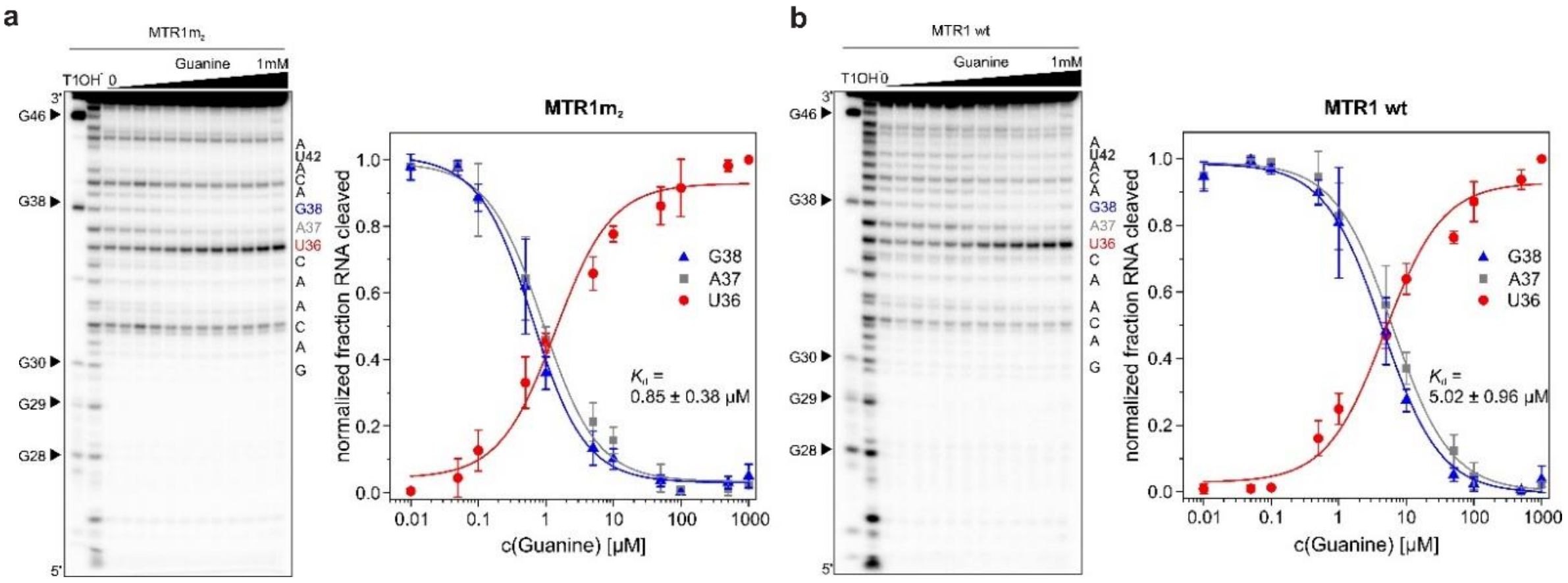

Figure S25. Representative PAGE images of in-line probing gels. PAGE gels of split MTR1m$_2$ (a) and MTR1wt (b) (pH 8.0, 20 mM $MgCl_2$, 20 °C, 36 h; m^{1}A-containing RNA-ribozyme complex, 0.01 – 1,000 µM guanine) together with analysis of normalized band intensities of G28, A37 and U36 fit to a one-site-binding model for determination of apparent K_d values. Error bars denote ± s.d. of the mean for n = 3 independent replicates. Source data are provided as a Source Data file.

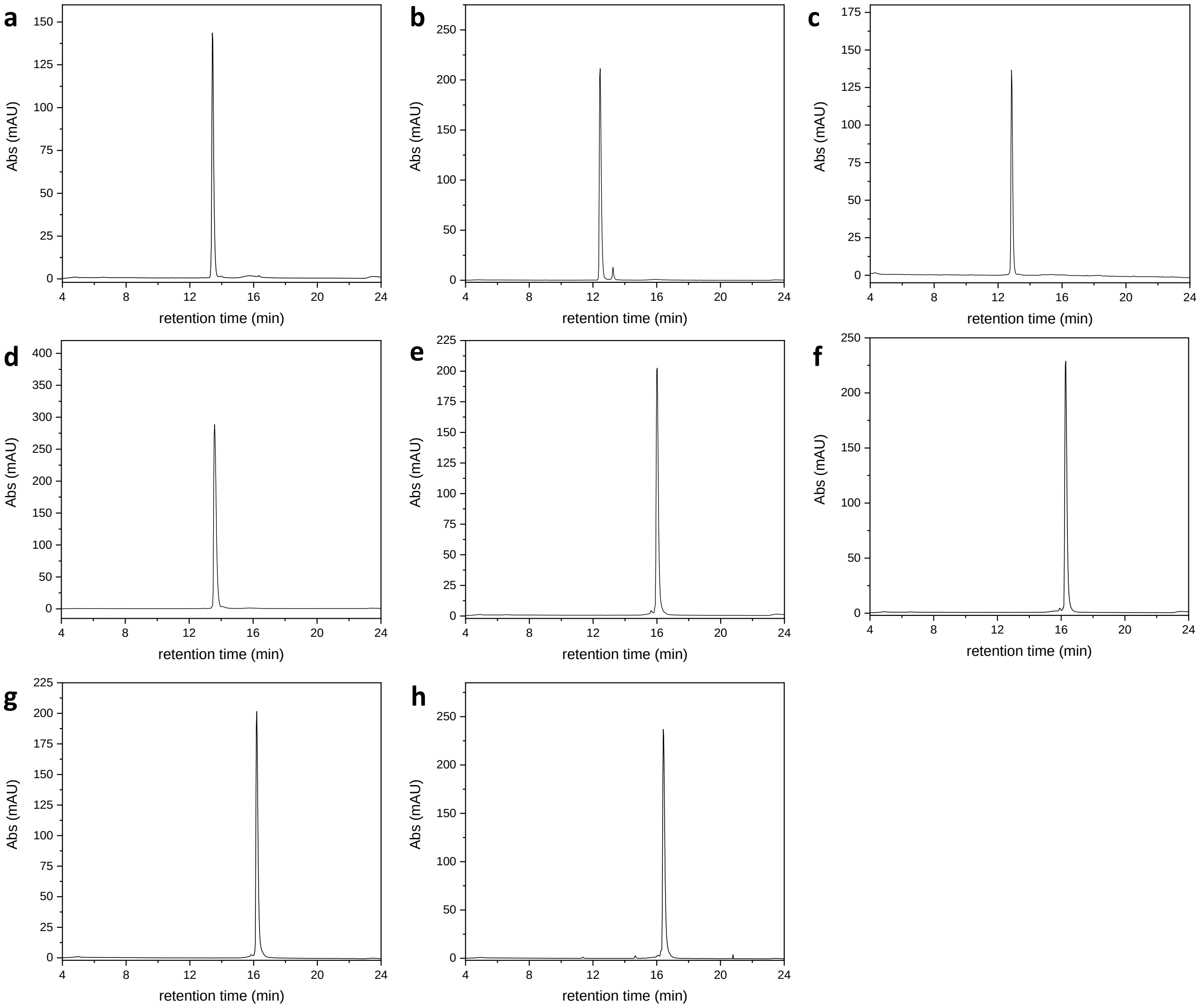

Figure S26. Anion-exchange HPLC analysis of utilized RNA oligonucleotides. RNA oligonucleotides were synthesized by solid phase synthesis (a) R1, (b) R1 m^1A, (c) R1 c^3A, (d), R1 c^7A, (e) R2 (OH), (f) R3 (OH), (g) R2 (OMe), (h) R3 (OMe).

Table S1. List of RNAs used in this study. Overview of RNA oligonucleotides prepared by solid phase synthesis together with their respective calculated and measured mass.

No.	description	5'-sequence-3'	Mass calculated	Mass found
R1	Substrate RNA	CCACUGAGAGCUUC	4403.63 Da	4403.63 Da
R1 m1A	Substrate RNA with m^1A	CCACUGm1AGAGCUUC	4417.61 Da	4417.65 Da
R1 c3A	Substrate RNA with c^3A	CCACUGc3AGAGCUUC	4402.60 Da	4402.63 Da
R1 c7A	Substrate RNA with c^7A	CCACUGc7AGAGCUUC	4402.62 Da	4402.61 Da
R2 (OH)	MTR1 5' fragment	GGAAGCUCUGACCGACCCCCAGCC	7621.13 Da	7621.11 Da
R3 (OH)	MTR1 3' fragment	GCUGGGACAACUAGACAUACAGUG	7735.10 Da	7735.13 Da
R2 (OMe)	MTR1 5' fragment Cm12	GGAAGCUCUGACmCGACCCCCAGCC	7635.11 Da	7635.17 Da
R3 (OMe)	MTR1 3' fragment Um42	GCUGGGACAACUAGACAUmACAGUG	7749.12 Da	7749.13 Da

Table S2. Data collection and refinement statistics.

	$MTR1m_2$ + m^6G
PDB code	9S1D
**Data collection**	
Wavelength (Å)	1.0332
Resolution (Å)	43.17-2.60 (2.72-2.60)*
Space group	$P\ 4_1\ 2_1\ 2$
Unit cell	
a, b, c (Å)	71.32 71.32 83.51
a, b, g (º)	90.0 90.0 90.0
Unique reflections	7084 (837)
Multiplicity	25.3 (27.0)
Completeness (%)	100 (100)
R_{merge}	0.044 (2.017)
R_{pim}	0.010 (0.396)
Mean I/sigma(I)	42.9 (2.0)
$CC_{1/2}$	1.0 (0.583)
Wilson B-factor (Å^2)	76.8
No. mol / ASU	1
**Refinement**	
Resolution (Å)	43.17-2.60 (2.98-2.60)
No. reflections for Rwork	6700 (2181)
No. reflections for R-free	347 (107)
Rwork / R-free (%)	22.0 /25.1
Coordinate error	0.36
No. non-hydrogen atoms	1374
RNA & ligand	1325
Ions / water	49
Average B-factor (Å^2)	44.34
Chain A (Å^2)	57.15
Chain B (Å^2)	46.40
Chain C (Å^2)	36.76
r.m.s. deviations	
Bond length (Å)	0.005
Bond angles (Å)	0.940
Clashscore	1.98
Number of TLS groups	3

*Statistics for the highest-resolution shell are shown in parentheses.

Table S3. Overview of observed rate constants k_{obs} for methyltransferase reactions performed using MTR1wt and MTR1m$_2$ at varying temperatures. Shown are k_{obs} values for each experimental replicate (rep.), the respective mean and standard deviation (SD). Reactions were performed under single turnover conditions using 1 µM substrate RNA and 10 µM ribozyme in the presence of 3 mM $MgCl_2$ and 100 µM m^{6}G at various temperatures, pH 6.0.

	MTR1 wt				MTR1m$_2$			
	k_{obs} [s^{-1}]				k_{obs} [s^{-1}]			
**Temp. (°C)**	**rep. 1**	**rep. 2**	**Mean k_{obs} [s^{-1}]**	**SD**	**rep. 1**	**rep. 2**	**Mean k_{obs} [s^{-1}]**	**SD**
**0**	2.78E-04	3.28E-04	3.03E-04	3.55E-05	3.89E-03	4.19E-03	4.04E-03	2.14E-04
**5**	3.72E-04	3.71E-04	3.72E-04	7.07E-07	5.56E-03	5.33E-03	5.45E-03	1.58E-04
**7**	3.80E-04	3.50E-04	3.65E-04	2.11E-05	5.68E-03	6.05E-03	5.86E-03	2.58E-04
**10**	3.76E-04	3.89E-04	3.82E-04	8.60E-06	6.49E-03	5.51E-03	6.00E-03	6.96E-04
**12**	4.00E-04	3.68E-04	3.84E-04	2.27E-05	6.97E-03	7.14E-03	7.06E-03	1.20E-04
**15**	4.07E-04	3.62E-04	3.85E-04	3.23E-05	7.17E-03	6.44E-03	6.81E-03	5.17E-04
**17**	2.73E-04	2.62E-04	2.67E-04	7.90E-06	5.66E-03	6.22E-03	5.94E-03	3.91E-04
**20**	2.25E-04	2.19E-04	2.22E-04	4.24E-06	5.42E-03	4.79E-03	5.11E-03	4.43E-04
**25**	1.76E-04	1.86E-04	1.81E-04	7.07E-06	3.66E-03	3.37E-03	3.52E-03	2.08E-04
**30**	0.78E-04	0.70E-04	0.74E-04	5.30E-06	2.57E-03	2.11E-03	2.34E-03	3.23E-04

Table S4. Fitting to Eyring equation. Fit values obtained from linear fit of temperature dependent MTR1wt and MTR1m$_2$ methyl transfer reactions (0 – 15 °C), and activation enthalpy ($\Delta H^{\ddagger}$) and entropy ($\Delta S^{\ddagger}$) calculated thereof.

	**MTR1 wt**	**MTRm$_2$**
**slope**	-894.73 ± 353.26	-2513.57 ± 472.25
**y-intercept**	-10.377 ± 1.26	-1.856 ± 1.68
**$\Delta H^{\ddagger}$ (kcal mol^{-1})**	1.78 ± 0.70	4.99 ± 0.94
**$\Delta S^{\ddagger}$ (cal mol^{-1} K^{-1})**	-67.8 ± 2.5	-50.9 ± 3.3
**$\Delta G^{\ddagger}_{298K}$ (kcal mol^{-1})**	22.0 ± 1.0	20.2 ± 1.4

Supplementary References

1. Harp, J. M. *et al.* Cryo neutron crystallography demonstrates influence of RNA 2'-OH orientation on conformation, sugar pucker and water structure. *Nucleic Acids Res.* **50**, 7721–7738 (2022).

2. Sabei, A., Hognon, C., Martin, J. & Frezza, E. Dynamics of Protein-RNA Interfaces Using All-Atom Molecular Dynamics Simulations. *J. Phys. Chem. B* **128**, 4865–4886 (2024).

3. Forget, S. & Stirnemann, G. Critical structural perturbations of ribozyme active sites induced by 2′-O-methylation commonly used in structural studies. *Nucleic Acids Res.* **54**, 13–14 (2026).

4. Mattelaer, C. A., Mattelaer, H. P., Rihon, J., Froeyen, M. & Lescrinier, E. Efficient and Accurate Potential Energy Surfaces of Puckering in Sugar-Modified Nucleosides. *J. Chem. Theory Comput.* **17**, 3814–3823 (2021).

5. Huang, M., Giese, T. J., Lee, T. S. & York, D. M. Improvement of DNA and RNA sugar pucker profiles from semiempirical quantum methods. *J. Chem. Theory Comput.* **10**, 1538–1545 (2014).

6. Darré, L. *et al.* Small Details Matter: The 2′-Hydroxyl as a Conformational Switch in RNA. *J. Am. Chem. Soc.* **138**, 16355–16363 (2016).

7. Scheitl, C. P. M., Mieczkowski, M., Schindelin, H. & Höbartner, C. Structure and mechanism of the methyltransferase ribozyme MTR1. *Nat. Chem. Biol.* **18**, 547–555 (2022).
