# Supplementary material for: A magic methyl effect in the active site of a methyltransferase ribozyme
Source: Nat Commun. 2026 Jul 17;17:8784. doi: 10.1038/s41467-026-75575-8 (PMC13493877; doi:10.1038/s41467-026-75575-8)
Supplement: Supplementary file 3 [file 41467_2026_75575_MOESM3_ESM.pdf]

nature portfolio

Peer Review File

A magic methyl effect in the active site of a methyltransferase ribozyme

Corresponding Author: Professor Alessandra Magistrato

This file contains all reviewer reports in order by version, followed by all author rebuttals in order by version.

Version 0:

Reviewer comments:

Reviewer #1

(Remarks to the Author)
The manuscript 'Magic methyl effect in RNA catalysis: how tandem ribose 2'-O-methylation in the active site accelerates RNA-catalyzed RNA methylation', authored by J. Aupic and coworkers, describes how an integrated multiscale computational approach – classical MD, QM/MM MD, and metadynamics – combined with new biochemical data and a 2.6 Å crystal structure of the doubly modified ribozyme (MTR1m2) was used to investigate the mechanistic basis of methylation-enhanced RNA catalysis. Their simulations reveal that cooperative hydrophobic interactions between the two neighboring 2'-OMe groups (at C12 and U42) rigidify the active site by locking the ribose rings in a C3'-endo conformation and excluding water from the catalytic pocket. This structural preorganization reduces the activation free-energy barrier relative to the unmodified ribozyme, closely matching independently measured kinetic effects. The newly solved crystal structure of the product state confirms the predicted 2'-OMe–2'-OMe interaction and the associated conformational stabilization, thus validating the computational findings.

Overall, the manuscript is well written and employs state-of-the-art computational techniques together with robust analytical tools, all of which are convincingly supported by carefully designed experiments. It therefore meets the expectations for publication in the targeted high-impact journal. While I cannot comment extensively on the experimental aspects, I provide below a set of specific comments on the theoretical part that I believe will further strengthen the manuscript.

i) Page 7, Results, first paragraph of the '2'-O-methylation of active-site ribose rings…' section, line 158: the authors state that 2'-OMe is known to stabilize the C3'-endo conformation. This point requires clarification. There are crystal structures of naturally occurring ribozymes in which 2'-OMe instead locks the ribose into the C2'-endo conformation (e.g., the hairpin ribozyme, PDB ID 2OUE). Thus, rather than stabilizing one specific pucker state, 2'-OMe generally suppresses dynamical repuckering and restricts the sugar ring to the conformation favored by the local structural environment. The authors should clarify this distinction.

ii) Page 8, in the following paragraph of the same section, the authors note that classical force fields struggle with accurate sugar-pucker description. This is correct, and it may be useful to emphasize that this limitation persists even in recent RNA force-field refinements (e.g., Refs. 10.1021/acs.jctc.1c00643; 10.1021/acs.jctc.3c00990), which highlight difficulty representing dynamic sugar-puckering accurately.

iii) Page 8, same paragraph (lines 175–176). Perhaps I somehow missed it, but how exactly was the 'active site' defined for these calculations? From which sugar-pucker state were the simulations initiated? Moreover, the last sentence of the paragraph (lines 184–185) seems to over-interpret the presented data. While the profile in Fig. S14b is reasonable, i.e., once 2'-OMe is introduced, the sugar pucker becomes locked, making C3'-endo/C2'-endo interconversion highly unfavorable and resulting in a large free-energy difference, the profile in Fig. S14a is more difficult to rationalize. The 2'-OH group should be able to sample both pucker states (as observed in standard MD simulations, e.g., Fig. 2), and one would expect a clear minimum corresponding to the alternative (C3'-endo) conformation. This suggests that the obtained free energy profiles could depend on how the 'active site' region was defined for the QM calculations and/or on insufficient relaxation of neighboring structural constraints during the limited simulation time.

iv) Page 8, in the 'Active site rigidification reduces…' section (lines 194–195), while I understand the authors' point, the data do not suggest that C2'-endo is clearly the preferred pucker state for U42 in the MTR1wt system based on the MD

simulations. Rather, it appears to adopt approximately a 50/50 distribution between C2'-endo and C3'-endo (Fig. 2). This raises the question of how the free energy profile might change if the QM/MM metadynamics calculations were initiated from the C3'-endo pucker instead… While I do not expect the authors to perform such computationally demanding simulations, they should at least comment on the potential impact of the chosen starting pucker on their results.

v) While I understand that the system size and complexity place practical limits on what can be simulated, I would appreciate a more detailed explanation of the obtained free energy surfaces (panels c and e) and their relationship to the corresponding profiles (panels d and f). Specifically, how were the one-dimensional profiles extracted from the two-dimensional surfaces, and how were the transition states identified on these landscapes? In panel c, for example, several additional minima appear on the surface, yet the corresponding profile seems to imply only one dominant reaction pathway. Conversely, panel even suggests the reaction may proceed through a single transition state, i.e., directly from the R to the P state.

Minor notes:
1. I would appreciate if the authors made the simulation and computational input files available in a public repository (e.g., Zenodo).
2. In Figure S11, the line in panel b is difficult to see. It would be preferable to enlarge the plot and present both lines (i.e., from panels a and b) together in a single, bigger plot.

Reviewer #2

(Remarks to the Author)
The manuscript by Aupic et al. describes the results of computational, biochemical and crystallographic analysis of an in vitro-evolved methyltransferase ribozyme. The crystal structure of the ribozyme as well as structure-guided biochemistry were published previously, leading to the observation that methylation of the 2'-OH of two residues in the active site resulted in improved catalysis. This manuscript explores the atomistic basis for this observation. Despite mention of "Magic" in the title, it appears that the main driver of improved catalysis is reduced conformational variability upon methylation. This is not unexpected, but in light of the abundance of 2'-O methylation in biological RNAs, and of the ready accessibility of this modification in synthetic RNAs, an atomistic analysis will be of interest broadly, and therefore I think the manuscript should be of interest to Nature Communications.

1. My main suggestion has to do with the presentation of the results. Throughout, in the molecular figures, the active site nucleases are shown. This is reasonable for a catalyst. However, what is being studied here is what is happening to the ribose upon methylation. Thus, the views in Figure S15 are the critical result, and this figure (or some variation) should feature prominently in the main text.

2. A crystal structure at 2.6 A resolution is being reported, and the main conclusion as to what drives the pucker preferences of the methylated ribozyme is that the methyl groups on residues 12 and 42 interact favorably. This is depicted schematically in Figure S15b. The electron density for the two (or three) residues shown in that panel needs to be shown in the main figure.

3. As to molecular driving forces, the authors misleadingly indicate that the methyl-methyl interaction is due to hydrophobic forces. Hydrophobic interactions are entropic, having to do with displaced water molecules. The favorable interactions are van der Waals (enthalpy). nomenclature aside, what is not described at all in the paper, but is critical for this line of argument, is the environment (polar, non polar?) of the 2'-OH or 2'-OMe of the two critical residues, in the two crystallographically characterized states. At a minimum, solvent-accessible (or maybe molecular, depending on what is more informative) surfaces for the area around the two residues in the two structures need to be shown in the main text for comparison.

4. To my point above, is it the hydrophobic effect (entropy) or the van der Waals interaction (enthalpy) of the two methyl groups (or both) driving the improved catalysis? I think that the way to answer this is to examine the enthalpy and entropy components of the activation energy of the reaction for the unmethylated and methylated ribozymes. Eyring analysis would be the standard experimental procedure. I think this would get to the crux of the matter.

Reviewer #3

(Remarks to the Author)
NCOMMS-25-89567 Magic methyl effect in RNA catalysis: how tandem ribose 2'-O-methylation 2 in the active site accelerates RNA-catalyzed RNA methylation by Aupič et al

This manuscript describes an investigation into an aspect of the in vitro-selected MRT1 methyl transferase ribozyme using computational calculation and X-ray crystallography. The results presented explore the observation of Höbartner et al that 2’-O-methylation of the C and U that hold the 6-methylguanine substrate accelerates the reaction, increasing the rate ~15 fold. The sugar pucker of the modified C and U nucleosides was analyzed by molecular mechanics. This suggests that the unmethylated ribose rings have a small fraction of C2’-endo pucker, while the methylated forms are exclusively C2’-endo. Quantum mechanical modeling of the active center suggested that 2’-O-methylation resulted in greater rigidity. The authors suggest that a hydrophobic interaction between the two methyl groups tightens the active center.

The crystal structure of the doubly-methylated form of MTR1 was solved. The structure is closely similar to the non-methylated form with an overal RMSD = 0.43 Å. In the methylated form the two methyl groups are directed towards each other with a C-C separation of 3.6 Å, thus slightly drawing together the ribose groups of C and U.
In the light of two previous published studies of MTR1 by molecular and quantum mechanical calculation, the advance in knowledge here is relatively small, and I don’t find the conclusions compelling. The new crystal structure is virtually identical to the earlier structures of the unmethylated RNA species, just showing that the methyl groups point at each other. Thus the new conclusions regarding MTR1 mechanism is somewhat incremental.

Version 1:

Reviewer comments:

Reviewer #1

(Remarks to the Author)
The authors have addressed most of my concerns, and the manuscript has improved in clarity and readability. From my perspective, it is now suitable for publication.

Reviewer #2

(Remarks to the Author)
The revised manuscript addresses all the concerns I raised, and I think is suitable for publication. I have two minor issues with the supplement: Fig S21, please indicate what kind of electron density map (simulated-annealing omit 2|Fo|-|Fc| would be the most obejctive, for an MR structure). Fig S22, I don't think a "hydrophobic interaction" can be inferred, unless water structure is known. As in the text, this should be a van der Waals interaction.

Open Access This Peer Review File is licensed under a Creative Commons Attribution 4.0 International License, which permits use, sharing, adaptation, distribution and reproduction in any medium or format, as long as you give appropriate credit to the original author(s) and the source, provide a link to the Creative Commons license, and indicate if changes were made.
In cases where reviewers are anonymous, credit should be given to 'Anonymous Referee' and the source.
The images or other third party material in this Peer Review File are included in the article’s Creative Commons license, unless indicated otherwise in a credit line to the material. If material is not included in the article’s Creative Commons license and your intended use is not permitted by statutory regulation or exceeds the permitted use, you will need to obtain permission directly from the copyright holder.
To view a copy of this license, visit https://creativecommons.org/licenses/by/4.0/

Point-by-point response to the reviewers' comments

Reviewer #1

The manuscript 'Magic methyl effect in RNA catalysis: how tandem ribose 2'-O-methylation in the active site accelerates RNA-catalyzed RNA methylation', authored by J. Aupic and coworkers, describes how an integrated multiscale computational approach – classical MD, QM/MM MD, and metadynamics – combined with new biochemical data and a 2.6 Å crystal structure of the doubly modified ribozyme (MTR1m2) was used to investigate the mechanistic basis of methylation-enhanced RNA catalysis. Their simulations reveal that cooperative hydrophobic interactions between the two neighboring 2'-OMe groups (at C12 and U42) rigidify the active site by locking the ribose rings in a C3'-endo conformation and excluding water from the catalytic pocket. This structural preorganization reduces the activation free-energy barrier relative to the unmodified ribozyme, closely matching independently measured kinetic effects. The newly solved crystal structure of the product state confirms the predicted 2'-OMe–2'-OMe interaction and the associated conformational stabilization, thus validating the computational findings.

Overall, the manuscript is well written and employs state-of-the-art computational techniques together with robust analytical tools, all of which are convincingly supported by carefully designed experiments. It therefore meets the expectations for publication in the targeted high-impact journal. While I cannot comment extensively on the experimental aspects, I provide below a set of specific comments on the theoretical part that I believe will further strengthen the manuscript.

i) Page 7, Results, first paragraph of the '2'-O-methylation of active-site ribose rings…' section, line 158: the authors state that 2'-OMe is known to stabilize the C3'-endo conformation. This point requires clarification. There are crystal structures of naturally occurring ribozymes in which 2'-OMe instead locks the ribose into the C2'-endo conformation (e.g., the hairpin ribozyme, PDB ID 2OUE). Thus, rather than stabilizing one specific pucker state, 2'-OMe generally suppresses dynamical repuckering and restricts the sugar ring to the conformation favored by the local structural environment. The authors should clarify this distinction.

We thank the reviewer for this comment. Our claim that 2'-OMe is known to stabilize the C3' endo conformation was referring to studies performed with model systems comprising single nucleosides. However, we agree with the reviewer this was not clearly stated. We have now included this clarification in the main text (page 7) and included a comment on the importance of the local structural environment in the Supplementary Discussion.

ii) Page 8, in the following paragraph of the same section, the authors note that classical force fields struggle with accurate sugar-pucker description. This is correct, and it may be useful to emphasize that this limitation persists even in recent RNA force-field refinements (e.g., Refs. 10.1021/acs.jctc.1c00643; 10.1021/acs.jctc.3c00990), which highlight difficulty representing dynamic sugar-puckering accurately.

We have emphasized this further in the main text (page 8) as suggested by the reviewer, including the suggested references.

iii) Page 8, same paragraph (lines 175–176). Perhaps I somehow missed it, but how exactly was the 'active site' defined for these calculations?

We employed the same definition of the active site (QM zone) across all hybrid QM/MM simulations (C12, U42, m^6G, A7). We have clarified this point in the Methods section (main text page 19) and

included an additional figure in the Supplementary Information showing QM atoms explicitly (Fig. S11).

From which sugar-pucker state were the simulations initiated?

For initiating QM/MM metadynamics simulations of sugar puckering we initially used the final frame of representative classical MD simulations. In case of MTR1wt, this resulted in C12 and U42 assuming the C3' endo and C2' endo state, respectively, at the start of the QM/MM metadynamics simulations, while in MTR1m$_2$, Cm12 and Um42 were both in the C3' endo state. We have included this information in the Methods section (main text page 19). During the revision stage, we have carried out an additional simulation for the MTR1wt system utilizing an alternative starting structure as described below.

Moreover, the last sentence of the paragraph (lines 184–185) seems to over-interpret the presented data. While the profile in Fig. S14b is reasonable, i.e., once 2'-OMe is introduced, the sugar pucker becomes locked, making C3'-endo/C2'-endo interconversion highly unfavorable and resulting in a large free-energy difference, the profile in Fig. S14a is more difficult to rationalize. The 2'-OH group should be able to sample both pucker states (as observed in standard MD simulations, e.g., Fig. 2), and one would expect a clear minimum corresponding to the alternative (C3'-endo) conformation. This suggests that the obtained free energy profiles could depend on how the 'active site' region was defined for the QM calculations and/or on insufficient relaxation of neighboring structural constraints during the limited simulation time.

We agree with the reviewer that the lack of a clear free energy minimum for the C3' endo state of U42 in MTR1wt (previously Fig. S14a) is surprising. To address the reviewer's concerns, we have extended the simulation for additional 10 ps to assess whether the shape of the free energy profile arises from insufficiently sampling of sugar puckering states. We observed no significant change in the free energy profile, suggesting that the sampling of sugar puckering states is sufficient. This is further supported by the graph depicting the U42 pseudorotational angle as a function of simulation time, now included in Fig. S15 (previously Fig. S14), that shows extensive sampling of both the C3' endo and C2' endo state. However, the reviewer is correct that QM/MM metadynamics timescales are too short to capture any larger structural rearrangements of the active site. The active site of MTR1wt is indeed quite flexible. For example, during classical MD simulations we observed an influx of water molecules in the active site and distancing of C12 and U42 2'-OH groups (Figs. S7 and S8). To test how the selection of the MTR1wt starting structure affects the free energy profile obtained from QM/MM metadynamics, we ran an additional simulation initiated from the relaxed crystal structure (pre-production MD), where C12 and U42 are both in the C3' endo state. The free energy profile extracted from this simulation shows a clear minimum for U42 in the C3' endo state. While the C2' endo state is still preferred, the difference in free energy is less pronounced ($\Delta F_{C3' \rightarrow C2'}$ of -6.1 ± 0.3 kcal/mol). This suggests sugar puckering preferences are heavily dependent on the larger structural environment. We have now included the results of this simulation in Fig. S15 and discuss their implication in the Supplementary Discussion section.

iv) Page 8, in the 'Active site rigidification reduces…' section (lines 194–195), while I understand the authors' point, the data do not suggest that C2'-endo is clearly the preferred pucker state for U42 in the MTR1wt system based on the MD simulations. Rather, it appears to adopt approximately a 50/50 distribution between C2'-endo and C3'-endo (Fig. 2). This raises the question of how the free energy profile might change if the QM/MM metadynamics calculations were initiated from the C3'-endo pucker instead… While I do not expect the authors to perform such computationally demanding

simulations, they should at least comment on the potential impact of the chosen starting pucker on their results.

We thank the reviewer for this comment. Indeed, we believe that the observed difference in the methylation free energy profiles obtained with QM/MM metadynamics for the MTR1wt and $MTR1m_2$ systems arises from different initial puckering of U42 versus Um42. Especially since the initial state persists during the QM/MM metadynamics simulations due to the reaction occurring on shorter time scales than sugar re-puckering in our simulations. By extension we posit that if MTR1wt simulations were initiated with U42 in the C3' endo state, the reaction free energy profile would be closer to that of $MTR1m_2$, with a lower rate-limiting activation barrier. We agree that classical MD simulations suggest U42 in MTR1wt can likely assume both C3' and C2'endo state in solution. This may be the reason behind the small difference between our calculated and experimentally observed rate acceleration upon introduction of 2'-O-methyl groups in U42 and C12. We have now adjusted the main text stating U42 in MTR1wt can likely assume both sugar puckers conformations (pages 15/16). We have also added a new section in Supplementary Discussion describing this point more extensively.

v) While I understand that the system size and complexity place practical limits on what can be simulated, I would appreciate a more detailed explanation of the obtained free energy surfaces (panels c and e) and their relationship to the corresponding profiles (panels d and f). Specifically, how were the one-dimensional profiles extracted from the two-dimensional surfaces, and how were the transition states identified on these landscapes? In panel c, for example, several additional minima appear on the surface, yet the corresponding profile seems to imply only one dominant reaction pathway. Conversely, panel even suggests the reaction may proceed through a single transition state, i.e., directly from the R to the P state.

We have obtained free energy profiles (panels d and f) from free energy surfaces (panels c and e) by calculating the lowest energy path from the reactants' to the products' free energy basin using the MEPSAnd program (Marcos-Alcalde, Iñigo, et al. *Bioinformatics* 36.3 (2020)). The maxima of these 1D free energy profiles were then identified as transition states. The 1D free energy profiles and activation free energies we obtained with this approach are in agreement with those previously calculated with umbrella sampling for the single-strand MTR1 ribozyme variant (McCarthy, Erika, et al. *Nucleic acids research* 51.9 (2023)).

While we agree with the reviewer that additional minima are present on the surface, all alternative reaction pathways were suboptimal and associated with higher activation free energy barriers. Among the top 5 pathways calculated with the MEPSAnd program, none proceeded through a single transition state for neither of the two investigated systems.

We have added a short comment in the main text (page 9) and included an additional Supplementary Figure (Fig. S17). The latter depicts the optimal and suboptimal reaction pathway on 2D free energy surface obtained for MTR1wt along with corresponding 1D free energy profiles and the optimal free energy pathway for the reaction in the $MTR1m_2$ system.

Minor notes:

1. I would appreciate if the authors made the simulation and computational input files available in a public repository (e.g., Zenodo)

We have deposited all classical MD trajectories and input files required to replicate all our simulations to Zenodo (doi: 10.5281/zenodo.18259069, temporary link:

https://zenodo.org/records/18259069?token=eyJhbGciOiJIUzUxMiJ9.eyJpZCI6Ijc5ZDRlODcyLTY2MWQtNDM5ZC05ZGU3LTAyMzcyNTBhYTQzYiIsImRhdGEiOnt9LCJyYW5kb20iOiJmMTAyMjE2ODA3OWViZmQyMDg2N2EzNTVjYmM5NzFjNiJ9.rR7Rj4B2jpyVhR5nM8byh_B5MTu4OpSXXcPyHDoRbkMm2_iplcwTWg2TqEvXv5zSYgGUa5VHVnb07hP_b3aWOQ). QM/MM MD and metadynamics trajectories are available from the corresponding author Alessandra Magistrato upon request, due to their large size.

2. In Figure S11, the line in panel b is difficult to see. It would be preferable to enlarge the plot and present both lines (i.e., from panels a and b) together in a single, bigger plot.

We have adjusted the figure (now Fig. S12) as suggested.

Reviewer #2

The manuscript by Aupic et al. describes the results of computational, biochemical and crystallographic analysis of an in vitro-evolved methyltransferase ribozyme. The crystal structure of the ribozyme as well as structure-guided biochemistry were published previously, leading to the observation that methylation of the 2'-OH of two residues in the active site resulted in improved catalysis. This manuscript explores the atomistic basis for this observation. Despite mention of "Magic" in the title, it appears that the main driver of improved catalysis is reduced conformational variability upon methylation. This is not unexpected, but in light of the abundance of 2'-O methylation in biological RNAs, and of the ready accessibility of this modification in synthetic RNAs, an atomistic analysis will be of interest broadly, and therefore I think the manuscript should be of interest to Nature Communications.

1. My main suggestion has to do with the presentation of the results. Throughout, in the molecular figures, the active site nucleases are shown. This is reasonable for a catalyst. However, what is being studied here is what is happening to the ribose upon methylation. Thus, the views in Figure S15 are the critical result, and this figure (or some variation) should feature prominently in the main text.

We thank the reviewer for this suggestion. To further underline how ribose methylation of the 2'-OH affected the sugar conformational behaviour in our MD simulations, we have now modified Figure 2 to include snapshots from classical MD simulations of MTR1wt and MTR1m$_2$, showcasing different sampled ribose conformations of C12/Cm12 and U42/Um42 (panel g).

2. A crystal structure at 2.6 A resolution is being reported, and the main conclusion as to what drives the pucker preferences of the methylated ribozyme is that the methyl groups on residues 12 and 42 interact favorably. This is depicted schematically in Figure S15b. The electron density for the two (or three) residues shown in that panel needs to be shown in the main figure.

We appreciate the reviewer's suggestion and have updated Fig. 5b with electron density maps (2Fo-Fc contoured at 1σ) for the active sites of both MTR1wt and MTR1m$_2$. All hydrogen bond interactions are indicated with black dashed lines. To avoid crowding, only the distances between the two oxygens or the two methyl groups are shown.

3. As to molecular driving forces, the authors misleadingly indicate that the methyl-methyl interaction is due to hydrophobic forces. Hydrophobic interactions are entropic, having to do with displaced water molecules. The favorable interactions are van der Waals (enthalpy). nomenclature aside, what is not described at all in the paper, but is critical for this line of argument, is the environment (polar, non polar?) of the 2'-OH or 2'-OMe of the two critical residues, in the two crystallographically characterized states. At a minimum, solvent-accessible (or maybe molecular, depending on what is more informative) surfaces for the area around the two residues in the two structures need to be shown in the main text for comparison.

We thank the reviewer for the correction of the terminology regarding the interaction between the two methyl groups. In our opinion, both hydrophobic interaction and van der Waals force engage in the magic methyl effect: the clustering of the two nonpolar methyl groups reduces the contact with the aqueous environment, providing an additional driving force to secure the active orientation, while the actual interaction between these two methyl groups is van der Waals forces. We have modified the text accordingly.

As for the description of the polar/nonpolar environment of the functional groups, we have incorporated it into our main text:

Page 12-13: A notable feature of the MTR1m$_2$ active centre is the two 2'-OMe groups pointing towards each other at a distance of 3.6 Å, situated at the regime of short-range van der Waals interactions. Additionally, compared to the polar hydroxyl groups in MTR1wt, the clustering of two weakly polar 2'-OMe groups in MTR1m$_2$ minimizes water contacts, which further bolsters their interaction via the hydrophobic effect.

To visualize the solvent accessible surface area (SASA) of the active sites in both structures, we added an additional figure (Fig. 6) showing the SASA of the active sites in both crystal structures. Combining the SASA and the water contact frequency observed during classical MD simulation (Fig S8), both data consistently support our observation that the higher solvent accessibility in MTR1wt is „sealed" off when two nonpolar methyl groups are installed (MTR1m$_2$), making the active centre less susceptible to water-induced destabilization.

4. To my point above, is it the hydrophobic effect (entropy) or the van der Waals interaction (enthalpy) of the two methyl groups (or both) driving the improved catalysis? I think that the way to answer this is to examine the enthalpy and entropy components of the activation energy of the reaction for the unmethylated and methylated ribozymes. Eyring analysis would be the standard experimental procedure. I think this would get to the crux of the matter.

We performed the suggested Eyring analysis by determining k_{obs} at various temperatures between 0°C and 30°C, and we observed a non-linear relationship for both the wild-type and the double-methylated ribozyme. In each case, we observed a linear increase of k_{obs} with rising temperatures between 0 - 15 °C, with an overall change by less than a factor of 2 for each ribozyme. The following 3- to 5-fold decrease of k_{obs} at higher temperatures likely reflects a combination of weaker hybridization (binding between substrate RNA and ribozyme) and destabilization of the H-bonding network within the ribozyme. Therefore, we only used data between 0 and 15°C for a linear fit to the Eyring equation, resulting in $\Delta H^{\ddagger}$ of 1.8 and 5.0 kcal/mol, and $\Delta S^{\ddagger}$ of -67.8 and -50.9 cal/molK for MTR1wt and MTR1m$_2$, respectively. Considering experimental uncertainty and error propagation the data should not be overinterpreted. However, these data tentatively suggest that improved catalysis is mostly due to entropic effects. In our simulations, we observed no significant change in active site solvation as the methylation reaction progressed for neither MTR1wt nor MTR1m$_2$, suggesting that the entropic cost is not primarily due to water ordering. Instead, our simulations suggest that the more favourable $\Delta S^{\ddagger}$ for MTR1m$_2$ arises from the more rigid, pre-organized active site that resembles the transition state (Fig. 5, Fig. S20). This rigidity is conferred by the van der Waals interaction between the two methyl groups of C12 and U42.

The results of the Eyring analysis are described in the text and Supplementary Discussion and show the data in the new Figure S23, Tables S3 and S4.

Reviewer #3

This manuscript describes an investigation into an aspect of the in vitro-selected MRT1 methyl transferase ribozyme using computational calculation and X-ray crystallography. The results presented explore the observation of Höbartner et al that 2'-O-methylation of the C and U that hold the 6-methylguanine substrate accelerates the reaction, increasing the rate ~15 fold. The sugar pucker of the modified C and U nucleosides was analyzed by molecular mechanics. This suggests that the unmethylated ribose rings have a small fraction of C2'-endo pucker, while the methylated forms are exclusively C2'-endo. Quantum mechanical modeling of the active center suggested that 2'-O-methylation resulted in greater rigidity. The authors suggest that a hydrophobic interaction between the two methyl groups tightens the active center. The crystal structure of the doubly-methylated form of MTR1 was solved. The structure is closely similar to the non-methylated form with an overal RMSD = 0.43 Å. In the methylated form the two methyl groups are directed towards each other with a C-C separation of 3.6 Å, thus slightly drawing together the ribose groups of C and U.
In the light of two previous published studies of MTR1 by molecular and quantum mechanical calculation, the advance in knowledge here is relatively small, and I don't find the conclusions compelling. The new crystal structure is virtually identical to the earlier structures of the unmethylated RNA species, just showing that the methyl groups point at each other. Thus the new conclusions regarding MTR1 mechanism is somewhat incremental.

While the reviewer is correct that the catalytic mechanism of the original non-modified MTR1 ribozyme has been resolved, all of which is clearly described and referenced in the main text, we feel the reviewer underestimated the relevance of our outcomes, which provide unique insights for future developments in the field of RNA catalysis. Specifically:

1. While the beneficial effect of 2'-OMe groups in MTR1 was already known, the underlying reason for the observed increase in catalytic rate upon introduction of two 2'-OMe groups was completely unknown prior to our current collaborative study. Here we demonstrate that the increase in catalytic activity is not due to a change in the reaction mechanism, but a consequence of an additional van der Waals interaction that reduces the ribose rings' conformational heterogeneity, rigidifies the active site and stabilizes the transition state. This is an important outcome that bears major significance for future ribozyme design. As a rule of thumb, current artificial ribozymes are weak catalysts ($k_{cat} < 2$ min^{-1}), and significantly less efficient in comparison to protein enzymes (Okuda *et al. Nat. Chem.* (2023), Krochmal *et al. Nat. Chem. Biol.* (2022)). For developing ribozymes with applicative potential in gene therapy or biochemical research, increasing their turnover rate is important. As the 3-helix junction structure is a common ribozyme scaffold, inclusion of a van der Waals interaction between 2'-OMe groups could prove a general strategy for increasing catalytic efficiency. Even more broadly, our results suggest that increasing the rigidity of ribozymes' active sites by whichever means and thus making them more protein-like will benefit future functional ribozymes.

2. While the reviewer agrees that the newly reported crystal structure of the modified MTR1 ribozyme confirms the positioning of 2'-OMe groups as suggested by our computer simulations, they conclude that changes to the active site are minor and therefore the result is not impressive. Years of work in the field of experimental and computational enzymology have shown that it is the precise positioning of active site atoms in the transition state that determines catalytic efficiency and even subangstrom-level deviations can completely abolish catalytic activity (Hilvert, Donald, *Annual Review of Biochemistry* 82.1 (2013)). Therefore, any active site perturbations are relevant to fully understand the factors that

mediate catalytic rates. This is particularly important for reliable construction of theozymes, i.e., theoretical catalytic sites, necessary for ribozyme design. Finally, the agreement between the active site as observed in QM/MM MD simulations and crystal structure provide evidence of the accuracy and precision of these methods for modelling ribozyme active sites, identifying them as a crucial tool for ribozyme design-oriented studies. We believe that due to the inherent plasticity of RNA molecules, the inclusion of dynamic aspects into the computational design process may be particularly beneficial.

We have modified the main text (pages 16, 17) to further emphasize the importance of our outcomes, as outlined in the points above.

REVIEWERS' COMMENTS

We thank both reviewers for evaluating our revised manuscript and their recommendations for publication.

Reviewer #1 (Remarks to the Author):

The authors have addressed most of my concerns, and the manuscript has improved in clarity and readability. From my perspective, it is now suitable for publication.

Reviewer #2 (Remarks to the Author):

The revised manuscript addresses all the concerns I raised, and I think is suitable for publication. I have two minor issues with the supplement:

Fig S21, please indicate what kind of electron density map (simulated-annealing omit 2|Fo|-|Fc| would be the most obejctive, for an MR structure).

The Figure caption of Figure S21 has been expanded with the relevant information.

We showed the 2Fo-Fc (1σ) and Fo-Fc (3σ) from a refinement [1] where the program had never seen any of the three methyl groups. Therefore, the three positive difference densities (green blobs) observed in S21b were least biased from the model phase compared to the typical omit maps calculated from the final model.

To compare S21b with different omit map methods as suggested by reviewer 2, a composite omit map was calculated with simulated annealing [2]. For comparison, we provide the 2Fo-Fc maps from the composite omit calculation and the original final refinement in Reply Figure 1. The composite omit map showed slightly less density around the methyl group on N^1-A7 and between Cm12 and G. The density around the two 2'O methyl groups has barely any difference between the two 2Fo-Fc maps. Overall, the composite omit map with simulated annealing validated the existence of the three methyl groups even in a less biased calculation.

Another omit map method generally used is manual removal of specific ligand/atoms from the final model and calculate difference density map (Fo-Fc) by refinement. We removed the three methyl groups from the final model and refined the omitted model, which yielded the 2Fo-Fc and Fo-Fc maps shown in Reply Figure 1b. The result is similar to Figure S21b with even more pronounced positive density peaks (green blobs) around the three methylated positions. This method, however, is more biased compared to the refinement map in Figure S21b since the final model has gone through multiple refinement rounds with installed methyl groups.

In summary, both the composite omit map with simulated annealing, and the standard omit map by refinement support our placement of three methyl groups in the reported MTR1m_2 structure.

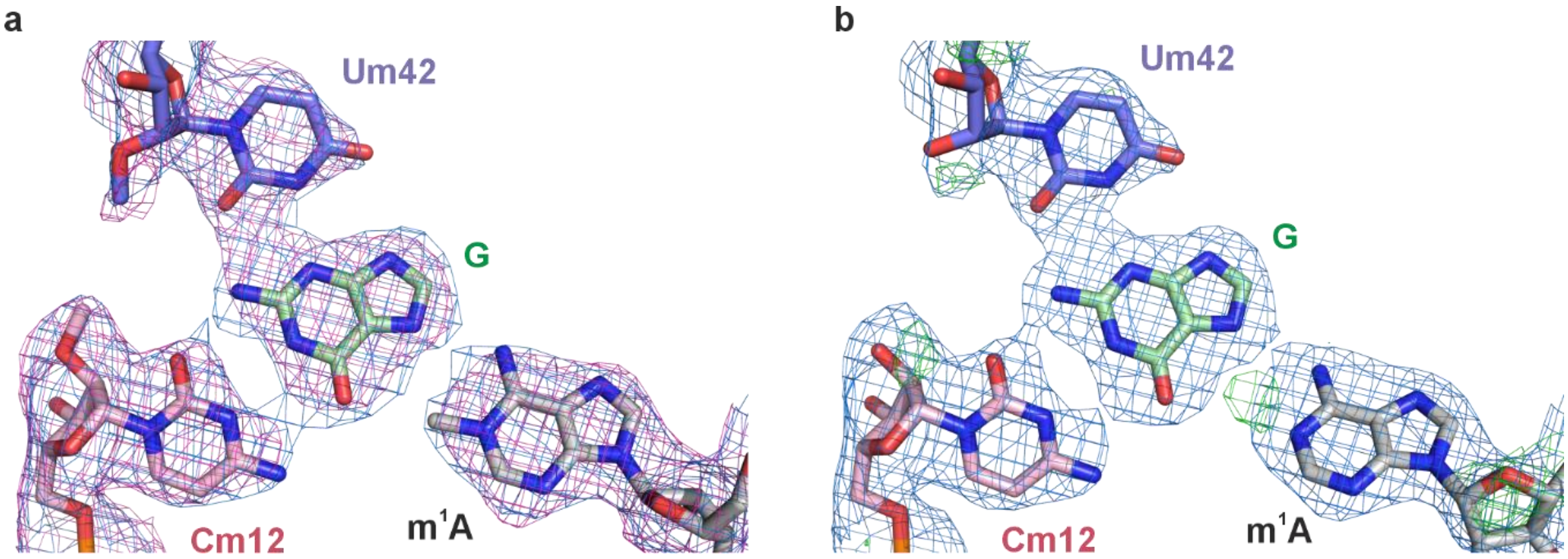

Reply Figure 1: Omit maps of the active site in MTR1m$_2$. (a). The overlay of two 2Fo-Fc density maps from the original refinement (blue) and the composite omit map calculation with simulated annealing (pink). Both maps are countered at 1σ. Note that composite omit map does not generate Fo-Fc difference map since it pools all individual omitted calculations into the final 2Fo-Fc map. (b). Standard omit map generated by refinement of triply unmethylated model. The blue and green mesh represent the σA-weighted 2Fo-Fc and Fo-Fc electron density countered at 1σ and 3σ respectively.

References:

1. Afonine, P.V. *et al*. Towards automated crystallographic structure refinement with phenix.refine. *Acta Crystallogr D Biol Crystallogr*. **68**, 352-67 (2012).
2. Terwilliger, T. C. *et al*. Iterative-build OMIT maps: map improvement by iterative model building and refinement without model bias. *Acta Crystallogr D Biol Crystallogr*. **64**, 515-24. (2008).

Fig S22, I don't think a "hydrophobic interaction" can be inferred, unless water structure is known. As in the text, this should be a van der Waals interaction.

The caption of Fig S22 was changed to mention only the distance between the 2'-O-methyl groups.
